# A supergene and genomic mosaicism are associated with phenotypic evolution in Galápagos tree finches

**DOI:** 10.1038/s41467-026-78507-8

**Published:** 2026-10-11

**Authors:** Matteo Sebastianelli Arbelaez, Erik D. Enbody, Carl-Johan Rubin, Carlos A. Valle, Lukas F. Keller, B. Rosemary Grant, Peter R. Grant, Leif Andersson

**Affiliations:** 1 Department of Medical Biochemistry and Microbiology, Uppsala University, Uppsala, Sweden; 2 SciLifeLab, Uppsala University, Uppsala, Sweden; 3 Department of Computational Biology, Cornell University, Ithaca, NY USA; 4 Colegio de Ciencias Biológicas y Ambientales, Galápagos Science Center GSC, Universidad San Francisco de Quito USFQ, Quito, Ecuador; 5 Department of Evolutionary Biology and Environmental Studies, University of Zürich, Zürich, Switzerland; 6 Department of Ecology and Evolutionary Biology, Princeton University, Princeton, NJ USA; 7 Department of Veterinary Integrative Biosciences, Texas A&M University, College Station, TX USA

**Keywords:** Adaptive radiation, Evolutionary biology, Comparative genomics

## Abstract

Adaptive radiations provide powerful models to study the origins of phenotypic novelty and species diversity. Darwin’s finches constitute a classic example of adaptive radiation, in which beak morphology is a key trait related to fitness. Here, we focus on *Camarhynchus* tree finches by analyzing whole genomes in combination with morphological data to revisit long-standing questions regarding their origins raised before genetic evidence became available. *Camarhynchus* diversified over the last 100,000 years, yielding some striking morphological differences despite shallow genetic divergence. We identify several highly differentiated loci, including *G03*, a supergene affecting beak and body size. We reveal a striking example of genomic mosaicism, in which autosomal differentiation between two sympatric species is restricted primarily to a single locus -(*G03*)- that approaches fixation, shifting them from one phenotypic optimum to another. Contextualized in a broader phylogenetic framework, we also reveal an unexpected diversity of *G03* haplotypes and show that the small and large *G03* alleles diverged early in the radiation, suggesting a key genetic innovation enabling ecological expansion among species of Darwin’s finches. Our results further establish *Camarhynchus* as a model example of speciation with gene flow, underscoring the broader role of hybridization in generating biodiversity.

## Abstract (web summary)

Adaptive radiations produce striking phenotypic diversity over remarkably short evolutionary timescales. This study uses genomic analyses of Darwin’s tree finches to reveal a cryptic lineage, in which autosomal differentiation with another sympatric species is restricted primarily to a single locus (G03) that approaches fixation, shifting them from one phenotypic optimum to another.

## Introduction

Adaptive radiations, consisting of groups of ecologically distinct species that evolved rapidly from a common ancestor, represent primary models to explore the mechanisms causing phenotypic innovation and speciation^1–3^. Growing evidence supports an important role for the reorganization of ancestral genetic variation in generating novel phenotypes underlying rapid diversification^4–9^. Lineage divergence can be further enhanced by introgression^10^, given that genetic variation resulting from hybridization increases the potential for phenotypic evolution, especially when introgressed regions contain loci that underpin traits that mediate simultaneously ecological interactions and contribute to non-random mating^11^. Yet, hybridization can also lead to lineage fusion by blurring genetic and phenotypic differences^12^, highlighting a subtle balance between its homogenizing and diversifying effects. Nonetheless, introgressed genes that evolved on different backgrounds have the potential to have large effects on phenotypes, and can strongly contribute to lineage diversification in sympatry^13,14^.

Darwin’s finches are an iconic example of recent adaptive radiation, in which 18 species distributed across the Galápagos archipelago and Cocos Island diversified over the one to two million years^15^. In this system, beak morphology and body size represent some of the fundamental phenotypic differences among species. These highly heritable traits are closely associated with diet and fitness^16,17^ and are linked with survival in periods of food scarcity during periodic droughts^3,18^. Concurrently, the same traits, together with song, are important cues involved in mate choice, and play a role in assortative mating^19,20^.

**Fig. 1:**
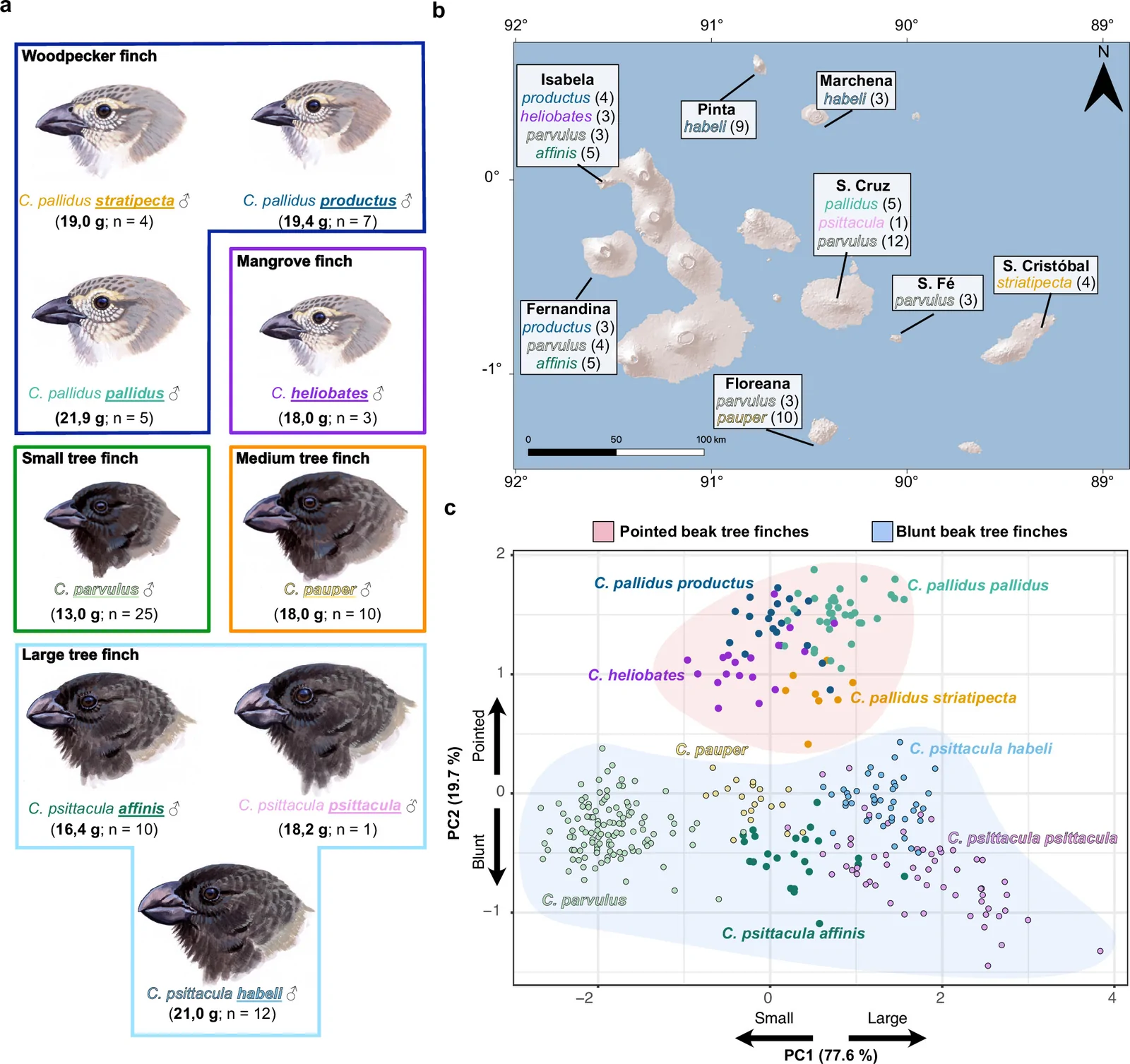
Galápagos islands, species distribution and phenotypes of *Camarhynchus* tree finches. **a** Head phenotypes, average body weight (g) and total number of genomic samples of the subspecies represented in this study, with their geographic distribution across the Galápagos archipelago shown in (**b**). The numbers indicate the number of whole-genome sequences per population. **c** PCA plot based on beak length, depth, and width measured from 348 museum specimens, reflecting morphological variation in beak measurements across tree finches, with PC1 reflecting beak size and PC2 reflecting beak shape. *C. pallidus* and *C. heliobates* show the least morphological differentiation, possibly reflecting relaxed competition in light of the allopatric geographic status among *C. pallidus* subspecies and strong ecological niche partitioning between *C. pallidus* *productus* and *C. heliobates*, which are therefore syntopic. On the contrary, species with widely overlapping distributions (*C. parvulus,* *C. pauper* and *C. psittacula*) show a high degree of morphological segregation. The signs of PC2 have been inverted with respect to Table S1 to ease figure interpretation. The taxonomic naming convention used in **b** mirrors that underlined in bold in **a**. Morphological data are from ref. ^22^. Bird illustrations by Szabolcs Kókay.

To date, much of our knowledge regarding the dynamics affecting survival, species interactions and gene flow among Darwin’s finches is based on a decades-long field and genetic study that focused on the *Geospiza* ground finches and specifically the inhabitants of Daphne Major island^3,17^. Like *Geospiza*, the Galápagos *Camarhynchus* tree finches diversified recently^15^. Early studies on the five tree finch species (Fig. 1a), currently distributed across most of the archipelago (Fig. 1b), were carried out to infer phylogenetic affinity within this group using morphological data^21,22^. More recently, tree finches were also embedded in the broader phylogenetic context of Darwin’s finches using modern methodologies^15^. Mirroring *Geospiza*, beak morphology in tree finches varies along two main axes: size (i.e. small vs. large) and pointedness (i.e. pointed vs. blunt) (Fig. 1c)^23,24^. It has previously been shown that the *G03* locus, a supergene controlling beak and body size^3^, is also associated with beak morphology in *Camarhynchus*^9,25^. Yet beyond that, we know little about the genomic architecture of beak size in Galápagos tree finches, which may have a different genetic basis in light of their distinct musculature and skull morphology compared to *Geospiza*^26^. Moreover, their evolutionary history has received much less attention than the *Geospiza* ground finches^27^, and attempts to describe the relationships among all species were only performed in the first half of the past century by David Lack^24^.

**Fig. 2:**
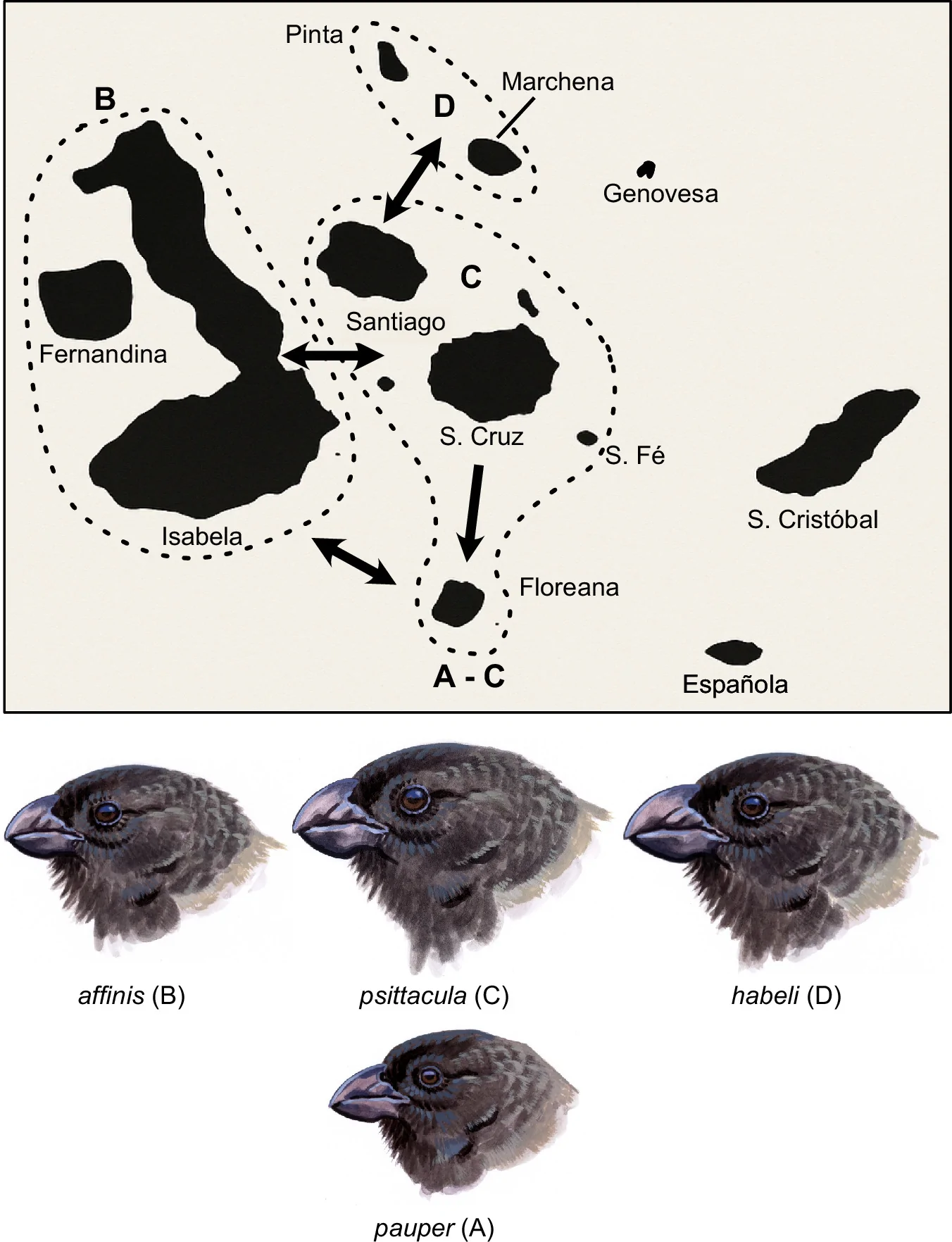
A possible speciation scenario between *C. pauper* and *C. psittacula* proposed by David Lack^24^. On Floreana, *C. pauper* (**A**) may have given rise to the *affinis* form (**B**) of *C. psittacula* (the smallest large tree finch in terms of beak and body size) on the western islands of Isabela and Fernandina, from which the nominate *C. psittacula psittacula* (with a large and blunt beak, **C**) evolved in the central island of Santa Cruz. *C. psittacula psittacula* populations then undertook different colonization routes, one towards the northern islands of Pinta and Marchena, where it developed into *C. psittacula habeli* (**D**), with a large but pointed beak, and another one southward back to Floreana, where *C. psittacula* *psittacula* established secondary contact with *C. pauper*. As *C. psittacula* became gradually larger in body and beak size as it spread across the archipelago, it had become so different from *C. pauper* that, upon secondary contact, the two lineages no longer interbred: phenotypic divergence allowed these two closely related lineages to coexist in sympatry. Uncertainty regarding colonization routes is reflected by the double-headed arrows. The map is redrawn from ref. ^24^. Bird illustrations by Szabolcs Kókay.

Only one island in the archipelago, Floreana Island, historically harbored three species of tree finches with blunt beaks. They are small, medium and large tree finch, with the latter long extinct as a breeding population and known only from museum specimens^28,29^. Focussing on the medium and large species, Lack^24^ argued that the large tree finch (*Camarhynchus psittacula*) is so similar to the same species on Santa Cruz Island it must have arrived from there, and recently (Fig. 2). He inferred that the ancestor of the medium tree finch species (*Camarhynchus pauper*), which is currently restricted to Floreana Island, must have arrived earlier and then evolved to its present form. Based on their phenotypic similarity, he then assumed that the *affinis* subspecies of large tree finch, currently classified as the smallest form of large tree finch, must be related to the medium tree finch on the western islands of Fernandina and Isabela—“*the Albemarle* (now Isabela) *form affinis shows close resemblance to pauper both in plumage and beak, so links up with it*”^24^—and evolved from it. However, Lack was unable to distinguish between alternative colonization routes and evolutionary histories throughout the archipelago, as is reflected in the double-headed arrows in Fig. 2. This is a good example of the opportunity to leverage evolutionary insight from genomic data to address questions that cannot be answered by phenotypes alone. Here, we revisit Lack’s thought-provoking speculations about the evolutionary dynamics of the tree finches to undertake a study of genomic and morphological variation within this system and meet the challenge. Even though we expand our investigation to the entire *Camarhynchus* phylogeny, we place particular emphasis on *Camarhynchus psittacula affinis*, currently described as a large tree finch but phenotypically more similar to the medium tree finch (Fig. 1c).

To investigate the genomic underpinnings of phenotypic evolution in the tree finch group, we build on a continuous whole-genome resequencing effort, currently comprising 415 Darwin’s finches^3,9,15^, encompassing all five *Camarhynchus* species and most of their currently recognized taxa (Fig. 1c). We show that tree finches are highly differentiated morphologically, yet genetic differentiation is minimal and confined to few genomic regions that include *G03* and other loci that were never reported before. We then show that *Camarhynchus psittacula affinis*, is an admixed lineage with a small tree finch autosomal background but with the *G03* haplotype for large body and beak size shared with large tree finches.

## Results

We used whole-genome sequencing data from 415 Darwin’s finches (including 77 *Camarhynchus* individuals) and in addition samples from two closely related outgroup species *Loxigilla noctis* (*n* = 3) and *L. barbadensis* (*n* = 5) from the Lesser Antilles. These individual genomes, with an average 15.8-fold sequencing depth (±8.2 sd), were aligned to the *Camarhynchus parvulus* reference genome^9^ resulting in the discovery of 11,222,039 biallelic single-nucleotide polymorphisms after filtering. These data form the basis of all analyses, unless noted otherwise. To improve readability when referring to the nine *Camarhynchus* lineages, we assigned each an epithet and use these throughout the manuscript hereafter (Table 1).

**Table 1:** Taxonomic naming convention of *Camarhynchus*

Species	Subspecies	Island	Epithet
*parvulus*	*parvulus*	All, except Pinta, Marchena and San Cristóbal	*parvulus*
*pauper*	NA	Floreana	*pauper*
*psittacula*	*affinis*	Isabela, Fernandina	western *psittacula*
*psittacula*	*psittacula*	Santa Cruz	central *psittacula*
*psittacula*	*habeli*	Pinta, Marchena	northern *psittacula*
*pallidus*	*striatipecta*	San Cristóbal	*pallidus* S. Cristóbal
*pallidus*	*pallidus*	Santa Cruz	*pallidus* S. Cruz
*pallidus*	*productus*	Isabela	western *pallidus*
*heliobates*	NA	Isabela	*heliobates*

Overview of the nine *Camarhynchus* lineages examined in this study, including species, subspecies, island of origin, and the epithets used for consistent reference throughout the manuscript.

### Evolutionary histories of the pointed-beak tree finches - the woodpecker (*C. pallidus*) and mangrove (*C. heliobates*) finches

**Fig. 3:**
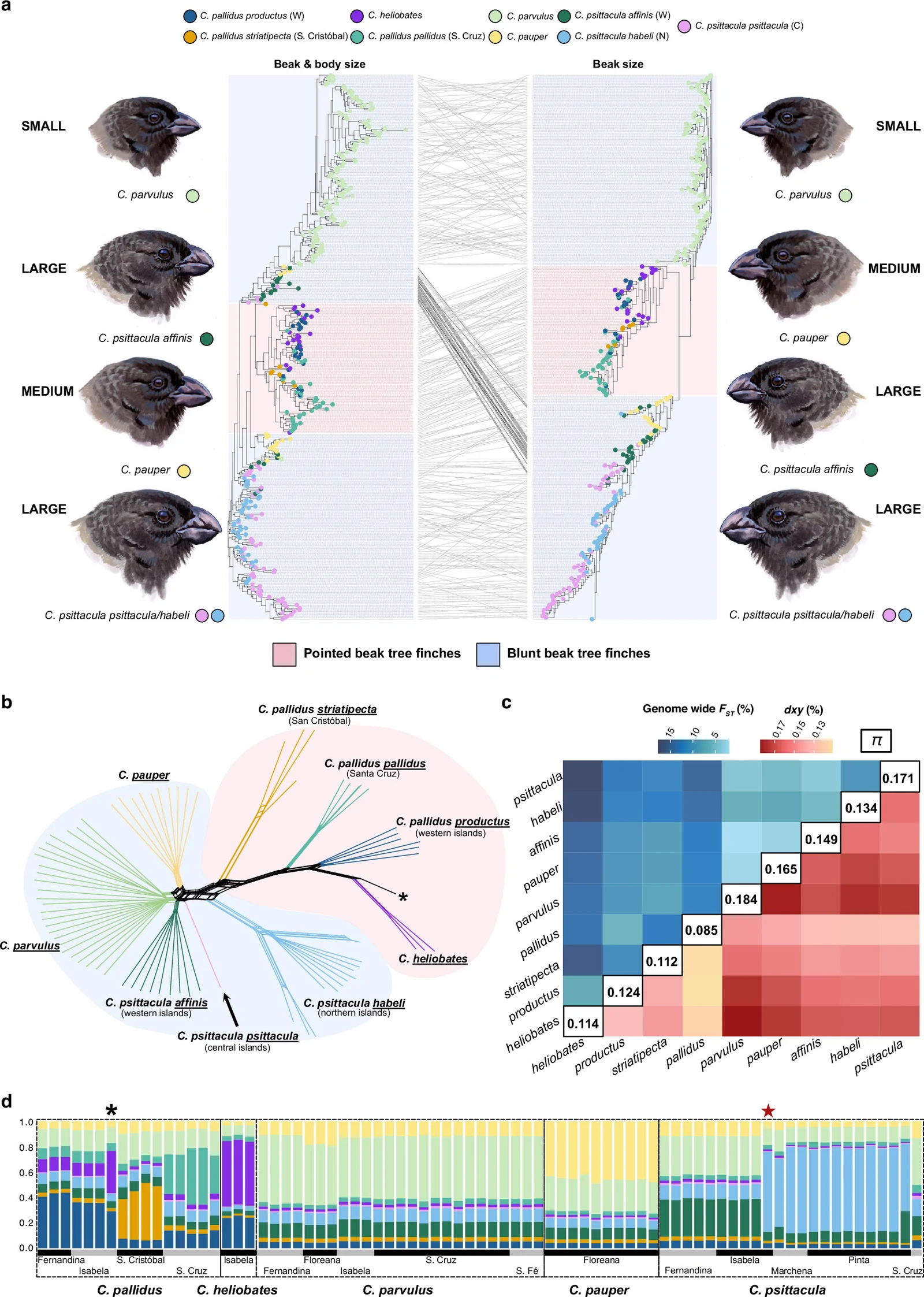
Evolutionary relationships, diversity and population structure in *Camarhynchus* tree finches. **a** Cladograms illustrating the relationship among *Camarhynchus* lineages based on morphology, with the left one being a cladogram built using both beak and body measurements and the right one using the beak measurements only. Remarkably, the western *psittacula* shows a closer relationship with *parvulus* based on overall morphology (left), but clusters with the other large tree finches based on beak measurements only (right), reflecting its current taxonomic classification. Tree finch icons mirror the position of the blunt-beak tree finches in each cladogram, highlighting the different position of the western psittacula between the two (also highlighted by the darker gray color of the lines connecting the same individuals between the two cladograms). **b** Phylogenetic network of the nine *Camarhynchus* subspecies analyzed in this study. **c** Representation of whole-genome pairwise diversity statistics (%) among subspecies, with the blue shades representing *F*_*ST*_ (upper panel), absolute divergence in red (*dxy*, lower panel) and within population nucleotide diversity (*π*) on the diagonal cells. The taxonomic naming convention used in (**c**) mirrors that underlined in (**b**). **d** Stacked bars reflecting proportions of genealogical nearest neighbor (GNN) assignments among tree finch taxa. The color of the stacked bars reflects the *Camarhynchus* taxa according to (**a**, **b**). The asterisk in (**b**, **d**) highlights 102Isa1940, a western *pallidus* × *C. heliobates* hybrid, whereas the red star indicates 106Isa8, likely a northern *psittacula* immigrant sampled on Isabela Island. Bird illustrations by Szabolcs Kókay.

The remarkable variation in beak size and shape is perhaps the most prominent characteristic of Darwin’s finches. Based on their morphological features, here summarized using a principal component analysis of beak and body measurements collected from 348 museum specimens (Fig. 1c, S1, Table S1), tree finches partition in morphological space in two main groups: the pointed-beak and the blunt-beak tree finches. This is also evident when building trees based on morphological trait distances (Fig. 3a). Genetically, these two groups are moderately differentiated with a pairwise genome-wide *F*_*ST*_ of 6%.

We investigated evolutionary relationships among tree finches by initially focusing on the pointed-beak group in three ways. First, by using a phylogenetic network approach (Fig. 3b) we found that *pallidus* San Cristóbal, *pallidus* Santa Cruz, western *pallidus* and *heliobates* are nested within each other, in a branching pattern that reflects their east to west geographic location. This network reflects pairwise levels of genome-wide relative diversity (Fig. 3c), with the western *pallidus* and *pallidus* Santa Cruz being the most closely related pair (genome-wide *F*_*ST*_ = 6.4%). Then, the western *pallidus* is the least divergent compared to *heliobates* (genome-wide *F*_*ST*_ = 7.2%), followed by *pallidus* Santa Cruz (genome-wide *F*_*ST*_ = 13.4%) and *pallidus* San Cristóbal (genome-wide *F*_*ST*_ = 16.0%). Altogether, our data support a split between pointed-beak and blunt-beak tree finches approximatively 100 Kya (Table S2). However, we caution that these estimate should be considered as minimal date, since our dating method based on pairwise net genetic distance^30^ makes no allowance for gene exchange (see *Methods*).

Second, we used ancestral recombination graphs to infer population structure. This analysis revealed that tree finch genomes are characterized by a mosaic of different ancestries, as summarized by genealogical nearest neighbor (GNN), a coalescent-based clustering method that summarizes the genome-wide relationship among all lineages (Fig. 3d). The GNN signatures of *C. pallidus* reflect their subspecies status. Similarly, *heliobates* shows a distinct genome-wide GNN signature. However, one western *pallidus* individual from Isabela (102Isa1940, marked with an asterisk in Fig. 3b, d) shows a mix of western *pallidus* and *heliobates* GNN components, suggesting hybrid ancestry. This is supported by its relatively high nucleotide diversity (*π* = 0.14%, Fig. S2) and chromosome-level GNN profile (Fig. S3). This result is not unexpected, since gene flow between *heliobates* and western *pallidus* has been documented in the recent past^31^. The hypothesis of gene flow is also supported by the results of an ABBA-BABA test (Table 2, Supplementary Notes 1, Fig. S4).

**Table 2:** Results of ABBA-BABA test for introgression

P1	P2	P3	*D*	*Z*	*P*-value	f4-ratio	%BBAA	%ABBA	%BABA
*pal*. Z.	*pal*. W.	*hel*.	0.08	9.05	<0.001	0.2	38.5	33.5	28.0

The test evaluates evidence of gene flow among *pallidus* Santa Cruz (*pal*. Z), western *pallidus* (*pal*. W), and *heliobates* (*hel*.), using *Loxigilla* sp. as outgroups. Reported statistics include the D-statistic (D), its corresponding Z-score (Z), and *P*-value, as well as the *f4*-ratio estimate of admixture proportion. The percentages of BBAA, ABBA, and BABA site patterns are also given.

Third, we analyzed measurements of museum specimens with normal mixture models (NMMs)^32^ to test whether our assessment of currently adopted taxonomic boundaries in the *Camarhynchus* genus is supported when morphological groups are defined without a priori information of their taxonomic origin. We estimated the percentage of individuals that were assigned to each morphological group, and demonstrate that *heliobates* and the three *pallidus* subspecies show ambiguity in their morphological assignments, reflecting overlap in morphology when using either all morphological measurements or only the beak measurements (Fig. S5).

### Evolutionary histories of the blunt-beak tree finches - small, medium and large

The small, medium and large tree finch (Fig. 1a), are all characterized by a blunt beak shape while showing considerable phenotypic variation in beak and body size, as implied by their common names (Fig. 1c, S1, Table S1). While the current taxonomy mirrors beak morphology, it does not reflect the overall morphology of beak and body size, with the western *psittacula* being similar to *parvulus* when considering beak and body size together (Fig. 3a).

From a genomic perspective, the unique population of *pauper* on Floreana Island belongs to a tight phylogenetic cluster with the western *psittacula* (genome-wide *F*_*ST*_ = 3%), *parvulus* (genome-wide *F*_*ST*_ = 2.5%) and central *psittacula* (genome-wide *F*_*ST*_ = 5.6%) (Fig. 3b-c). The tight relationship between *pauper* and *parvulus* reflects their close evolutionary origin, but it may also be affected to some extent by a documented history of recent gene flow between them^29,33–35^. This is reflected in the amount of lineage sharing between *pauper* and *parvulus* (Fig. 3d), with *pauper* having a relatively higher nucleotide diversity (Fig. 3c, Fig. S2) than it would be expected based on its population size^36,37^. Interestingly, *parvulus* and the western *psittacula*, which are currently classified as different species, are the least divergent among all tree finches, with a genome-wide *F*_*ST*_ = 1.7%, whereas the conspecific western and northern *psittacula* are the most divergent pair within the blunt-beak tree finches (genome-wide *F*_*ST*_ = 9.6%). However, we caution that the different nucleotide diversity between western and northern *psittacula* may partially inflate the *F*_*ST*_ estimate.

Altogether, blunt-beak tree finches have a very close evolutionary relationship, yet their beak and body size is significantly partitioned in morphological space, as revealed by our NMM analysis (Fig. S5). This provides an opportunity to investigate the genetic architecture of such recent divergence. Therefore, we calculated genomic differentiation genome-wide to identify loci underpinning phenotypic differences among tree finches by calculating interspecies nucleotide divergence (i.e.* dxy*) and *F*_*ST*_ in 10 kb non-overlapping windows for all *Camarhynchus* taxa. *dxy* scans (Fig. S6) reveal some chromosomes with regions of relatively high *dxy*, though these divergence peaks are present in all the comparisons, indicating shared ancestral variation. For instance, a divergence peak on chromosome 11 likely results from the translocation of a 380 kb region on the W chromosome (see Fig. S7 and Supplementary Notes 2). However, by using *F*_*ST*_, a measure of relative differentiation, we show that that genetic differentiation among blunt-beak tree finches is mostly confined to a few loci on chromosomes 1A (in correspondence with *G03*), 5, 6 and Z (Figs. S8a–11, Table S3 and Supplementary Notes 3 for a detailed description of the gene content at *F*_*ST*_ peaks). However, when correcting *F*_*ST*_ by its genome-wide average (Z*F*_*ST*_), one autosomal locus (i.e.* G03*) stands out, with relative differentiation at this locus between the western *psittacula* and *parvulus* being ~25 SD relative to the genome background (Fig. S8b).

### The western psittacula is a mosaic lineage

**Fig. 4:**
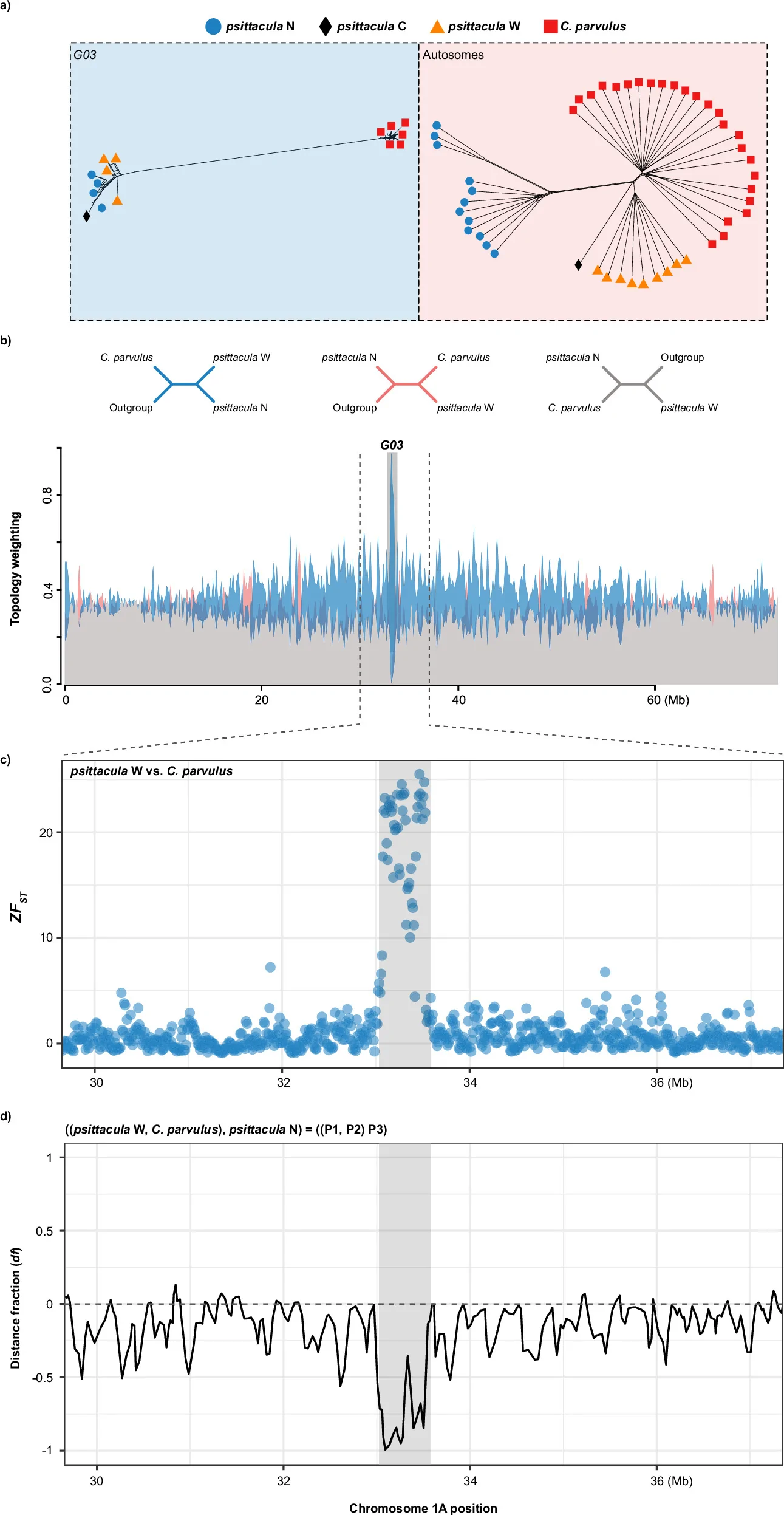
Shared *G03* alleles between northern and western *psittacula.* **a** Phylogenetic networks based on 4,602 sites across the core region of *G03* (left panel) and across autosomes after removal of *G03*. **b** TWISST tree topology weighting analysis across chr. 1A revealing full support for the topology in which the northern and western *psittacula* are monophyletic (blue topology) instead of that in which the western *psittacula* and *parvulus* are sister species (red topology). The *Loxigilla* sp. are used as outgroups. The support for the blue topology peaks within *G03*, an outlier locus that shows a strong deviation from the genome-wide *F*_*ST*_ average between western *psittacula* and *parvulus*, as highlighted by the *ZF*_*ST*_ plot in (**c**). Also, by analyzing local patterns of allele sharing, we report evidence of an excess of shared alleles between northern and western *psittacula* at *G03* as shown in (**d**), in which negative values of the distance fraction (*df*) support extensive allele sharing between northern *psittacula* and western *psittacula*, whereas positive *df* values would reveal evidence of gene flow between northern *psittacula* and *parvulus*. Location of the *G03* locus is indicated by gray shading.

*F*_*ST*_ divergence scans (Fig. S8) show that the western *psittacula* and *parvulus* are likely to be fixed for different *G03* alleles (while also showing strong divergence on chromosome Z, Fig. S11) despite the shallow levels of autosomal genetic differentiation. This was confirmed by a species tree formed by western *psittacula* and northern *psittacula*, whereas western *psittacula* are much more similar to *parvulus* across autosomes (after removal of *G03*, Fig. 4a). Interestingly, the placement of the single individual of central *psittacula* mirrors that of the western *psittacula* within the networks, suggesting a very close relationship between them, but the limited sample size of the central *psittacula* prevents us from comparing their local patterns of genomic differentiation. The genetic similarity of *parvulus* and western *psittacula* outside *G03* (but not within) is further corroborated by a tree topology weighting analysis across chromosome 1 A (Fig. 4b). We also show that in the region of the elevated Z*F*_*ST*_ between *parvulus* and western *psittacula* (i.e. *G03*, Fig. 4c), the ABBA-BABA-based distance fraction (*df*)^38^ supports allele sharing at this locus between large tree finches (northern and the western *psittacula*, Fig. 4d). Altogether, these complementary analyses suggest that western *psittacula* is a mosaic lineage, i.e. in which admixed genomes retain structured ancestry rather than homogeneous despite gene flow over generations. It is closely related to *parvulus* and *pauper* but being fixed for the *G03 large* (*L*) haplotype.

To further explore the evolutionary history of this mosaic population, we calculated the nucleotide diversity at the *G03* locus and *dxy* in comparisons with other species/populations of Darwin’s finches (Fig. S12). This analysis allowed us to refute a scenario in which introgression of the *G03 L* allele originates from a single founder, because the *G03* nucleotide diversity is as high as in other populations with a high frequency of the *L* allele. These data rather support a scenario with gene flow from the abundant *parvulus* population to a rarer large-beaked population that has homogenized most of the genome outside the *G03* locus and that selection maintains the phenotypic difference between the two sympatric populations. Furthermore, in light of their sequence similarity, this analysis suggests that the *L G03* allele of the western *psittacula* may originate from a pointed-beak tree finch rather than from a *psittacula*. The *pauper* population is segregating for the *G03 L* and *S* alleles, but the *dxy* data suggest that its *L* alleles are more similar to the ones in nominate *psittacula* than to the ones in the other tree finches with large beaks (Fig. S12).

Further, an in-depth investigation of *df* patterns genome-wide revealed that, besides *G03*, two other regions show an increase of shared alleles (i.e*. df* < *−*0.75) between northern and western *psittacula* (Fig. S13a). One is located on chromosome 2, a locus also highlighted in Rubin and Enbody et al.^9^ that includes three forkhead box genes (i.e*. FOXC1, FOXF2* and *FOXQ1*) and *EXOC2*, with the former playing an important role in palate and craniofacial development^39–41^ (Fig. S13b, left panel). The other locus is located on chromosome 7 and includes *SATB2* (Fig. S13b, right panel), another homeobox gene also involved in bone development and craniofacial patterning^42^.

### Admixture mapping reveals loci underlying genomic differentiation among tree finches

Rubin et al.^9^ previously performed admixture mapping comparing the *parvulus*, *pauper* and *psittacula* species and only a single locus, *G03*, stood out as highly differentiated. Based on our finding of a mixed ancestry of the western *psittacula* we decided to revisit this question and exclude this taxon. Thus, we performed pairwise comparisons of the three *Camarhynchus* groups that are morphologically most differentiated: the pointed-beak tree finches (whose limited sample size prevented us from investigating genomic differences at the population level), *parvulus* and *psittacula* (excluding the western *psittacula* considering its admixed autosomal background). Admixture mapping highlighted once more the importance of the *G03* locus, the region with the strongest association in every comparison between *parvulus* with either *psittacula* or the pointed-beak tree finches, reflecting their difference in beak and body size (Figs. S14, S15). Interestingly, admixture mapping revealed additional divergent loci between *parvulus* and *psittacula* at chromosomes 2, 3, 5, 7 and Z after excluding western *psittacula* from the comparison (Fig. S14a). These divergent loci were not evident when including western *psittacula* in the large tree finch group due to homogenization of genome-wide differences (Fig. S14b). We also recovered a divergent locus on chromosome 6 where the pointed-beak tree finches carry a unique allele (Fig. S15), while a divergence island on chromosome 2 further differentiates the two large lineages (Fig. S15b): pointed-beaked tree finches and *psittacula* (see Supplementary Note 4 and Table S4 for a detailed description). In summary, these analyses resulted in identification of 15 not previously reported quantitative trait loci (QTLs), denoted *C01*-*C15*, contributing to genetic differentiation among *Camarhynchus* species.

### Extensive haplotype variation at *G03* among Darwin’s finches

**Fig. 5:**
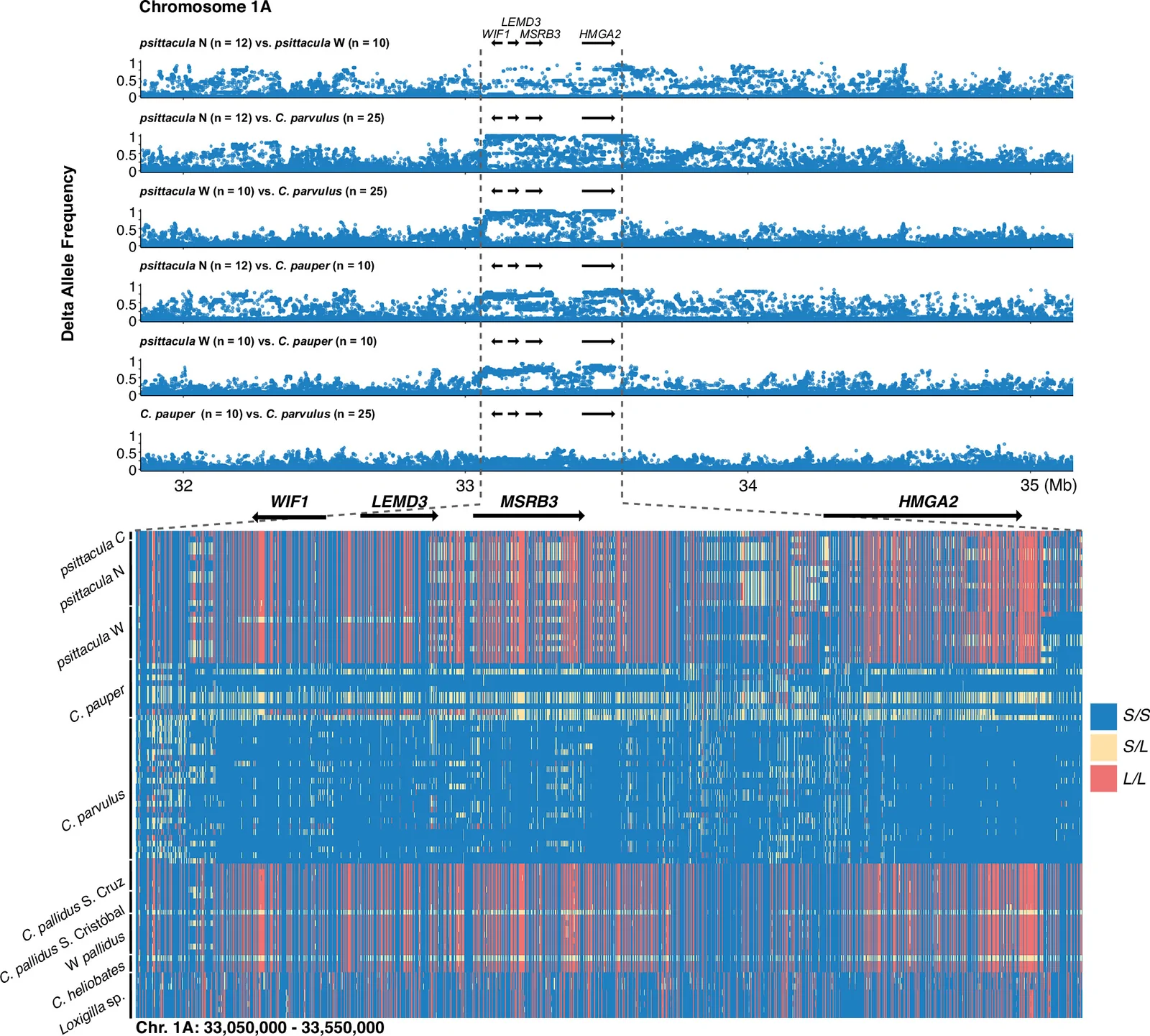
Delta allele frequencies and genotypes at the chromosome 1A *G03* locus underlying differences in beak and body size. The upper panel shows differences in allele frequency among *C. psittacula, C. pauper* and *C. parvulus* subspecies. The bottom panel highlights individual haplotypes within the core 500 kb region of this locus^9^ as well as the relative position of the genes found therein. *C. pallidus, C. heliobates* and the *Loxigilla* sp. outgroups are also included. Blue genotypes indicate homozygosity to the *C. parvulus* small beak (*S*) haplotype (*L* indicates the large beak haplotype). Altogether, the figure illustrates that allele frequency differences at *G03* between species with different beak and body size are underpinned by different haplotypes that segregate by species.

Our analyses suggest that the *G03* allele frequency differences between *parvulus* and the three *psittacula* subspecies (Fig. 5, upper panel) are underpinned by haplotypes that segregate by species. In fact, all *psittacula* carry the *L* haplotype at *G03*, a haplotype shared with *pallidus* and *heliobates* (Fig. 5, bottom panel). As expected, all *parvulus* individuals are homozygous for an *S* haplotype, while the *L* and *S* alleles segregate in *pauper* as they do in *G. fortis* (medium ground finch). This variation is consistent with the important role of the *G03* supergene for phenotypic diversification among Darwin’s finches. Notably, the similar phenotypes of the western *psittacula* and *pauper* (Fig. 1, S1) have a different genetic architecture, with that of the former being underpinned by homozygosity of the *L* allele at *G03* in conjunction with other *parvulus* loci, whereas in *pauper G03 S* and *L* alleles segregate.

**Fig. 6:**
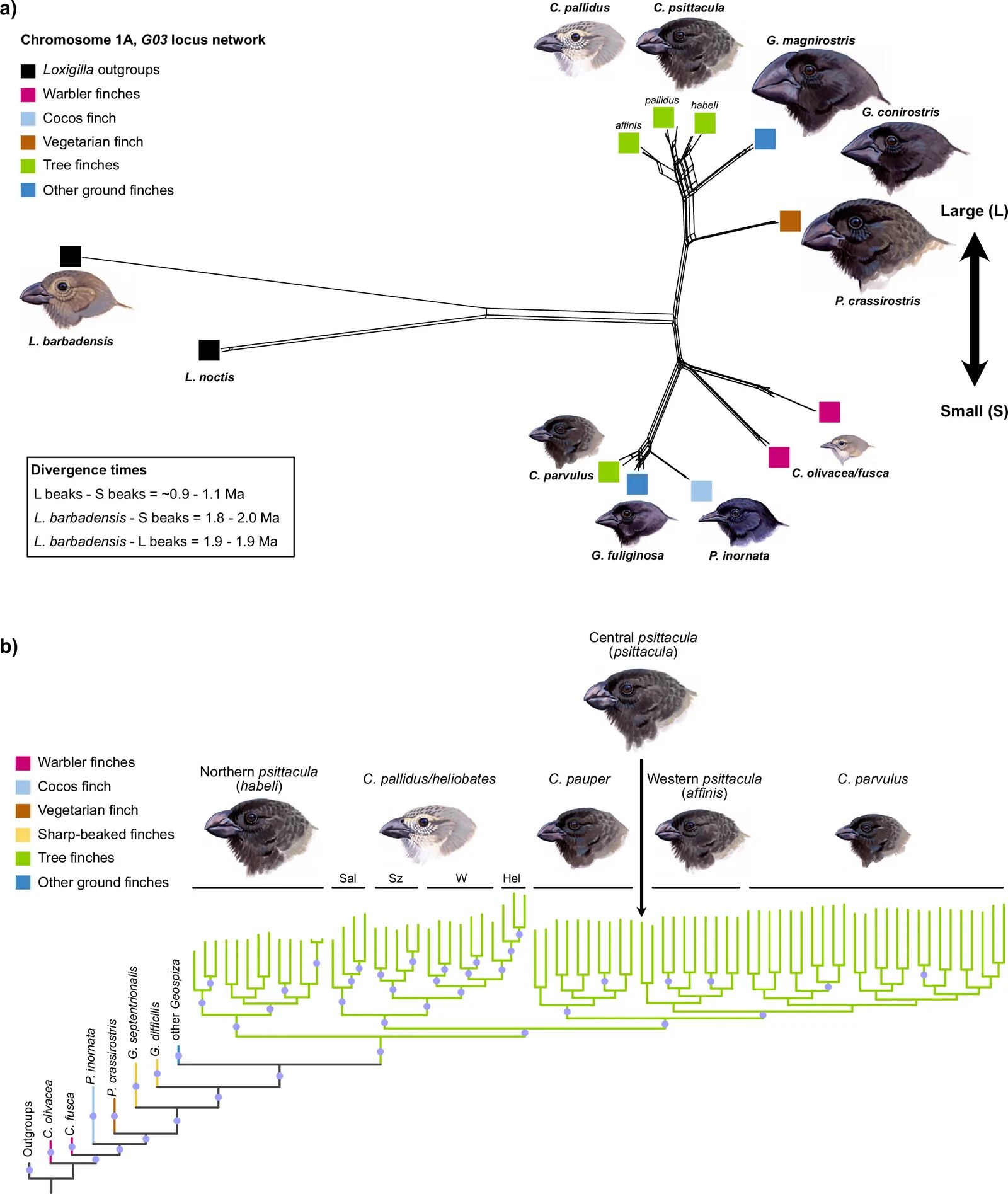
Local and genome-wide phylogenies of Darwin’s finches. **a** Haplotype network for the *G03* locus for Darwin’s finches with either large or small beaks. The divergence time between the small (S) haplotypes of *G. fuliginosa* and *C. parvulus* and the large (L) haplotype of the ground finches is indicated in the box. **b** Whole-genome maximum-likelihood phylogeny, violet circles demark fully supported nodes. Notations within the *C. pallidus/C. heliobates* group are as follows: Sal = *pallidus* San Cristóbal, Sz = *pallidus* Santa Cruz, W = western *pallidus* and Hel = *heliobates*. Bird illustrations by Szabolcs Kókay.

We visually confirmed the presence of the *L* and *S* haplotypes by contextualizing the tree finch haplotypes with that of other species in the radiation. This revealed the presence of multiple *G03* haplotypes in both large and small beaked finches (Fig. S16), which we further validated by building a haplotype network of the core region of the *G03* locus (Fig. 6a). By focusing on species with either small or large beaks, we found several large (*L*) beak haplotypes—one in the ground finches (large ground finch *Geospiza magnirostris* and Española cactus finch *G. conirostris*), one in the vegetarian finch (*Platyspiza crassirostris*) that is likely a recombinant in light of its shorter genetic distance with the *S* beak haplotype of *C. parvulus* (*dxy* = 0.43%) compared to the distance between the latter and that of *G. magnirostris* (*dxy* = 0.52%) (Figs. 6a, S12) and three in the tree finches. Similar to the large *G03* alleles, we also found variation in the small beak haplotypes. In fact, two are unique to the warbler finches (*Certhidea olivacea* and *C. fusca*), whereas another is shared among Cocos (*Pinaroloxias inornata*), small tree (*C. parvulus*) and small ground finches (*G. fuliginosa*). Altogether, the split between the *L* and *S* haplotypes predates the split between warbler finches and other Darwin’s finches. By dating the divergence time of the large and small *G03* haplotypes, we confirm that the split between these haplotypes occurred early in the radiation, from 900 Kya to 1.1 Mya. According to dating restricted to the *G03* locus, the Cocos finch diverged from the other finches more recently than suggested by the whole-genome phylogeny (Fig. 6b). In fact, based on the topology of the *G03* locus, the taxonomic placement of Cocos finch is much closer to the small ground and tree finch.

We also investigated how selection has affected the *G03* genomic region (525 kb) by comparing nucleotide diversity (*π*) within the *G03* region with genome average for all species of Darwin’s finches, including two outgroup species (Fig. S17). There is a clear trend that *π* is lower in *G03* compared with genome average, also in the outgroup species. This most likely reflects the low recombination rate in the *G03* region, which implies that background selection tends to reduce nucleotide diversity in this region possibly in combination with selection for beak and body size^43^. Only four species had higher *π* in the *G03* region than genome average, *G. conirostris*, *G. septentrionalis*, *C. pauper* and *C. heliobates*, because they segregate for the *G03* S and L alleles. Two species had extremely low *π* in the *G03* region, *G. magnirostris* and *G. acutirostris*, indicative of selective sweeps.

### Tree finches in the phylogenetic history of Darwin’s finches

To place our inference of the evolutionary history of tree finches within the broader Darwin’s finch radiation, we built a maximum-likelihood phylogeny from our updated genomes and revised divergence times across the tree (Fig. 6b). This phylogeny is broadly consistent with that of previous studies^15^. According to estimated divergence time (Table S2, Fig. S18 for pairwise *dxy* values among finch groups) *Certhidea* warbler finches and the ancestor of all other species diverged ~850 Kya (Table S2), followed by Cocos finch (~500 Kya, but see Fig. 6a) and vegetarian finch (~480 Kya). All the sharp-beaked ground finches form a basal lineage that diverged from the rest of the radiation ~380 Kya, before the split between tree and ground finches, with the latter two being the most recent major split that occurred at least 360 Kya (Table S2).

As for *Camarhynchus*, the maximum-likelihood phylogeny (Fig. 6b) also supports a division of the large tree finches into two groups. In fact, the northern *psittacula* is the earliest diverging lineage of all tree finches, whereas the western and central *psittacula* form a clade that shares a common ancestor with the small tree finch *C. parvulus*. The latter three form a monophyletic group with *C. pauper*. This scenario is completely inverted in the Z-linked phylogeny (Fig. S19), however, where *C. pauper* occurs as sister lineage to the western *psittacula*, and *C. parvulus* forms two separate clusters.

## Discussion

Our study has revealed very close evolutionary relationships within the *Camarhynchus* phylogeny of Darwin’s finches. We provide evidence that the western *psittacula*, described as the smallest form of large tree finch, is a mosaic lineage, thus underscoring the importance of gene flow as a major driver of phenotypic innovation in Darwin’s finch phylogeny. It also underscores the key role of the *G03* supergene for phenotypic diversification among tree finches. David Lack proposed that the large tree finch, western *psittacula*, evolved from the medium tree finch, *C. pauper* (Fig. 2). We now show that the small (*parvulus*), medium (*pauper)* and large (western *psittacula*) tree finches are very closely related (pairwise *F*_*ST*_ in the range 1.7% to 9.6%) and the greatest genetic differences between them are that *parvulus* is fixed for the *G03 S* allele, *pauper* is segregating for *S* and *L,* while western *psittacula* is fixed for the *L* allele. Altogether, our findings highlight the challenges of assigning discrete taxonomic boundaries in a system showing close evolutionary relationships and gene flow.

### Genetic relationships among *Camarhynchus* tree finches

Interspecies nucleotide divergences (*dxy*) among tree finches are only slightly higher than intraspecies nucleotide diversities (*π*), with pairwise *F*_*ST*_ values between taxa ranging from 2% to 15% (Fig. 3c), a level of genetic differentiation usually observed among subpopulations within species. The extensive allele sharing complicates the reconstruction of their evolutionary history, resulting in a poor correspondence between whole-genome phylogeny and the current morphology-based taxonomy (Fig. 3a, b). A recent study proposed that *pallidus* San Cristobal should be recognized as a distinct species^27^, and our data suggest that a similar argument could be made for the northern *psittacula*. Indeed, our maximum-likelihood phylogeny (Fig. 6b) suggests *psittacula* is polyphyletic: with the northern populations on Pinta and Marchena sharing one ancestor, while central and western populations share another. Therefore, one might regard the northern *psittacula* as a separate species, *C. habeli*, distinguished by beak and body size and, to a lesser extent, by male plumage—traits that led Sclater et al. to describe it as distinct^44^. However, its genome-wide differentiation from other blunt-beaked tree finches is low (*F*_*ST*_ < 10%), indicating a recent origin, and the deeper split of northern *psittacula* could reflect hybridization among other blunt-beaked lineages. Moreover, it is worth noting that in spite of their genetic similarity, *parvulus* and *psittacula* have different feeding ecology^26^ and seldom interbreed when sympatric. The latter evidence, contrary to the previous ones, supports their status as different species.

Equally complicated are the taxonomic relationships among pointed-beak tree finches, where *heliobates* is a *pallidus* relative that is specialized to mangrove habitats and is paraphyletic among *pallidus* subspecies. However, the ongoing hybridization between *pallidus* and *heliobates* violate the assumptions of the phylogenetic hypothesis that groups these populations on our phylogenomic tree. The pointed-beak tree finches show population structure and higher genetic differentiation than blunt-beaked finches but are morphologically less distinct, a pattern that is consistent with their current taxonomy. These findings demonstrate the limitations of taxonomic classification solely based on morphology or genomic data without the possibility to integrate behavioral observations^45^. We emphasize that issues of taxonomic classification should be treated with caution among tree finches. Our results call for a field study on the ecology and mating patterns of *psittacula* and the possibility of hybridization of the pivotal *psittacula* populations (i.e. central and western) as well as incorporating additional samples into the phylogenomic analyses.

### The key role of the *G03* supergene for beak and body size in Darwin’s finches

We estimate that the diversification leading to modern tree finches occurred within the last 100,000 years, followed by substantial morphological divergence despite shallow levels of genetic differentiation except at seven main loci on four chromosomes, including the *G03* supergene on chromosome 1A, which shows the highest deviation from the genome-wide average (Fig. S8b). Previously known as a key locus underlying a fitness-related trait associated with granivory in *Geospiza* finches^3^, our study support previous evidence^9,15^ that *G03* is a recurrent driver of beak and body size evolution across distinct ecological niches of Darwin’s finches. The study has uncovered a considerable diversity of *G03* haplotypes associated with beak size across the Darwin’s finch radiation, which is a hallmark of supergenes and other adaptive haplotypes, that they evolve over time by accumulating multiple mutations affecting fitness^46^. The maintenance of the *G03 S* and *L* alleles as large haplotype blocks over the last million years (Fig. 6a) implies the presence of multiple causal variants across this 525 kb genomic region with no evidence for the presence of an inversion^3^.

*G03* is involved in a striking example of genomic mosaicism, in which the western *psittacula* combines a *Large G03* haplotype with an otherwise predominantly *parvulus* autosomal genome, resulting in shallow genetic differentiation between the two (pairwise *F*_*ST*_ = 1.7% genome-wide), with clear differentiation at only a few loci on chromosome Z and one autosomal locus, *G03*. Notably, our data suggests that the source of the *Large G03* haplotype of the western *psittacula* may be a pointed-beak tree finch in light of the sequence similarity between the former and both *C. pallidus* and *C. heliobates* (Figs. 6a, S12). Thus, based on genomic evidence, the current taxonomic classification of western *psittacula* as a large tree finch is questionable. Morphologically, the beak of the western *psittacula* is medium-sized, ~25% larger than *parvulus* (Fig. 1c), thus closely matching the effect size of *G03* on beak-size variation in ground finches estimated in a previous GWAS^3^. This suggests that allelic variation at this locus likely explains most of the morphological differentiation between these lineages. We propose that the mosaicism has been created by repeated introgression of alleles from *parvulus* to a large beak population of tree finches. There are no field observations of interbreeding on Isabela or Fernandina Island between *parvulus* and *psittacula*, but Lack^23^ suggested hybridization may explain the difficulty in classifying some specimens from these islands.

Mirroring its genetic mosaicism, the morphology of the western *psittacula* is slightly disproportionate (i.e. a larger beak compared to its body size, Fig. 3a). Indeed, its medium-sized beak results from combining fixation, or near fixation, of a large beak *G03* haplotype with *parvulus* alleles at other loci. However, the body size of western *psittacula* does not overlap with that of *C. parvulus* and is larger (but, on average, smaller than other *psittacula* subspecies and *C. pauper*; see body weight in Fig.1a, size in Fig. S1). This difference may be attributed to several physiological and structural adaptations that enable a small-bodied bird to support a large beak. Even though *G03* includes *HMGA2*, a gene known to affect body size^3^, we highlight *the follistatin* gene (*FST*) located on chromosome Z (known for its pleiotropic effects, including regulation of body size^47^) as a putative candidate further contributing to size differences between the western *psittacula* and *parvulus* in light of many fixed differences located upstream of *FST* (Fig. S11). However, many small effect loci, undetectable with the current sample sizes, may also contribute to these phenotypic differences in body size.

### The evolutionary relationships among Darwin’s finches

Our phylogenetic analysis, based on genome-wide data, largely confirms the established relationships among Darwin’s finch species (Fig. 6b). However, we observed that the Cocos finch appears more closely related to the ground and tree finches based on the local *G03* phylogeny compared to the genome-wide tree (Fig. 6a). This placement agrees with previous mitochondrial DNA, W and Z-linked phylogenies (Fig. S19)^15^, suggesting a more recent split than inferred from autosomal data (i.e. ~ 500 Kya, Table S2). Lack speculated that Cocos finch may originate from the sharp-beaked finches in the northern islands (*G. septentrionalis* or *G. difficilis*), and a phylogeny based on chromosome Z supports this hypothesis^24^.

Compared to the major groups of Darwin’s finch radiation, the relationships within the genus *Camarhynchus* are less clear. In fact, our phylogenetic reconstruction places the northern *psittacula* basal to all species in this genus (Fig. 6b). Thus, this result is in disagreement with Lack’s hypothesis of the northern *psittacula* originating from central *psittacula* (Fig. 2). However, consistent with his view, the *C. pauper* split occurs before differentiation of central *psittacula* and *parvulus* (Fig. 6b), and the close relatedness between western *psittacula* and *pauper* is also supported by the similarity of their *G03* haplotypes (Fig. S12). Even though we attempted to retrace the evolutionary steps of the blunt-beak group, it is challenging, even with genomic data, to reconstruct island colonization routes in these species, given their dynamic evolution and frequent hybridization, especially because of varying historical connectivity between the central islands^48^.

Altogether, the *Camarhynchus* radiation provides a neat example of speciation with gene flow and emergence of a mosaic species fueled by introgression. More broadly, our findings contribute to a growing body of evidence that hybridization and divergence are not mutually exclusive processes, but can interact to generate evolutionary novelty during the early stages of speciation, whether in bacteria^49^, insects^50^ or other vertebrates^10,11,51–54^. A significant finding of this study has been a similar role of the *G03* locus in the evolutionary history of both tree finches and ground finches. However, while previous studies have highlighted *G03* as a major driver of beak size evolution, those examples exhibit substantial genome-wide differentiation and the involvement of additional loci^9,25^. In contrast, the western *psittacula* example is remarkable because two sympatric species occupy distinct phenotypic optima while differing primarily at this single locus. In fact, despite the sister relation between the western and central *psittacula*, the phenotype of the western lineage has been heavily modified by introgression from *parvulus*, whereas the morphology of the latter appears to have been less affected. Considering their very recent origin and close relatedness, *Camarhynchus* tree finches represent a natural experiment—a system in which the earliest stages of divergence can be studied. Despite the challenges regarding species delimitations they pose, tree finches illustrate that by embracing evolutionary complexity, we can gain a far better understanding of the processes generating biodiversity.

## Methods

### Sample collection

The collection of blood samples, funded by the National Science Foundation (NSF), was conducted with annual permits from the Galápagos National Parks Directorate, with approval from Princeton University’s Animal Care Committee and in accordance with its protocols and supported logistically by the Charles Darwin Research Station in Galápagos. Blood samples from Darwin’s finches were collected across the Galápagos archipelago as part of past sampling efforts^9,15,25^, with blood extracted from the brachial vein of the birds, preserved in *EDTA*-soaked filter paper and then stored in DRIERITE. Sampling was conducted in accordance with the protocols of Princeton University’s Animal Welfare Committee.

### DNA extraction, library preparation

In this study, we used 415 Darwin’s finch samples (including the 77 *Camarhynchus* individuals that are the major focus of this study) and 8 *Loxigilla* outgroups that were processed as part of previous DNA extraction and sequencing efforts^3,9,15,55^, with the exception of two *C. psittacula* individuals that were added in this study. Briefly, we extracted DNA from blood stored on filter paper by following a previously established protocol^55^. Libraries for whole-genome sequencing were generated using a custom Tn5 transposon-based protocol^3,9,55,56^ adapted from Picelli et al.^57^, and sequencing for the two *C. psittacula* individuals sequenced in 2024 was performed using the NovaSeq X Plus platform (Illumina, CA).

### Whole-genome variant calling

For our downstream bioinformatic pipeline, we used Sentieon tools^58^, a rewritten GATK algorithm that improves temporal efficiency of the BWA/GATK pipeline while producing identical output. Specifically, we aligned reads to the Camarhymchus_parvulus_V1.1 genome assembly (GCA_902806625.1) using the *bwa-mem* module v0.7.18 provided therein^59^. We further used Sentieon to call variants using the Haplotyper algorithm and perform joint genotyping using the GVCFtyper modules, which are analogous to HaplotypeCaller and GenotypeGVCFs from GATK 4.1^60^. We further used VariantFiltration (GATK 4.3.0.0) to perform the following quality filters: QD < 2.0, FS > 60.0, SOR > 3.0 MQ < 40.0, MQRankSum < −12.5, DP < 2 and DP > 100. The filtered variants were then set to no-call using SelectVariants (GATK 4.3.0.0). The resulting VCF file was further filtered using *bcftools* v1.2^61^ to retain biallelic SNP and with *vcftools* 0.1.16^62^ to remove sites with a minor allele frequency threshold of 0.03 and maximum missingness of 0.75 (i.e. 25%). These filtering parameters were chosen for consistency with previous Darwin’s finch studies^3,9^.

### Relative sequence depth sexing

In avian systems, males have two copies of the Z chromosome, while females have only one Z copy and one W. Therefore, to infer individual sex, we compared the relative depth of the Z chromosome in each individual to the respective autosomal depth, with the expectation that males would have a similar Z chromosome to autosome depth ratio, whereas females would have half the depth on the Z chromosome compared to autosomes.

### Ancestral recombination graph inference

The VCF file was phased in *shapeit* v4.1.3^63^ using a previously generated *Camarhynchus parvulus* recombination map^9^. The phased VCF was then used to infer ancestral recombination graphs (ARGs) using *tsinfer* 0.4.0^64^, in which we provided information on the ancestral allele based on allelic frequencies of the two *Loxigilla* outgroups. Ancestral nodes were then dated using a bird mutation rate of 2.04^−9^per site per year^9,15^ using *tsdate*^65^.

### Population structure and patterns of population divergence

Given their power to capture the full coalescent history of each genomic region while accounting for recombination, ancestral recombination graphs (ARGs) are very powerful tools to infer genetic admixture and introgression^66^ and are increasingly being used in both humans^65,67,68^ and non-model systems^69,70^. Therefore, our inferred ARGs were used to compute, for each individual, the cumulative distribution of the genealogical nearest neighbors (GNN) statistic^64^. For each focal individual, the GNN algorithm traverses the trees along the genome and, at each locus defined by the *tsinfer* algorithm, identifies the genealogically nearest neighbors—that is, the samples that share the most recent common ancestor with the focal haplotype. We then recorded the population labels of these nearest neighbors. The GNN statistic for each individual was calculated as the proportion of genome-wide trees in which the nearest genealogical neighbors belonged to each of the predefined populations (i.e. the nine *Camarhynchus* taxa). This yields a probability distribution over populations, reflecting the relative frequency with which an individual’s local ancestry coalesces most recently with members of each population.

We further calculated *F*_*ST*_, a measure of relative differentiation, in 10 kb windows by using the tree sequence toolkit *tskit* (https://tskit.dev/), and specifically the *ts.F*_*ST*_ *()*, which leverages the *ts* tree sequences previously generated in *tsinfer*. We used pixy^71^ to estimate between species (*dxy*) and within species (π) nucleotide diversity in 10 kb windows by using a VCF file that included both invariant and variable sites. To perform these diversity statistics, two individuals were removed, the first one being the putative *C. p. pallidus* × *C. heliobates* hybrid individual (102Isa1940) and the second being the *C. psittacula* 106Isa8, a northern *psittacula* immigrant individual originally classified as western *psittacula*.

To obtain a per-site resolution of the landscape of divergence, we calculated delta allele frequency (an approach commonly used to visualize allelic differences between populations^72,73^) by extracting allelic frequencies from each focal population using the *–freq* flag in *vcftools* and then calculating the absolute difference between the reference alleles of each population pair being compared. However, when sample size is small, this approach results in Manhattan plots with increased background noise. In light of this, it was not possible to calculate delta allele frequency among taxa within the *C. pallidus *- *C. heliobates* group.

### Divergence time estimation among *Camarhynchus* populations

We estimated pairwise divergence splits among *Camarhynchus* taxa based on autosomal data. Pairwise divergence times among taxa were calculated using the number of nucleotide substitutions while accounting for the intrapopulation nucleotide diversity (*dx* and *dy*) as *da = dxy* − *(dx + dy)/2*^30^. Time since divergence would then be calculated as *T = da/2μ*, with *μ* being the mutation rate of 2.04 × 10^−9^ per year for consistency with previous studies^9,15^. To obtain average split times, we performed these calculations on 10,000 randomly selected windows (10 kb each) and performed this operation 100 times. This was done to account for uncertainty related to *dxy, dx* and *dy*. However, since we cannot account for uncertainty in mutation rates, we only report the average divergence time and not the associated confidence interval. We opted for this approach for estimating lineage divergence time because it uses net sequence divergence (*da*) to estimate splits without relying on fossil calibration and because it accounts for shared variation by subtracting nucleotide diversity. However, this method is sensitive to recent gene flow since *T* is directly related to *da* and *dxy*, and therefore estimates should be interpreted as minimal time splits.

We also estimated divergence times between beak haplotypes at the *G03* locus across finches with either large or small beaks. Since this approach leverages *dxy* and nucleotide diversity, our initial minor allele frequency threshold of 0.03 on a VCF with 459 individuals likely removed variants that are unique to the outgroups (*n* = 8) and Cocos finch (*n* = 8), resulting in an underestimation of divergence times. To prevent this, we extracted individuals from the unfiltered VCF to retain all the outgroups, Cocos finch and a subset of *C. pallidus, C. psittacula, C. magnirostris, C magnirostris, P. crassirostris, C. parvulus* and *G. fuliginosa* for a total of 100 individuals. This dataset was then filtered with a minor allele frequency of 0.01 and maximum-missingness of 0.75. We then estimated *dxy* and nucleotide diversity in windows containing 1000 sites and performed the calculations to obtain divergence times on 10 of these windows randomly selected. For this analysis, we did not include windows from the *G03* locus between *HMGA2* and *WIF1, LEMD3, MSRB3* (i.e. Chr1A:33.28-33.38 Mb).

### Admixture mapping analysis

We used GEMMA^74^ to perform admixture mapping analysis to search for loci associated with phenotypic differences among *parvulus* (*n* = 25), *psittacula* (without western *psittacula*, *n* = 13), and the pointed-beak tree finches (*pallidus* and *heliobates* together, *n* = 19). For each pairwise comparison, we coded phenotypes as 0-1. We accounted for sample relatedness by first calculating a relatedness matrix using the GEMMA flag -*gk* 1. The relatedness matrix was then used as a covariant in linear mixed-effect models (*-lmm* 4 flag). We corrected Wald *P-*values with Bonferroni correction and significance threshold was calculated assuming *α* = 0.05.

### Phylogenetic reconstruction and tree topology weighting

We also built a whole-genome and Z-linked maximum likelihood phylogeny. We filtered our dataset to remove loci previously known to affect beak size^9^ and used *bcftools +prune* to remove sites in linkage disequilibrium (r2 > 0.1) within 100 kb windows. This final VCF was then converted to fasta format using *vcf2phylip*^75^ and used for maximum likelihood tree reconstruction in *iqtree2*^76^, with the best substitution model (i.e. PMB + F + I + G4 for both trees) being selected based on the lowest Bayesian Information Criterion. The two trees were built using 528,344 informative sites for the whole-genome phylogeny and 9152 sites for that on chromosome Z. Both phylogenies were rooted on the *Loxigilla* outgroups. We further built a phylogenetic network using the Neighbor net method in SplitsTree v.6.4.13^77–79^ based on a genetic distance matrix^80^ of 77 *Camarhynchus* individuals, as well as a *G03* phylogenetic network focusing on Darwin’s finches with either large or small beaks. For the latter, we used the above-mentioned VCF file with 100 individuals, for which tailored filtering was applied (see previous section).

To obtain support for a history of gene flow within the *G03* locus between western and northern *psittacula*, we extracted the entire *G03* region (Chr1A:33.05-33.55 Mb) and used the 4602 remaining variant sites to infer a phylogenetic network among *C. parvulus*, western, central and northern *psittacula* by first using the same subset *G03* dataset, and then building another network based on just autosomes after removal of *G03*. We also used tree topology weighting to compare local genealogies across *G03* to corroborate the evidence that western *psittacula* forms a species tree within *G03*, but is closely related with *C. parvulus* outside this locus. We therefore limited this analysis to *C. parvulus*, western *psittacula* and northern *psittacula*, using the *Loxigilla* sp. as outgroups. By using *phyml* v3.3.2, we generated a sequence of neighbor-joining trees in non-overlapping 50 SNP windows and calculated topology weighting using *twisst* v2.0^81^.

### Allele sharing analysis

The software Dsuite Dtrios v.05^82^ was used to test for introgression genome-wide among *pallidus* Santa Cruz, western *pallidus* and *C. heliobates* (BBAA = 303,796, ABBA = 227,710, BABA = 173,661, Table 1). Furthermore, we also used the Dinvestigate function within the Dsuite toolset to investigate local patterns of allele sharing by inferring the distance fraction (*df*). This statistics accounts for *dxy* for each comparison within the trio, thus providing better discrimination between introgression and incomplete lineage sorting^38^. We conducted a *df* scan in 200 SNPs windows sliding by 100 across all chromosomes, a test designed to detect patterns of allele sharing at the *G03* locus between northern and western *psittacula*: ((western *psittacula, C. parvulus*), northern *psittacula*) = ((P1, P2) P3). The distance fraction is a measure bounded between 1 and −1, with positive values reflecting gene flow between P3 and P2, whereas negative values show evidence of gene flow between P3 and P1.

### Analysis of museum morphological data

We used morphological measurements from five *Camarhynchus* species taken by P. R. G. and Robert L. Curry obtained from the California Academy of Sciences, Chicago Field Museum, Liverpool Museum of Natural History, British Museum of Natural History, American Museum of Natural History, Leland Stanford Junior University, Stockholm Museum of Natural History, Mensch und Natur Museum in Munich, Harvard Museum of Natural History and the National Museum of Natural History in Paris. We carefully inspected this dataset to remove measurements from individuals with missing information (*n* = 3), that were mislabelled or incomplete (e.g. broken body parts, *n* = 19) and that did not have a complete set of measurements (*n* = 3). This process resulted in a total of 348 individuals (19 *C. heliobates*, 65 *C. pallidus*, 145 *C. parvulus*, 20 *C. pauper*, 137 *C. psittacula*) that were retained for further analyses. We used biometric measurements from these specimens to perform a principal component analysis on their beak using the length and width of the upper mandible and the combined depth of both upper and lower mandible (Fig. 1b). A separate principal component analysis was performed on tarsus and wing length (two proxies for body size) (Fig. S1).

We then built cladograms based on our morphological data (Fig. 3a). First, we generated one Euclidean matrix using the three beak measurements (length, width and depth) and another using the three beak measurements plus wing and tarsus length (two proxies for body size) and used these matrices for neighbor-joining tree estimation using *ape*. The resulting trees, one for the beak measurements and another for beak and body size together, were compared with tanglegrams using *phytools*. Finally, we explored how many distinct morphological groups of tree finches exist. We used the R package *clustvarsel*^83^ to reduce the dimensionality of the data by selecting the set of principal components most useful for group discrimination in normal mixture models (NMMs)^84^, without a priori information about the taxonomic origin of the measurements^85–87^. We performed two analyses, one focused on all morphological measurements (i.e. wing length, tail length, length of the upper mandible, beak depth and width of the upper mandible) and another using only the three beak measurements. The number of principal components to be fed into the clustering algorithm were selected by implementing both a forward and backward stepwise algorithm to ensure robust variable selection. We then used the R package *mclust*^88^ to fit a range of NMMs, which were selected based on the lowest Bayesian Information Criterion^89^. The first analysis selected four out of five principal components to discriminate morphological groups based on their discriminatory power, these are PC1 to PC3 and PC5, whereas the NMM model based on beak measurements used all three principal components. Based on the best fit model, selected according to the lowest Bayesian Information Criterion (BIC), nine morphological groups were defined when using all morphological measurements (BIC = −2325.8), whereas eight morphological groups were defined based on beak morphology (BIC = −1628.2).

### Reporting summary

Further information on research design is available in the Nature Portfolio Reporting Summary linked to this article.

## Supplementary information

Supplementary Information

Transparent Peer Review file

Reporting Summary

## Data Availability

The data associated with this study have been deposited on Figshare and are available at the following link: 10.6084/m9.figshare.30284983. Sequence data in this study have been deposited in the Sequence Read Archive under BioProject PRJNA897926, BioProject PRJNA743742 and GenBank PRJNA678752.

## Code availability

All code used to analyze genotype data is available on Github at https://github.com/MatteoSebastianelli/Tree_finches.
