# Supplementary material for: A supergene and genomic mosaicism are associated with phenotypic evolution in Galápagos tree finches
Source: Nat Commun. 2026 Oct 11;17:10656. doi: 10.1038/s41467-026-78507-8 (PMC13655052; doi:10.1038/s41467-026-78507-8)
Supplement: Supplementary file 1 [file 41467_2026_78507_MOESM1_ESM.pdf]

Supplementary Material

Title: A supergene and genomic mosaicism are associated with phenotypic evolution in Galápagos tree finches

Matteo Sebastianelli Arbelaez[1,2], Erik D. Enbody[3], Carl-Johan Rubin[1,2], Carlos Valle[4], Lukas F. Keller[5], B. Rosemary Grant[6], Peter R. Grant[6], Leif Andersson[1,2,7]

[1]Department of Medical Biochemistry and Microbiology, Uppsala University, Uppsala, Sweden.

[2]SciLifeLab, Uppsala University, Uppsala, Sweden

[3]Department of Computational Biology, Cornell University, NY, USA.

[4]Colegio de Ciencias Biológicas y Ambientales, Galápagos Science Center GSC, Universidad San Francisco de Quito USFQ, Quito, Ecuador.

[5]Department of Evolutionary Biology and Environmental Studies, University of Zürich, Zürich, Switzerland

[6]Department of Ecology and Evolutionary Biology, Princeton University, Princeton, NJ, USA.

[7]Department of Veterinary Integrative Biosciences, Texas A&M University, College Station, TX, USA.

Supplementary Note 1

Test for introgression within the C. pallidus/C. heliobates group

We compared western *pallidus*, *pallidus* S. Cruz and *C. heliobates* genome-wide, using the two *Loxigilla* species as outgroup. Central *pallidus* groups with the western subspecies in the most abundant tree topology genome-wide (Fig. S4, BBAA), followed by an ABBA tree that reflects gene flow between *C. heliobates* and western *pallidus* (Table 2). Gene flow with the latter is the likely cause for the preponderance of ABBA over BABA trees, which is otherwise unlikely to arise because of incomplete lineage sorting, which would result in comparable occurrences of ABBA and BABA trees.

Supplementary Note 2

Genomic landscape of absolute divergence

We explored genome-wide patterns on genomic differentiation by estimating *dxy* in 10 kb non-overlapping windows for all *Camarhynchus* taxa. We observed regions of relatively high *dxy,* including one on chromosome 1A when comparing large tree finches with small tree finches (Fig. S6), highlighting the *G03* locus segregating for ancestral haplotypes with major effects on beak and body size[1,2] (*Supplementary Notes 3* and *4*). The other *dxy* divergence peaks on other chromosomes are shared in all the comparisons among taxa (also highlighting ancestral variation), but are not present in any *Fst* contrast. For instance, a *dxy* divergence peak on chromosome 11 spanning a 380 kb region on chromosome 11 points towards a polymorphism that is shared in all *Camarhynchus* populations. Interestingly, based on our dataset, haplotypes within this region are either homozygous to *C. parvulus* or heterozygous, but never homozygous for the alternative allele (Fig. S7a). Furthermore, this pattern is shared with the *Loxigilla* outgroups as well, suggesting this polymorphism is old. Indeed, by leveraging our entire genomic dataset consisting of 415 individual Darwin's finches (including the 77 considered in the current study), we confirm this polymorphic region is shared by all Darwin's finch lineages (Fig. S7a), and likely most of the members of the Thraupidae family. By calculating nucleotide diversity in a central *psittacula* individual (106Saz3, heterozygous at the 380 kb region), we observed a sudden increase of nucleotide diversity to > 3% within this region, compared to a genome-wide background of 0.1% (Fig. S7b). However, a more in-depth investigation revealed this to be a sex-dependent pattern, with all individuals classified as heterozygous based on this region being females. By normalizing the sequencing depth to the median per site across this region, we reveal that a copy number variation is present in female individuals (Fig. S7c), but not in males (Fig. S7d), and likely involves a translocation of a copy of this~ 380 kb region of chromosome 11 on the W chromosome, given that females are the heterogametic sex in birds. Our results imply that the translocation happened during the evolution of tanagers well before the evolution of Darwin's finches.

Supplementary Note 3

Gene content at divergence islands of relative differentiation (*Fst*)

By focusing on regions of elevated *Fst*, we identified four major divergent regions differentiating tree finch populations: these are located on chromosomes 1A, 5, 6 and Z. The divergence island on chromosome 1A hits a key locus for Darwin's finch evolution (*i.e. G03*), known to mediate ecological adaptation of Darwin's finches by affecting beak size[3]. Specifically, the divergence peak revealed by this study maps on a ~500 kb core region that includes four genes: the *WNT inhibitory factor 1* (*WIF1*), the *LEM domain-containing protein 3* (LEMD3), the *methionine sulfoxide reductase B3* (*MSRB3*) and the *high mobility AT-hook 2* (*HMGA2*). The latter gene is a chromatin-associated protein that can affect the transcription factor activity[4] and has been associated with dwarfism in mice[5] and human height[6,7].The presence of this locus therefore explains the occurrence of the divergence peak in every contrast involving subspecies with known difference in beak size (Fig. 5 and S8).Even though sample size was too small to perform and *Fst* contrasts among *C. pallidus* subspecies and *C. heliobates,* haplotypes at this locus (Fig. 5, bottom panel) suggest that these two species also share haplotypes similar to the large tree finch *C. psittacula*. In fact, both *C. pallidus* and *C. heliobates* (to a lesser extent) overlap with *C. psittacula* in beak size (*i.e.* PC1), but not beak shape (*i.e.* PC2, Fig. 1c).

Chromosome 5 harbours another divergence peak when comparing northern *psittacula* with each of the other closely related tree finches (western *psittacula*, *C. pauper* and *C. parvulus*), a region spanning approximately 600 kb that encloses 13 genes (Fig. S9; Table S3). However, most fixed differences between northern *psittacula* and the other finches accumulate around the *INO80 complex ATPase subunit* (*INO80*), a gene that plays a pivotal role in chromatin remodelling [8], smooth muscle differentiation[9] but that is also involved in the response to DNA replication stress due to heat exposure by affecting nucleosome mobility[10].

An 80 kb region on chromosome 6 that includes six genes (Table S3, Fig. S10) distinguishes the medium tree finch *C. pauper* from northern *psittacula* and, to some extent, western *psittacula*, specifically in correspondence of a region harbouring *ATP binding cassette subfamily G2* (*ABCG2*), *biogenesis of lysosomal organelles complex 1 subunit 2* (*BLOC1S2*) and *cytochrome P450 family 2 subfamily C member 23* (*CYP2C23*), whereas a narrower divergent region between western and northern *psittacula* includes the *arachidonate 5-lipoxigenase* (*ALOX5*). Strikingly, most of the above mentioned genes (*i.e. ALOX5* and *CYP2C23* together with *SCD* and *PDK2L1*) are all highly expressed in the liver and play a crucial role in fatty acid metabolism [11–13], whereas *ABCG2* is known to affect egg shell coloration [14], with the *stearoyl-CoA desaturase* (*SDC*), *polycystin 2 like 1, transient receptor potential cation channel* (*PDK2L1*) and *BLOC1S2* being also involved in egg production[15]. However, in his book Lack describes Darwin's finch eggs to "*look much alike, being white with small pinkish spots*", even though they differ in size to some extent[16].

The divergence landscape is different on chromosome Z, with chromosome-wide elevated delta allele frequency in most comparisons (Fig. S11). However, by using the contrast between the

least divergent tree finches western *psittacula* and *C. parvulus*, we narrowed down the number of divergent regions to four, based on variant sites with delta allele frequency > 0.8 (Fig. S11, dashed line). The first divergence peak on chromosome Z spans approximately 86 kb and includes the *lysyl-oxidase* (*LOX*) and the *serum response factor binding protein 1* (*SREBP1)*, with the SNP with the highest delta allele frequency between western *psittacula* and *C. parvulus* located between the two genes. *LOX* is known to affect multiple processes, including bone matrix formation and osteogenic differentiation[17], whereas *SRFBP1* is involved in regulation of concentration of cellular NAD/NADH[18]. A second ~430 kb region includes two genes: the *ADAM metallopeptidase with thrombospondin type 1 motif 19* (*ADAMTS19*), known to play a key role in several cellular processes but also during development and ovulation[19], and *chondroitin sulfate synthase 3 CHSY3*, highly expressed in cartilages [20] and essential for bone and joint development[21]. A 60 kb region at approximately 37 Mb has a predominance of genes involved in egg production and embryonic maturation (*i.e. TUT7*, *GOLM1, NTRK2*) in birds and mammals[22–24] as well as egg shell color (*i.e. ISCA1* and *AGTPBP1*) [25]). Finally, the most divergent peak between western *psittacula* and *C. parvulus* (with SNPs completely fixed between the two) maps into a 120 kb region at 57 Mb that includes only one gene: the *follistatin* (*FST*), a gene widely known for its pleiotropic effects affecting several important biological functions in birds such growth rates[26] and fertility in chicken[27] as well as head pigmentation in Gouldian finches [28,29]. It is worth noting that the sites that reach complete fixation between western *psittacula* and *C. parvulus* are located upstream *FST*, suggesting their involvement in the regulation of its expression.

Supplementary Note 4

Gene content associated with admixture mapping divergence peaks

We performed pairwise admixture mapping scans between tree finch lineages that are most phenotypically divergent: the small and large blunt beak tree finches (*parvulus* and *psittacula,* excluding western *psittacula*) and the pointed beak tree finches (Table S4). Some regions of association mirror those highlighted in the *Fst* analysis (*Supplementary Note 3*), such as a divergence peak at *G03* (chromosome 1A) resulting from the comparison between small and large birds (*parvulus* with either *psittacula* and the pointed beak tree finches) and a divergent peak on chromosome 5 between *parvulus* and *psittacula*. Besides these two loci, other divergent SNPs between *parvulus* and *psittacula* are found on chromosome 2 (Fig. S14a). Two map on the *aryl hydrocarbon receptor* (*AHR*) gene (one missense variant Thr382Met and one intronic variant), a gene known to be involved in response to toxic agents, inflammation, autoimmune disorders and regulation of the circadian rhythm in humans[30,31]. Three other significant SNPs hit intronic sequences of the *calcium voltage-gated channel auxiliary subunit beta 2* (*CACNB2*), a gene that has been associated with vocal learning in zebra finches (*Taenopygia guttata*)[32], eggshell production and heat stress tolerance in chickens[33,34], whereas an additional SNP hits the intronic sequence of the *mohawk homeobox* gene (*MKX*), known

to be an important development regulator in vertebrates[35]. Chromosome 3 harbours a divergence peak that was also reported in Rubin *et al*., 2022[1], with our most significant SNP located ~40 kb downstream *tRNA methyltransferase 11 homolog* (*TRMT11*), associated with heat tolerance[36] and breast size in chicken[37]. An interesting locus is the one highlighted by the divergence peak on chromosome 7 that was also reported in previous studies[1,3]. In this divergence island, the most significant SNP is located in an intron of the *insulin-link growth factor binding protein 2* (*IGFBP2*) a gene affecting growth in chicken[38]. This gene occurs in tandem with the *insulin-line growth factor binding protein 5* (*IGFBP5*), another gene known to affect growth in chicken[39]. Finally, the last divergent region between *parvulus* and *psittacula* is found on chromosome Z, with significant SNPs located a few base pairs upstream of an uncharacterized gene.

Differences between *parvulus* and the pointed beak tree finches (besides *G03*) are found on chromosome 2 (Fig. S15a), approximatively 40 kb upstream the *mesenchyme homeobox 2* (*MEOX2*) gene known to affect growth during development[40], a region downstream the *tripartite motif containing 66* gene (*TRIM66*)[41] and a locus on chromosome 6 that includes the *zona pellucida glycoprotein 4* (*ZP4*), *CYP2C45* and *tolloid like 2* gene (*TLL2*), genes involved in the structure of the egg envelope[42], fatty acid metabolism[12] and activation of myostatin[43,44], respectively. Importantly, this locus on chromosome 6 seems to differentiate pointed beak and blunt beak tree finches. In fact, this divergence island is also evident in the comparison between *psittacula* and pointed beak tree finches (Fig. S15b), together with a locus on chromosome 2 that includes the *transforming growth factor-beta receptor type 1 protein* (*TGFBR1*) gene, known to affect growth in chicken[45], and *collagen type XV alpha 1 chain (COL15A1)* gene[46].

Figures

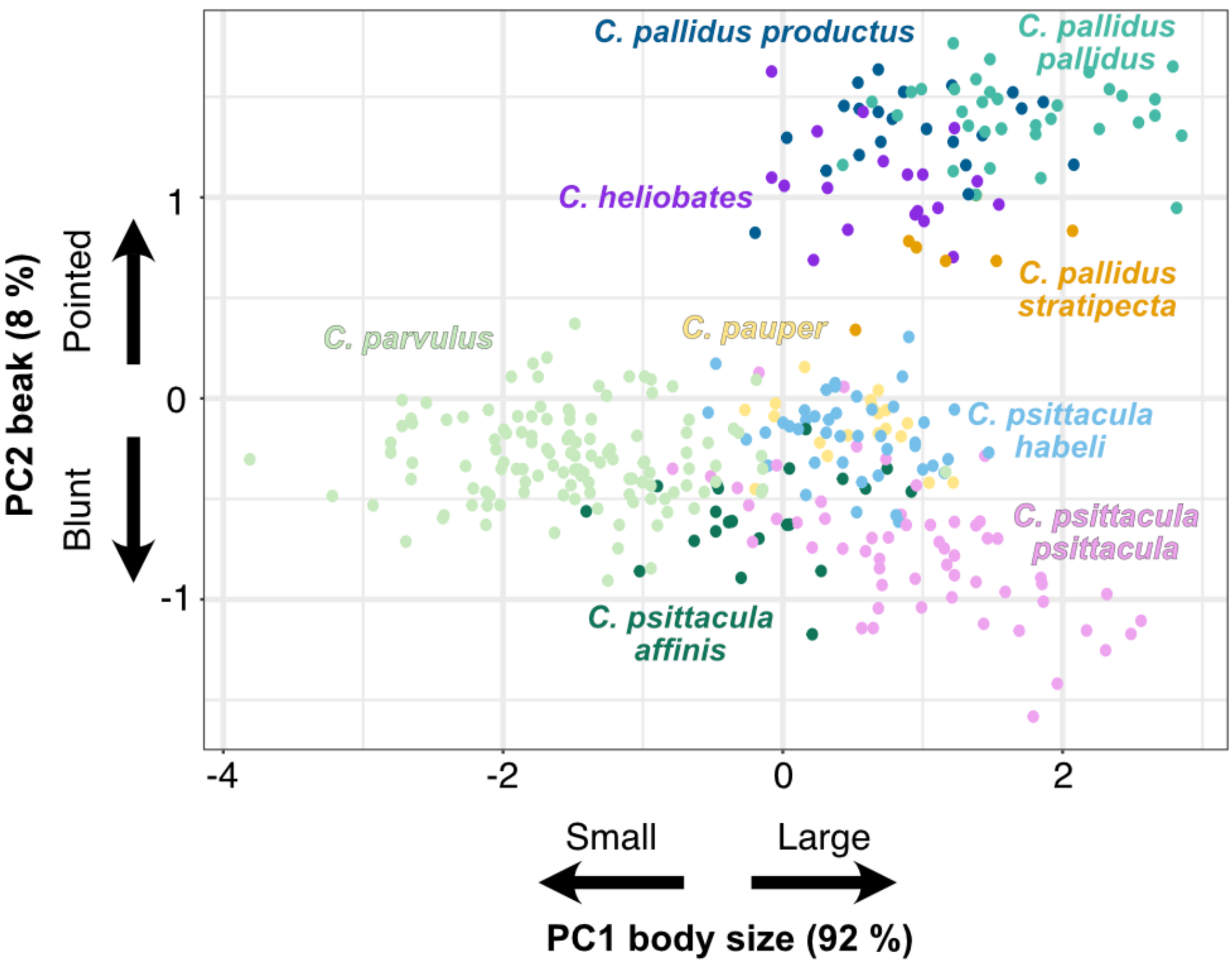

Fig. S1: Morphological variation in beak and body size across *Camarhynchus* taxa. PCA plot based on measurements from 348 museum specimens reflecting morphological variation in body size (x-axis) based on wing and tarsus length and beak shape (y-axis) based on beak length, depth, and width.

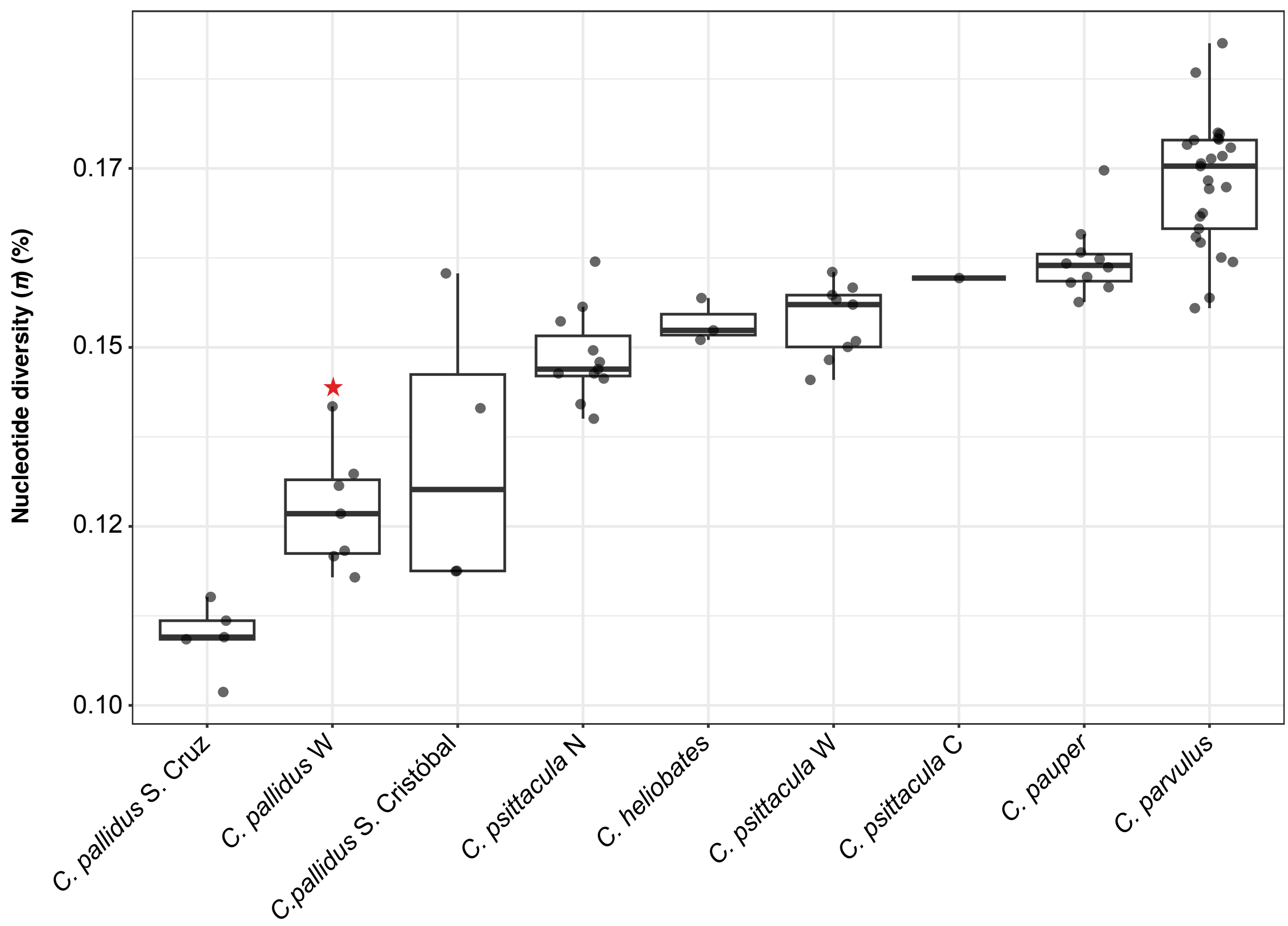

Fig. S2: Individual nucleotide diversity (π) of the critically endangered tree finches. Individual nucleotide diversity for all *Camarhynchus* individuals analysed in this study. The red star marks a putative hybrid *C. pallidus* x *C. heliobates* (102Isa1940) originally classified as *C. pallidus*, an individual with the highest nucleotide diversity of all the other *C. p. productus*. The figure also illustrates that all *C. pauper* have higher nucleotide diversity than individuals from islands of comparable size (*e.g.* the northern *psittacula* from Pinta and Marchena) and, interestingly, similar to the western *psittacula*, known to hybridize with *C. parvulus*.

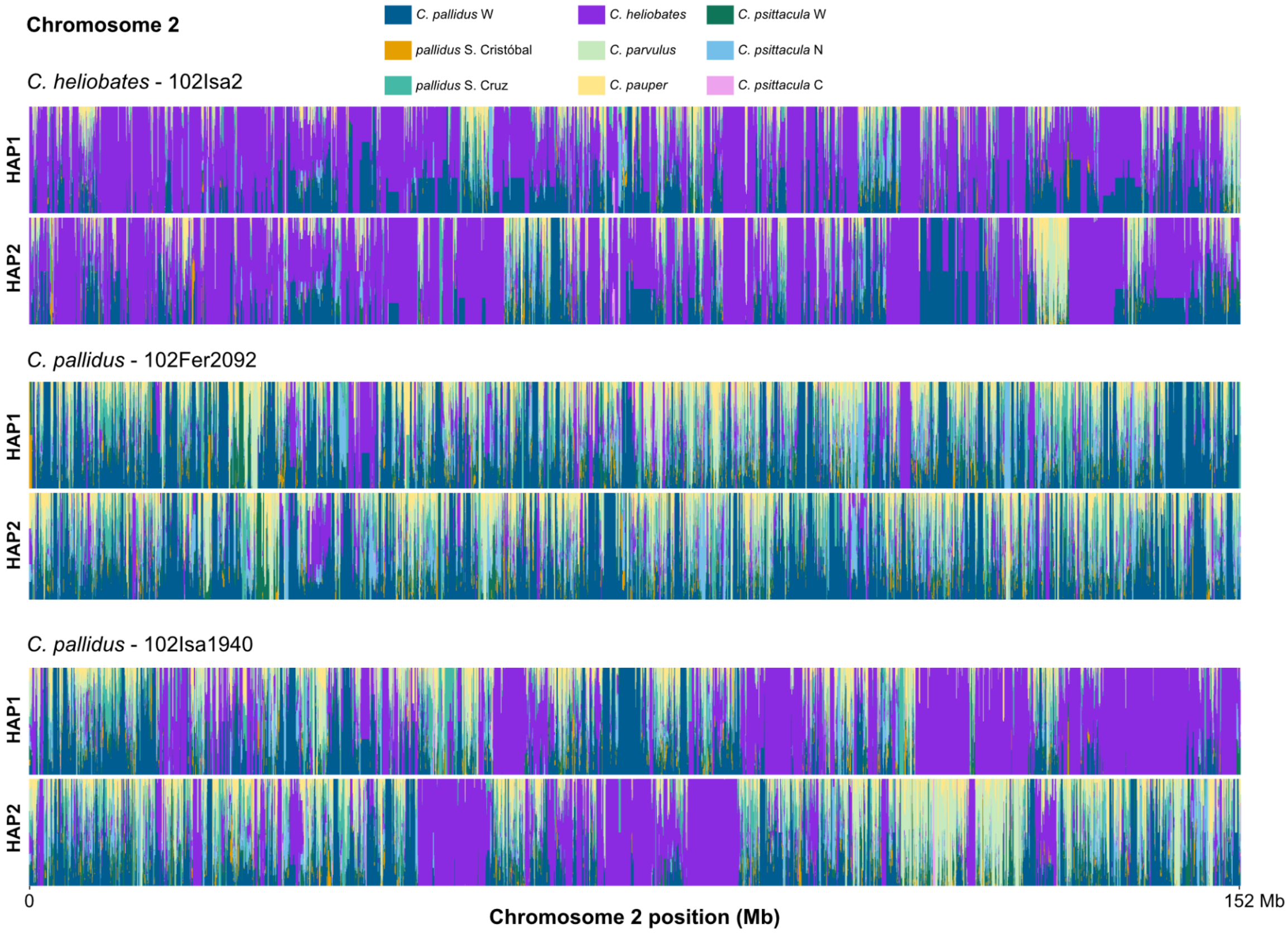

Fig. S3: Chromosome-level Genealogical Nearest Neighbour (GNN) haplotype plot. Example of a GNN haplotype plot for chromosome 2, the largest chromosome in the Darwin's finch genome, between a *C. heliobates* individual (top panel, 102Isa2), a *C. pallidus productus* from Fernandina Island (middle panel, 102Fer2092) and the putative *C. pallidus productus* x *C. heliobates* hybrid (bottom panel, 102Isa1940), the latter showing mixed ancestry tracks.

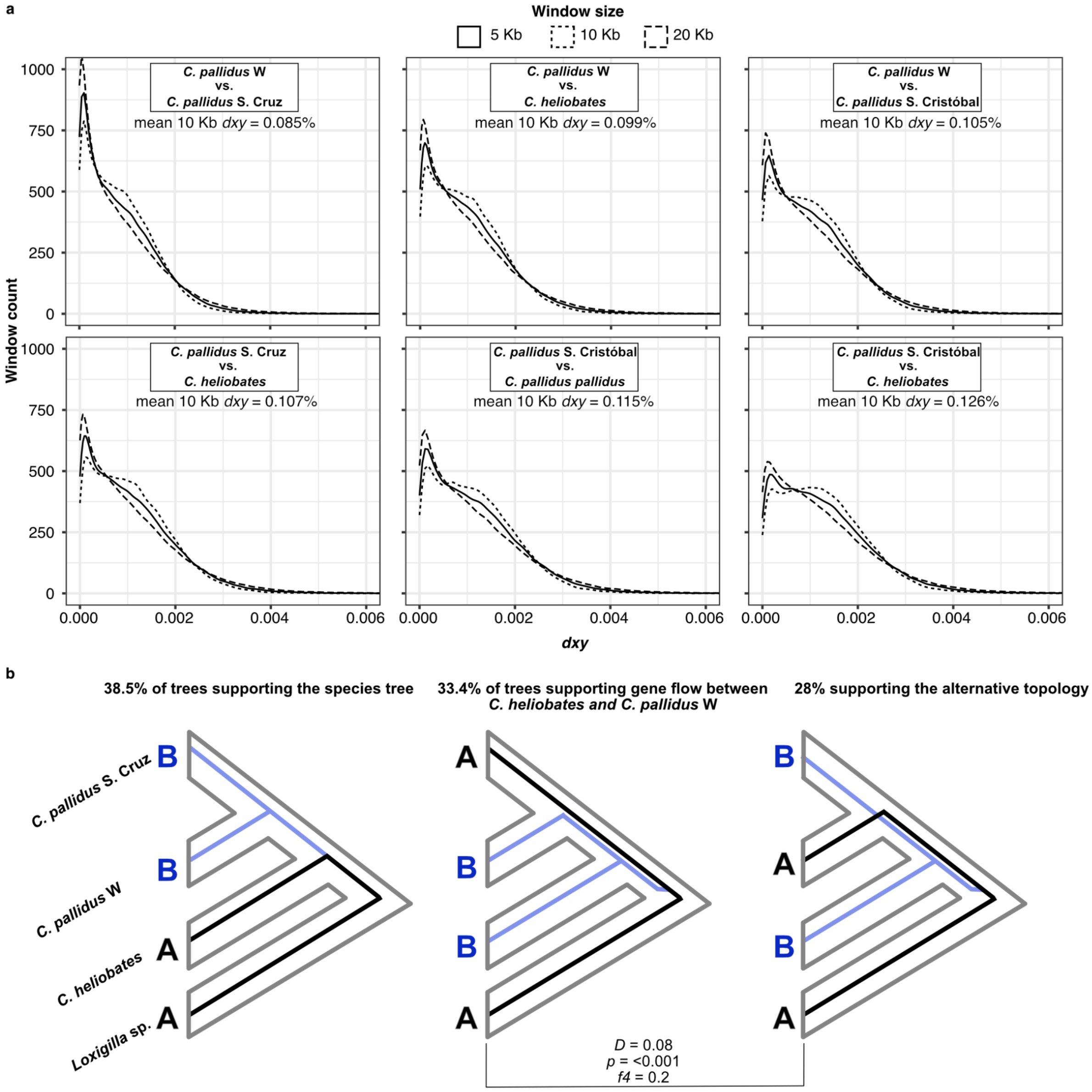

Fig. S4: Patterns of absolute diversity and introgression in the *Camarhynchus pallidus/C. heliobates* group. a) *dxy* distributions among *Camarhynchus pallidus/heliobates* populations. Panels are sorted left to right and top to button to reflect decreasing *dxy* mean values based on 10 kb windows. **b)** Results for ABBA-BABA test for introgression among *C. pallidus* S. Cruz, western *pallidus* and *C. heliobates*, with panels sorted left to right according to the most common tree topology. *Loxigilla noctis* and *L. barbadensis* are used as outgroups.

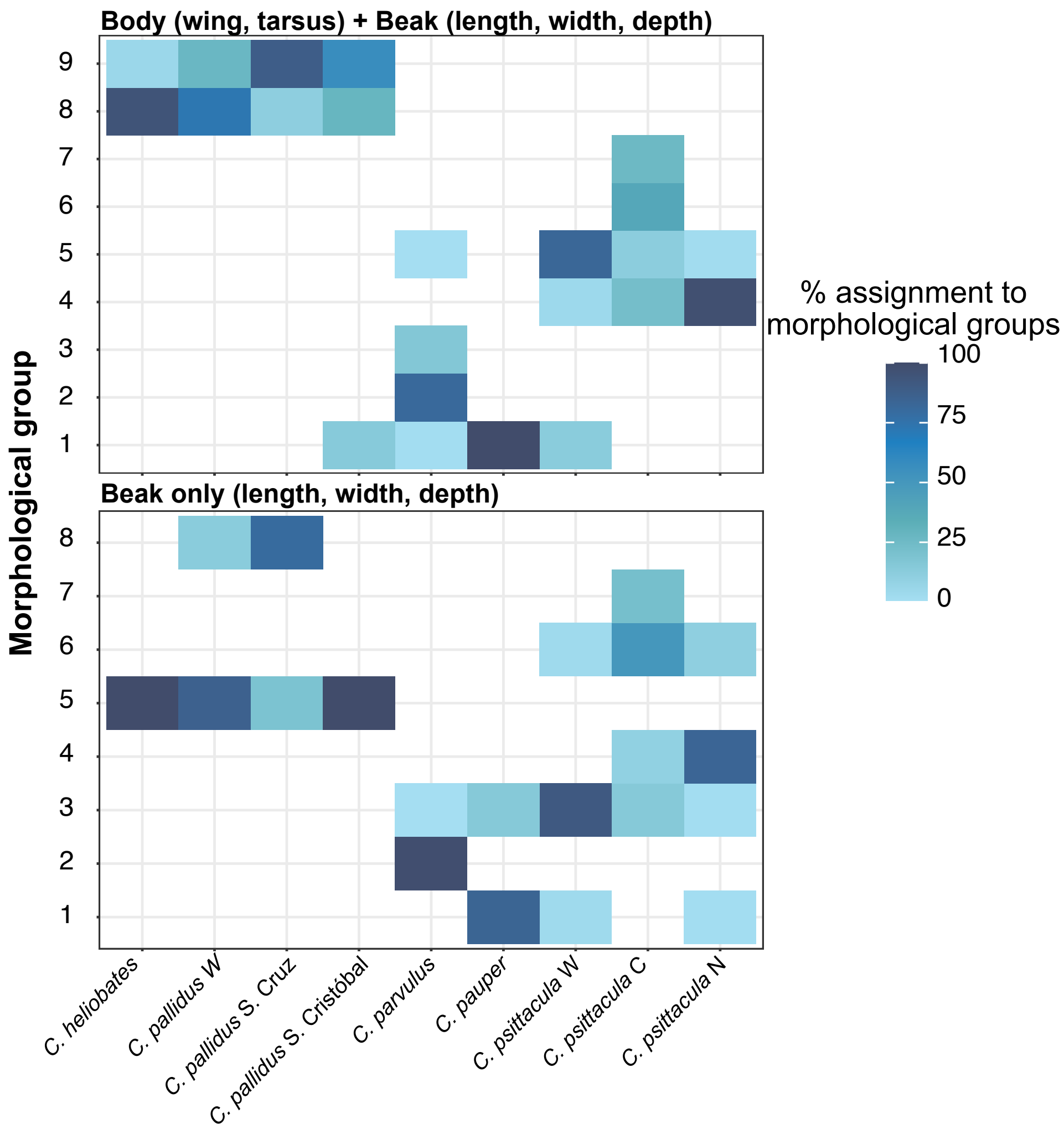

Fig. S5: Morphological space in *Camarhynchus*. Percentage of assignment of each *Camarhynchus* subspecies to the morphological groups inferred with normal mixture models (NMMs). In the upper panel, morphological groups are inferred using a combination of five morphological measurements comprising body measurements, whereas in the bottom panel, morphological groups are based on the beak measurements only.

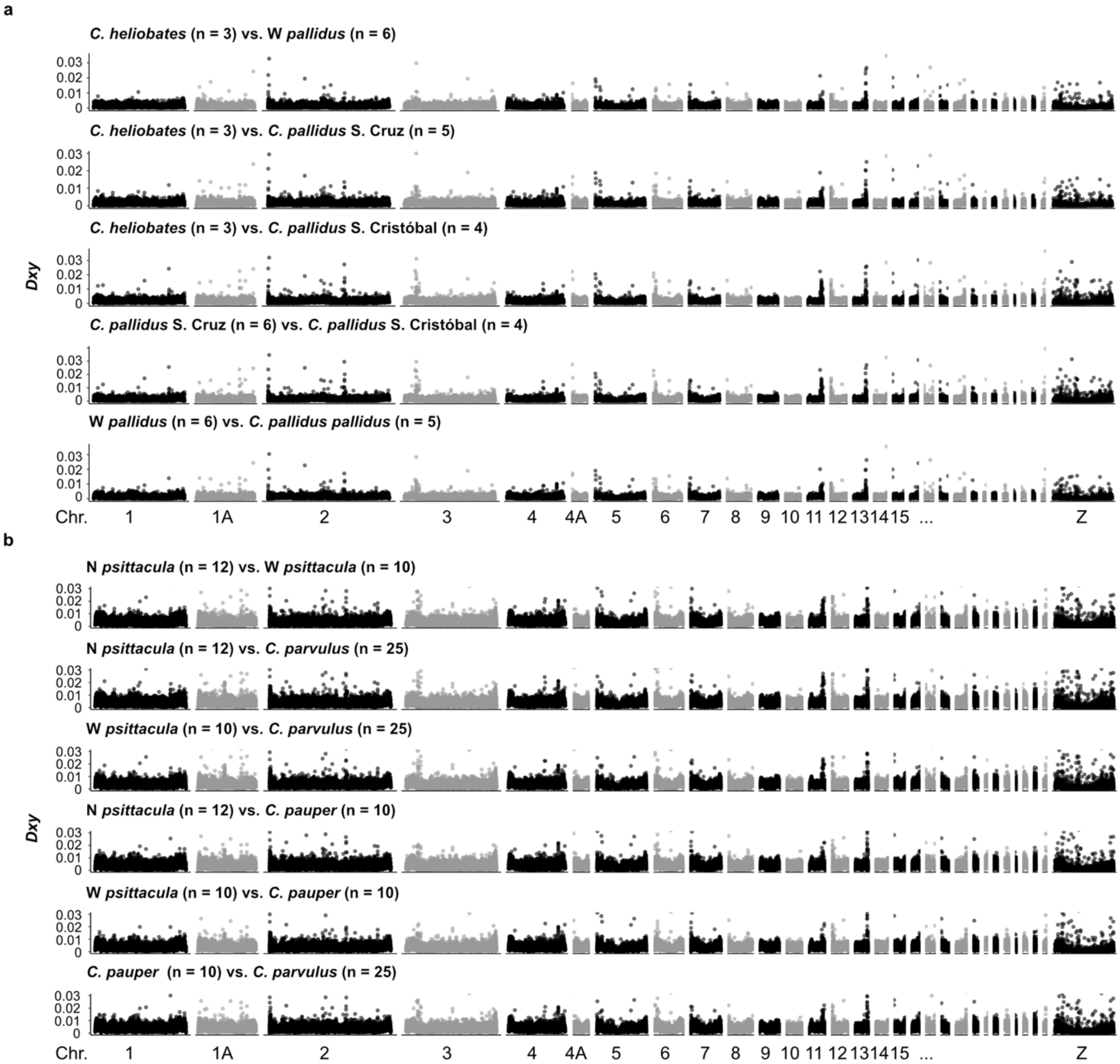

Fig. S6: Genomic landscape of absolute divergence. Genome-wide *dxy* calculated in 10 kb non-overlapping windows among a) *C. heliobates* and *C. pallidus* as well as b) among *C. parvulus*, *C. pauper* and *C. psittacula* subspecies.

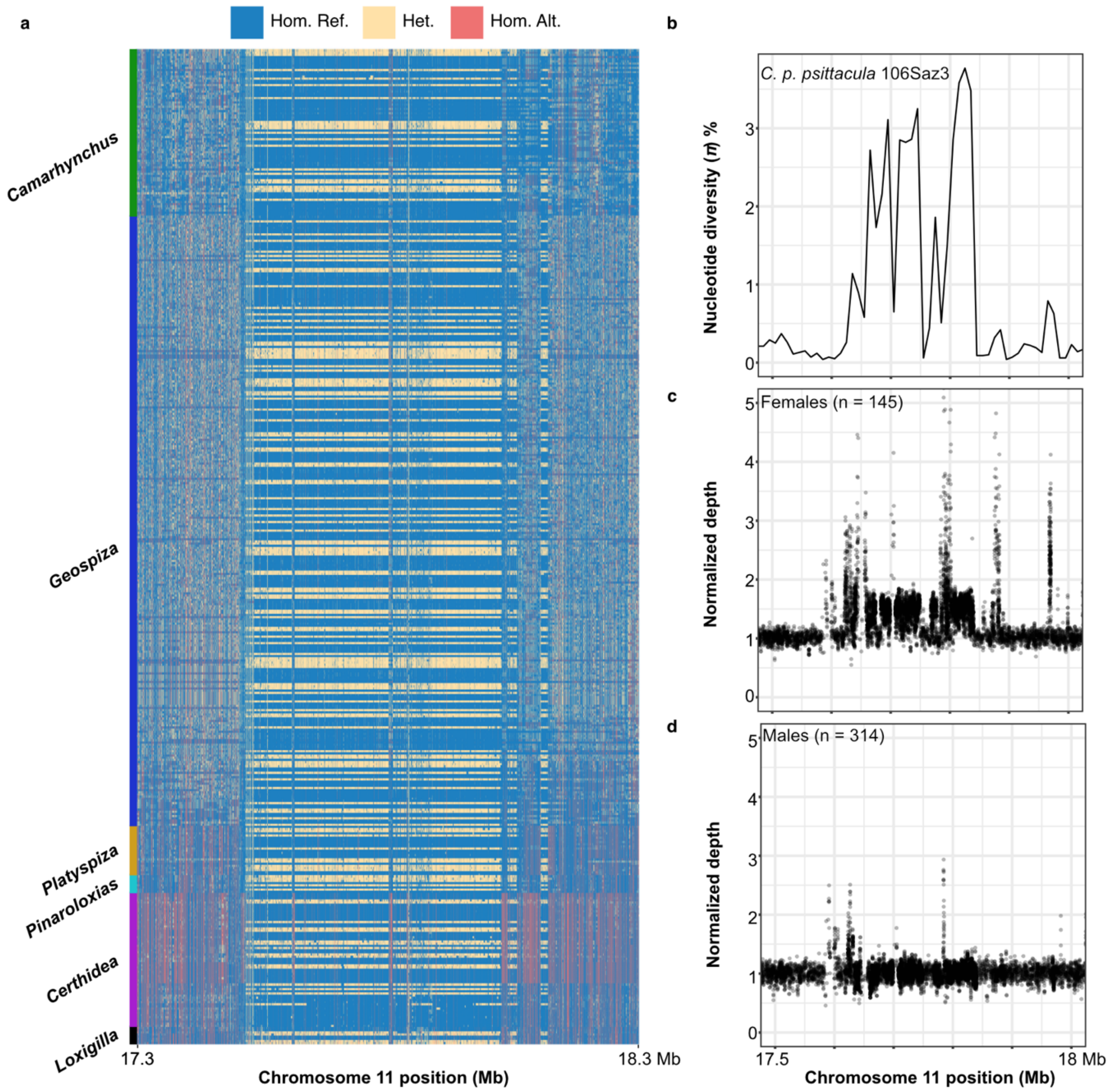

Fig. S7: Haplotypes, nucleotide diversity and copy number variation patterns on Darwin's finch chromosome 11. Individual haplotypes across the ~380 kb region on chromosome 11. Haplotypes of 415 Darwin's finch and outgroup individuals are shown in **a)**, sorted according to phylogenetic order, with the *Camarhynchus* genus (the most recent) placed at the top. **b)** shows nucleotide diversity (π) for a female central *psittacula* (106Saz3), whereas median normalized sequencing depth across this region for 145 females and 314 males is shown in **c)** and **d)**, respectively.

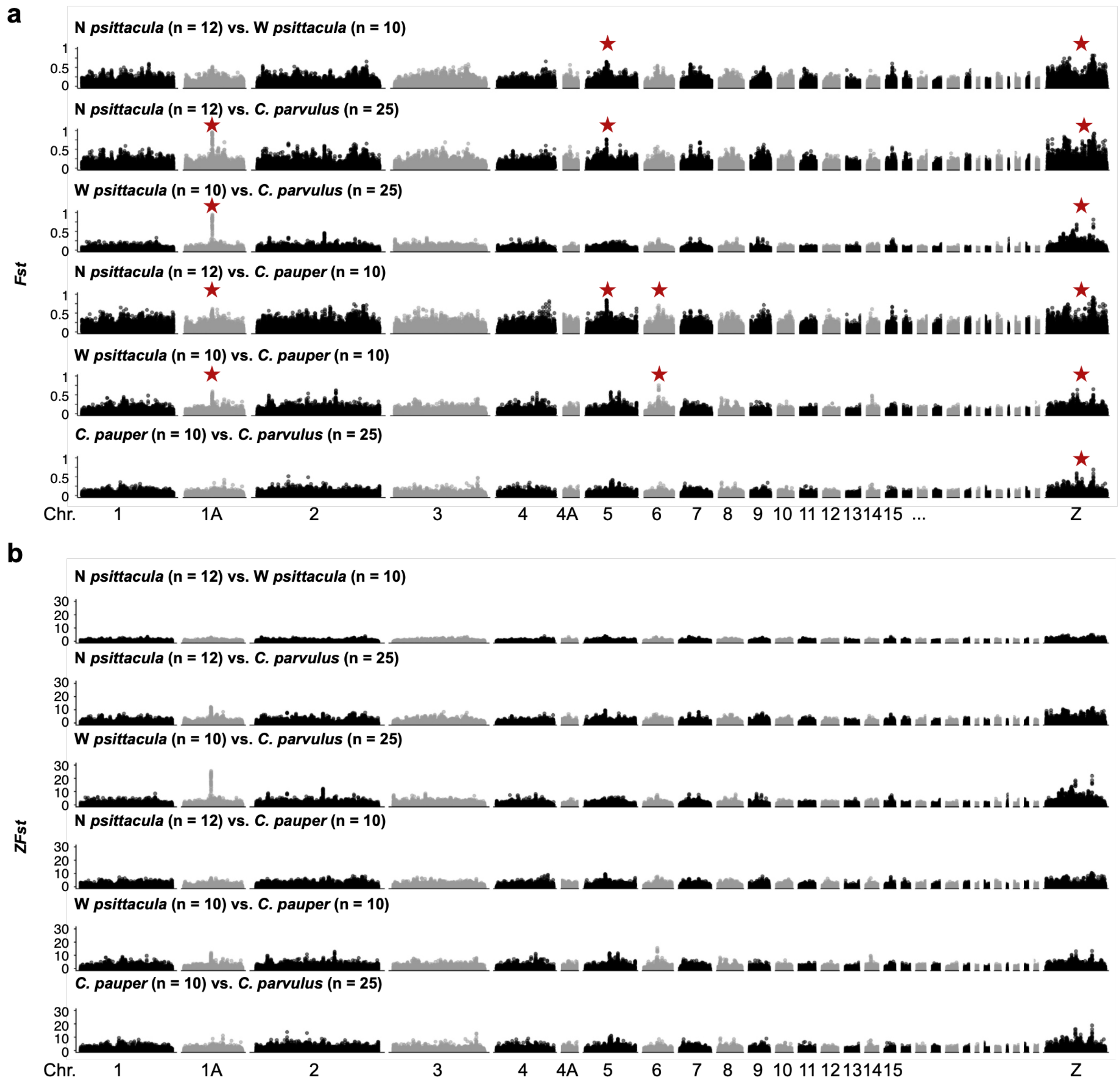

Fig. S8. Genomic landscape of relative divergence among large (*C. psittacula*), medium (*C. pauper*), and small (*C. parvulus*) tree finch taxa. a) Genome-wide raw F_{ST} calculated in 10 kb windows for each pairwise comparison. **b)** Corresponding Z-transformed *Fst* (ZF_{ST}), expressed as standard deviations from the genome-wide average; values near zero reflect background differentiation, whereas strong deviations from zero indicate outlier regions.

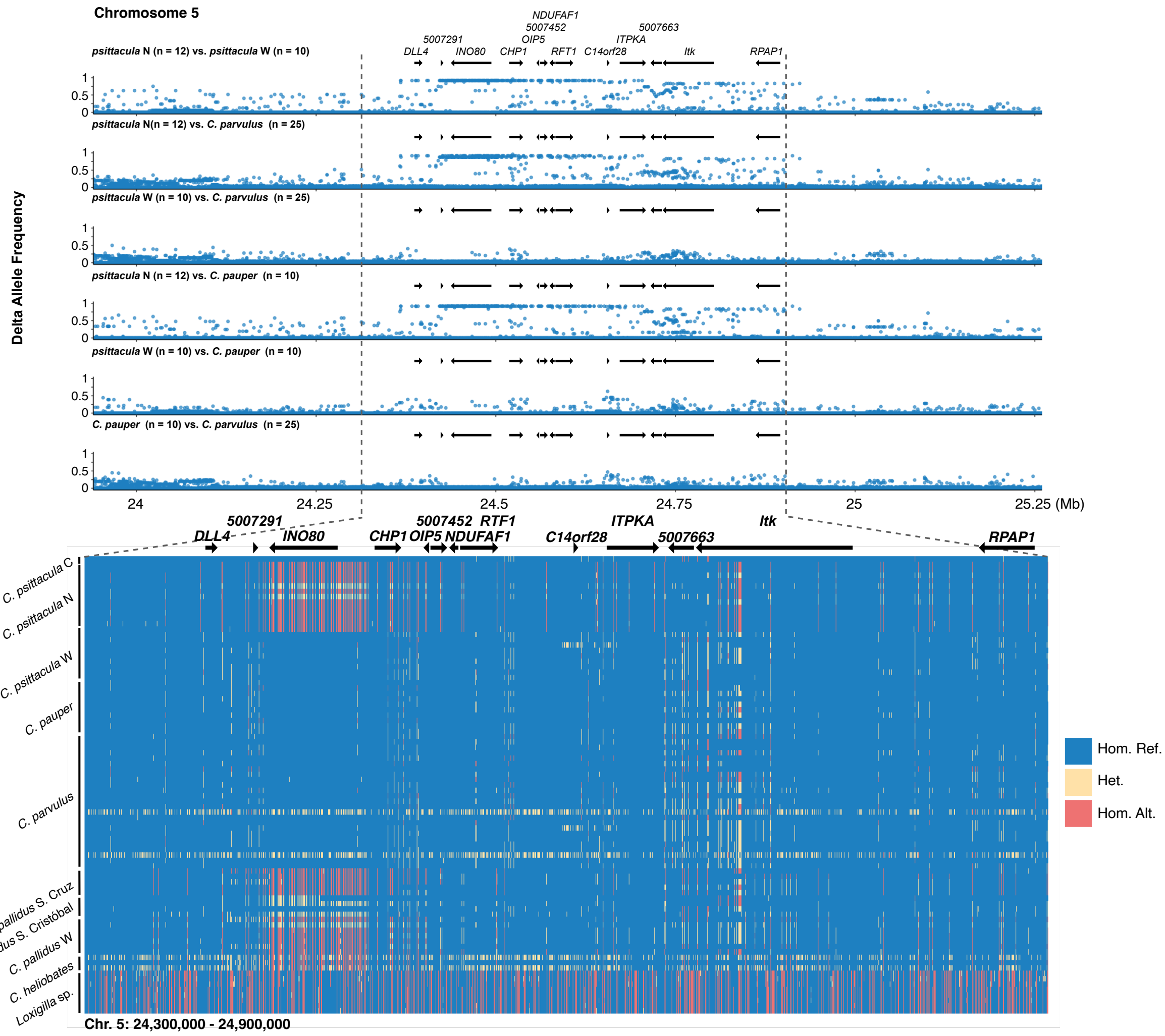

Fig. S9: Close-up of chromosome 5 divergence peak. Delta allele frequency plot among *C. psittacula*, *C. pauper* and *C. parvulus* (upper panel) as well as individual haplotypes across a 600 kb region harbouring most allele frequency differences (bottom panel). The latter includes all the taxa used in this study, including the outgroups. The blue colour in the lower panel indicates homozygosity to *C. parvulus*.

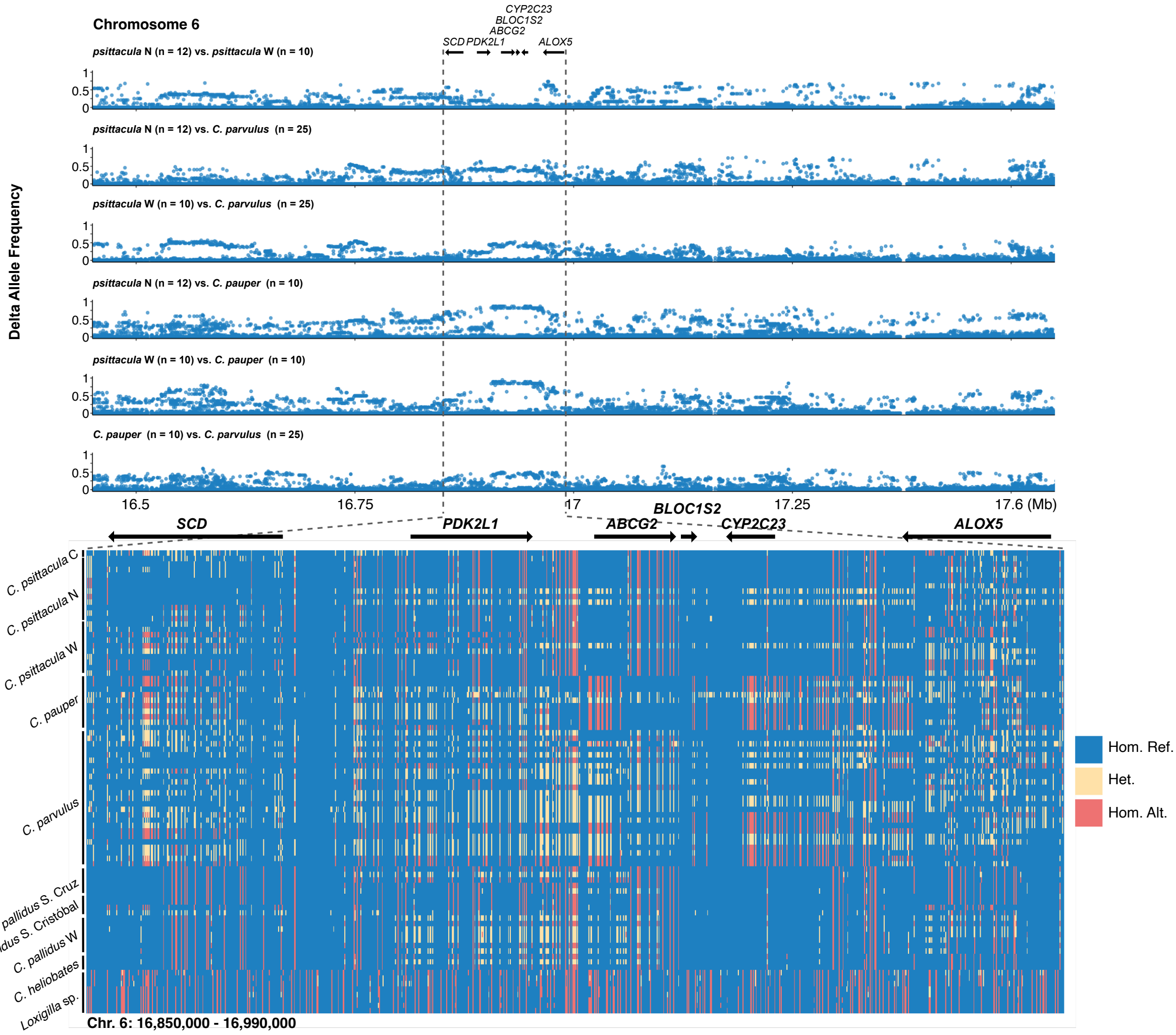

Fig. S10: Close-up of chromosome 6 divergence peak. Delta allele frequency plot among *C. psittacula*, *C. pauper* and *C. parvulus* (upper panel) as well as individual haplotypes across a 600 kb region harbouring most allele frequency differences (bottom panel). The latter includes all the taxa used in this study, including the outgroups. The blue colour in the lower panel indicates homozygosity to *C. parvulus*.

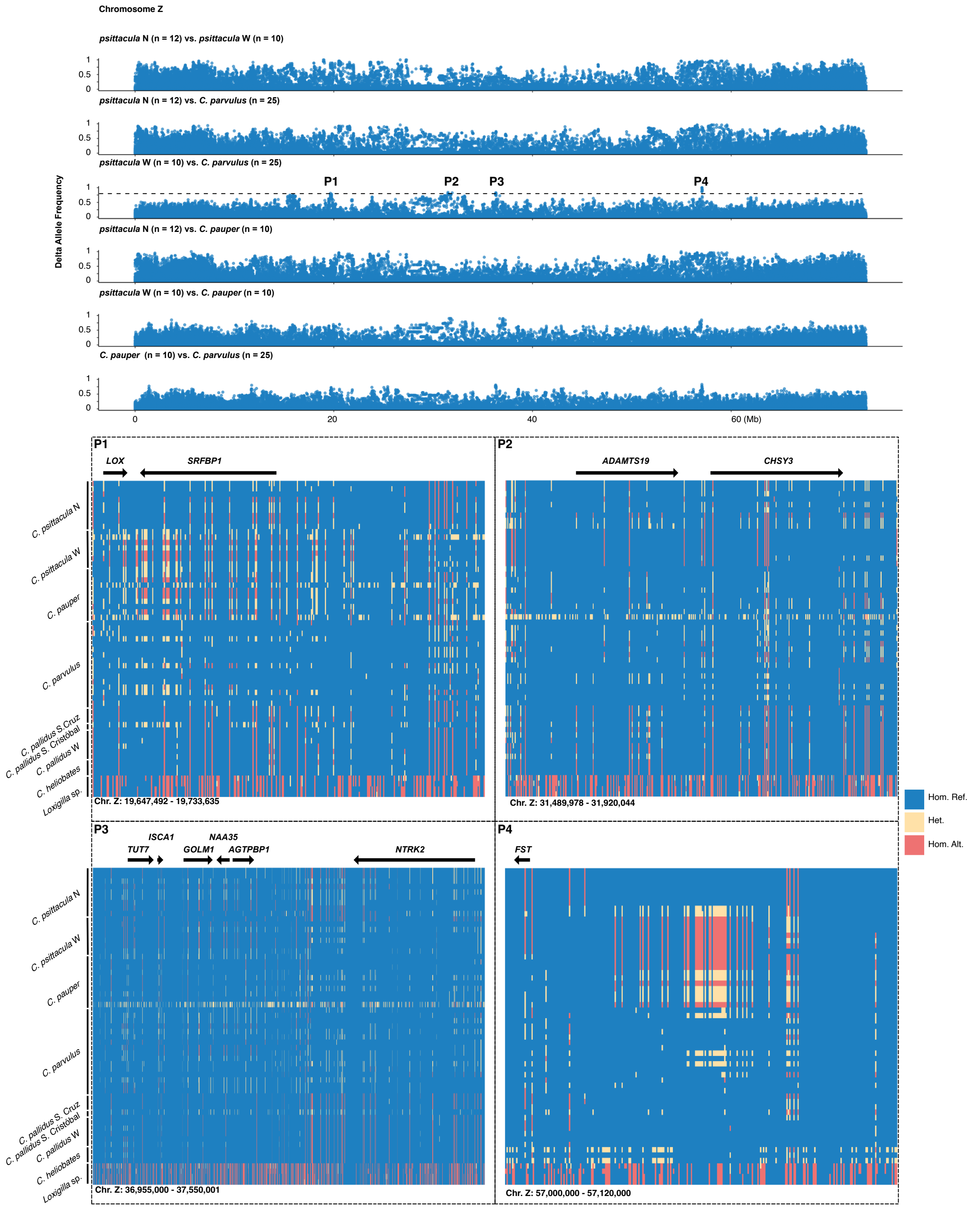

Fig. S11: Chromosome Z divergence peaks. Delta allele frequency among male *C. psittacula*, *C. pauper* and *C. parvulus* (upper panel) and close up of individual haplotypes (lower panel) within four loci selected based on delta allele frequency between western *psittacula* and *C. parvulus* > 0.8. The blue colour in the lower panel indicates homozygosity to *C. parvulus*.

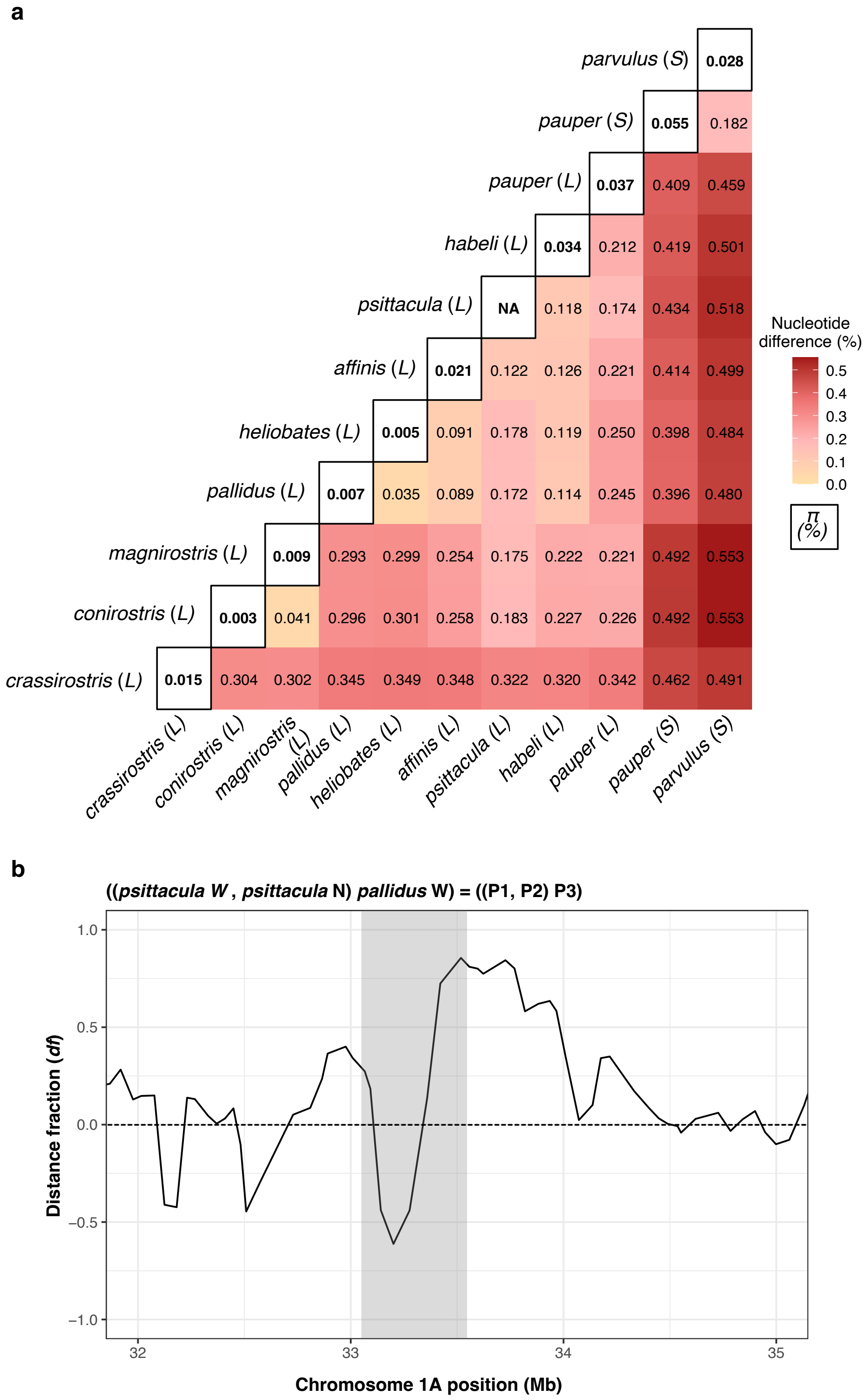

Fig. S12: Local *G03* patterns of diversity and allele sharing. a) Patterns of pairwise nucleotide divergence (*dxy,* in yellow/red shades) and nucleotide diversity (π, on the diagonal cells) calculated for the *G03* alleles of the large finches, the medium tree finch *C. pauper* (small *S* and large *L*) and the small tree finch *C. parvulus.* The relatively high π in *affinis* could reflect an evolutionary history in which both *pallidus* and *psittacula* contributed to its large *G03* haplotype, as supported by an analysis of allele sharing in **b)**, in which excess of shared alleles between western and northern *psittacula* is reflected by positive *df* values, whereas negative values reflect excess of allele sharing between *pallidus* W and western *psittacula*. Gray shading indicates the *G03 locus.*

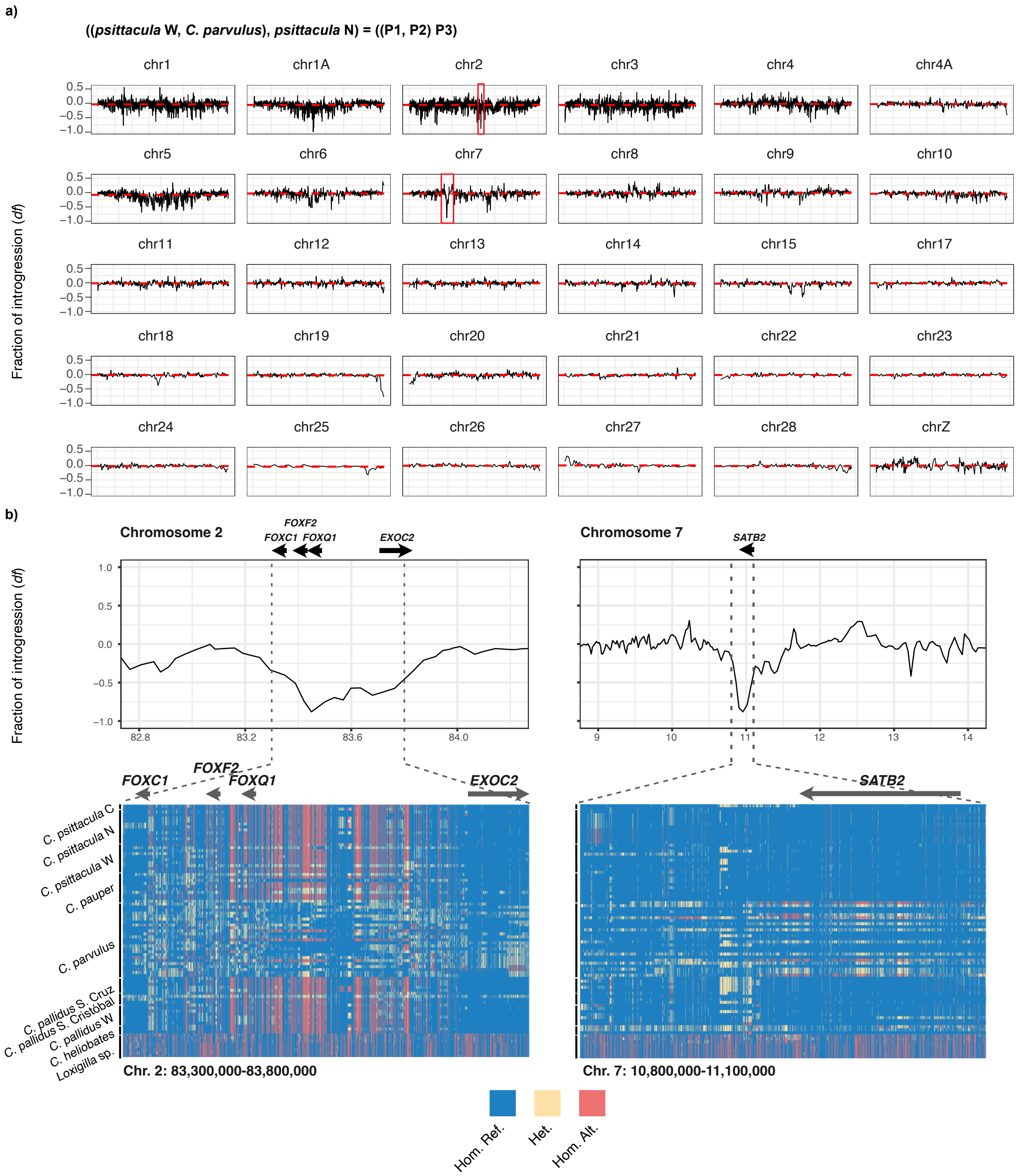

Fig. S13: Genome-wide patterns of allele sharing between northern and western *psittacula*. a) An inspection of patterns of shared alleles between northern and western *psittacula* across chromosomes reveals that, besides the *G03* locus on chromosome 1A, two additional loci have a distance fraction (*df*) exceeding 75% (red boxes), one on chromosome 2 - a locus that was associated with genetic differentiation among *Geospiza* (Rubin and Enbody *et al.* 2022) - and one on chromosome 7. A close-up of the *df* across these loci, as well as their gene content and individual haplotypes are shown in **b)**.

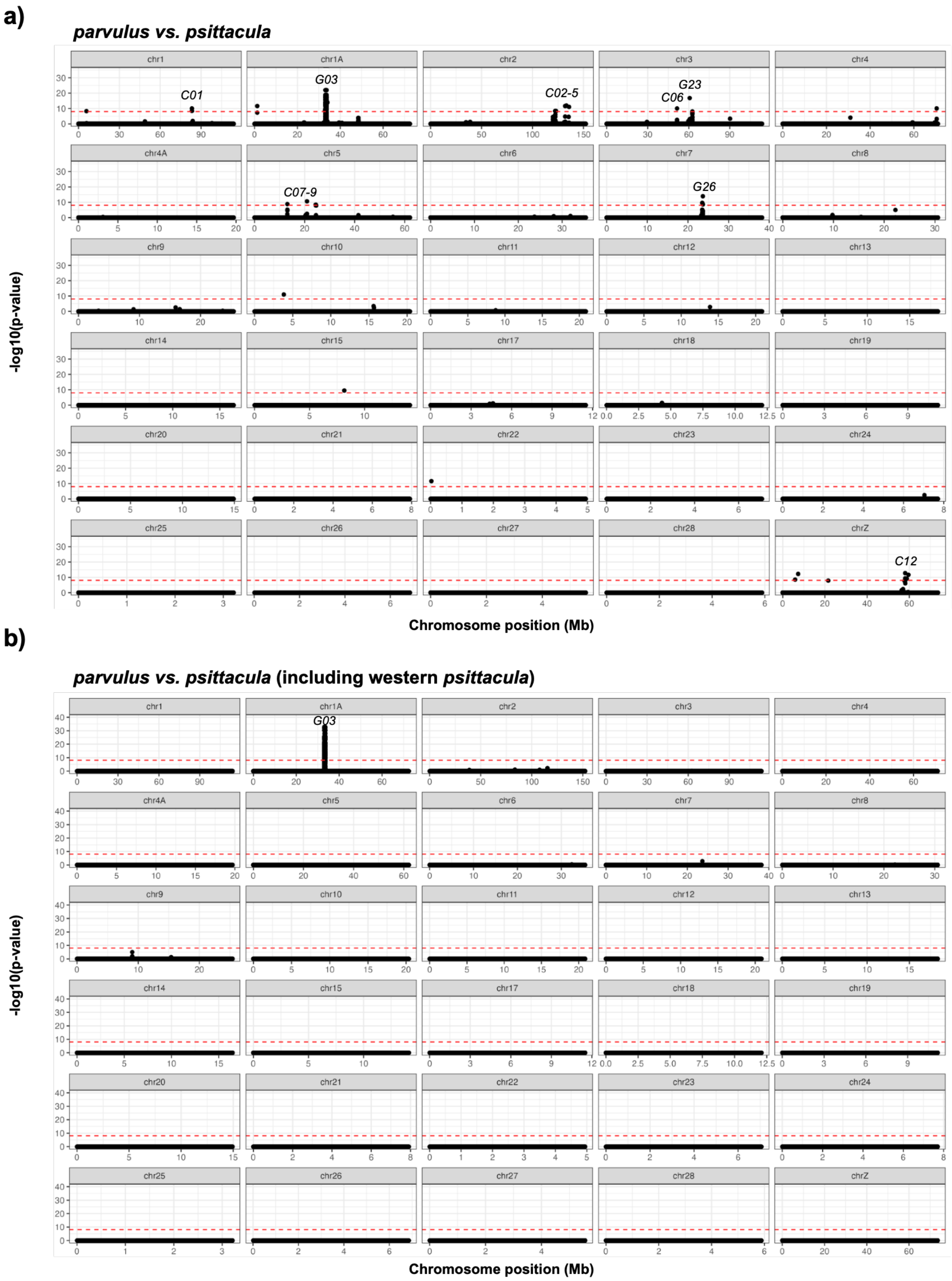

Fig. S14: Genome-wide admixture mapping between small and large tree finch. Regions of association resulting from an admixture mapping analysis between *parvulus* (coded as 0) and *psittacula* (coded as 1). The western *psittacula* lineage is excluded from the *psittacula* group in panel **a)** whereas it is included in **b)**. Divergence loci highlighted in previous studies on *Geospiza* ground finches follow the naming convention used in Rubin and Enbody *et al.* 2022 and Enbody *et al.,* 2023 (*i.e. G#*), whereas those differentiating tree finch taxa are named after *Camarhynchus* (*i.e. C#*). The red dashed line shows the significance threshold (α=0.05). Individual data points represent *p*-values after Bonferroni correction.

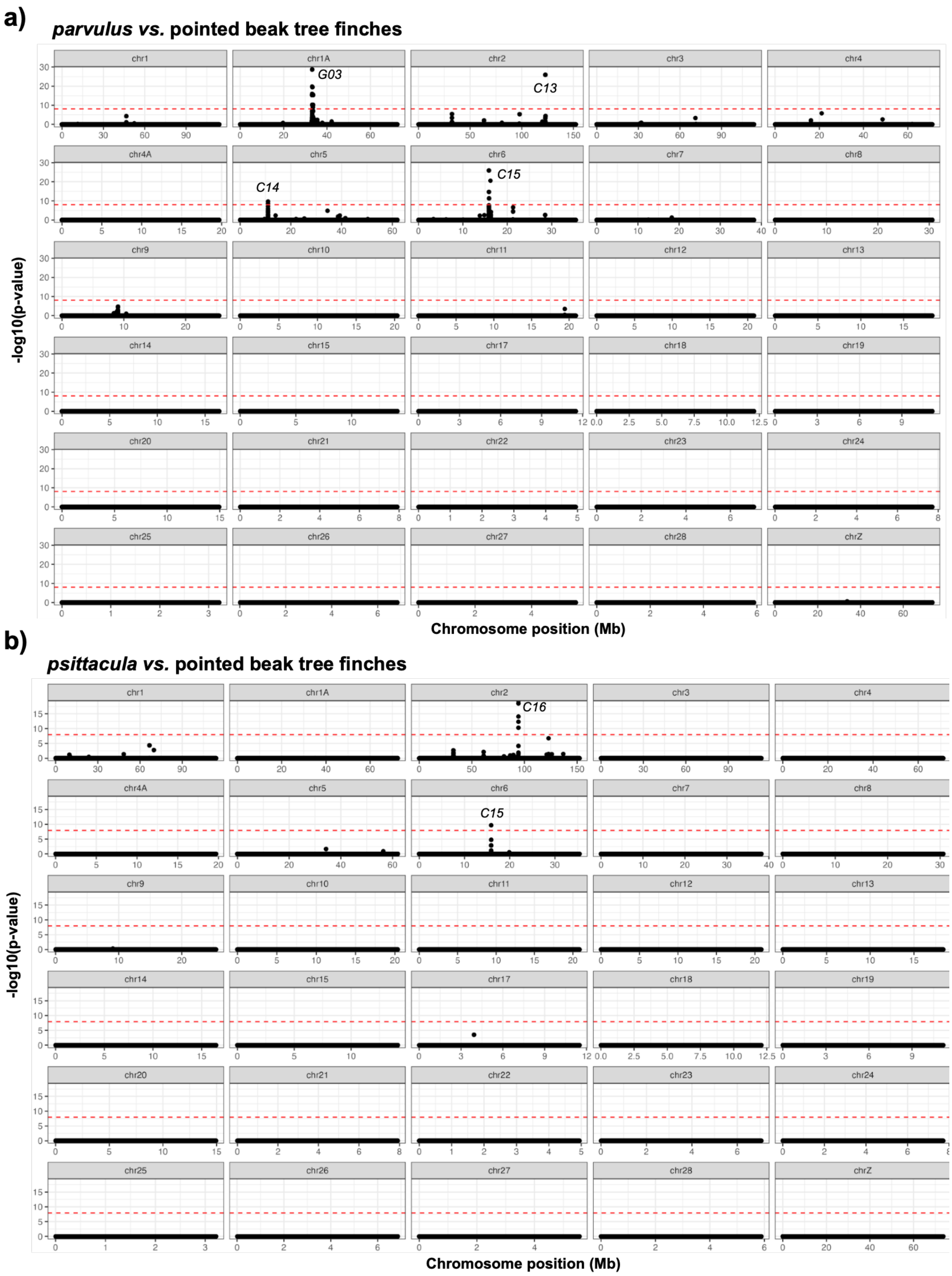

Fig. S15: Genome-wide admixture mapping between blunt beak (large and small tree finches) and pointed beak tree finches (woodpecker and mangrove finches). Regions of association resulting from an admixture mapping analysis between **a)** *parvulus* (coded as 0) and the pointed beak tree finches (*pallidus* and *heliobates,* coded as 1) and **b)** *psittacula* (coded as 0, western *psittacula* excluded) and the pointed beak tree finches (*pallidus* and *heliobates* coded as 1). Divergence loci highlighted in previous studies on *Geospiza* ground finches follow the naming convention used in Rubin and Enbody *et al.* 2022 and Enbody *et al.,* 2023 (*i.e. G#*), whereas those differentiating tree finch taxa are named after *Camarhynchus* (*i.e. C#*). The red dashed line shows the significance threshold (α=0.05). Individual data points represent *p*-value*s* after Bonferroni correction.

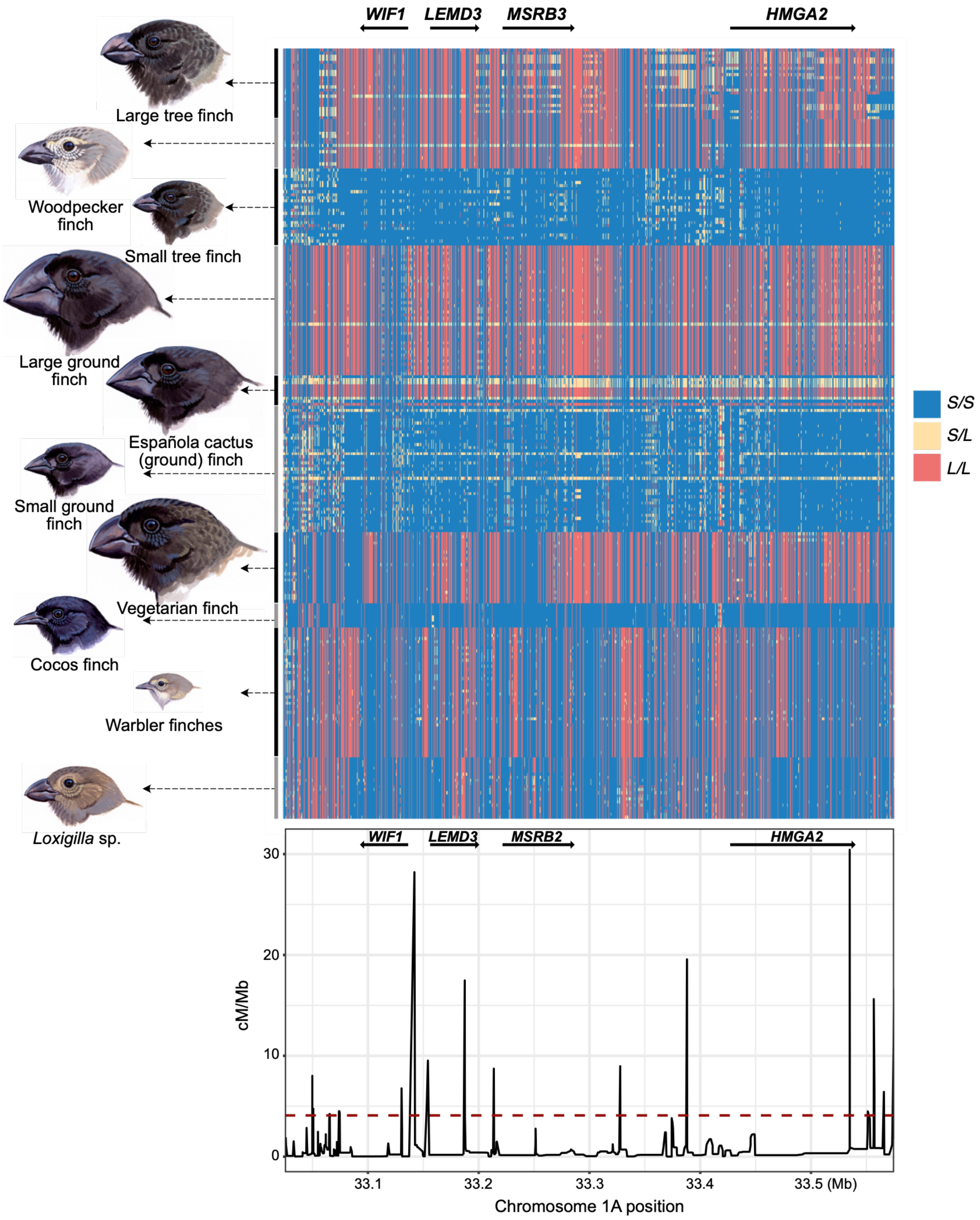

Fig. S16: Haplotype diversity at the *G03* locus among Darwin's finches. Overview of G03 haplotypes across the Darwin' finch radiation in the context of recombination rates (cM/Mb) across G03 (Chr1A:33,05-33,55 Mb) estimated by Rubin *et al*. 2022. The blue colour indicates homozygosity to the *C. parvulus* allele (S - small), whereas red indicates homozygosity for the alternative (L - large) allele. The red dashed line indicates the average recombination rate across chromosome 1A. Bird illustrations by Szabolcs Kókay.

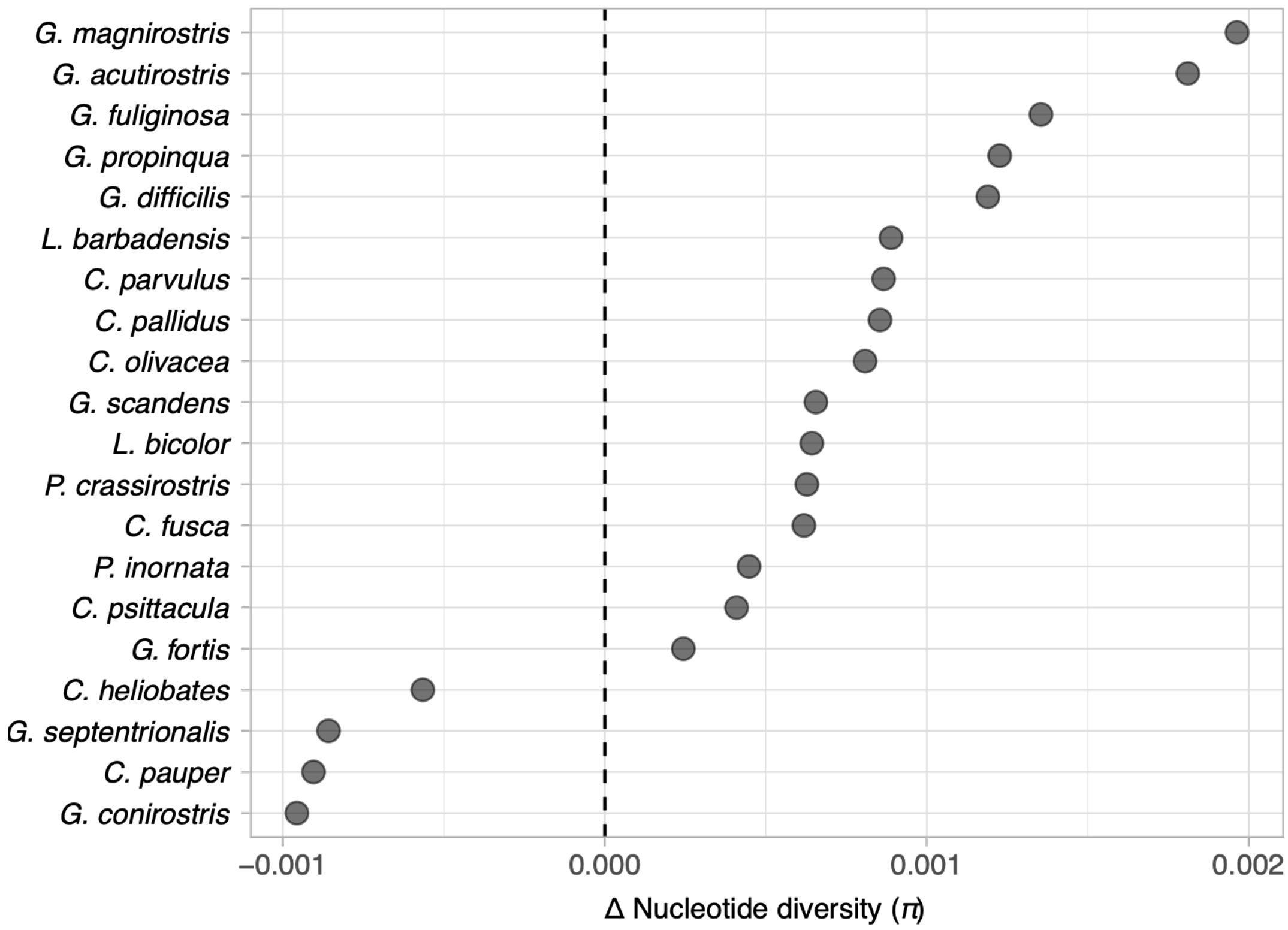

Fig. S17: Difference in nucleotide diversity between the *G03* locus and the chromosomal background across Darwin's finches. For each species, data points represent the difference between chromosome-wide nucleotide diversity (chr. 1A) and mean nucleotide diversity (π) within *G03*. Positive values indicate reduced diversity within *G03* relative to the chromosomal background, whereas negative values indicate high diversity.

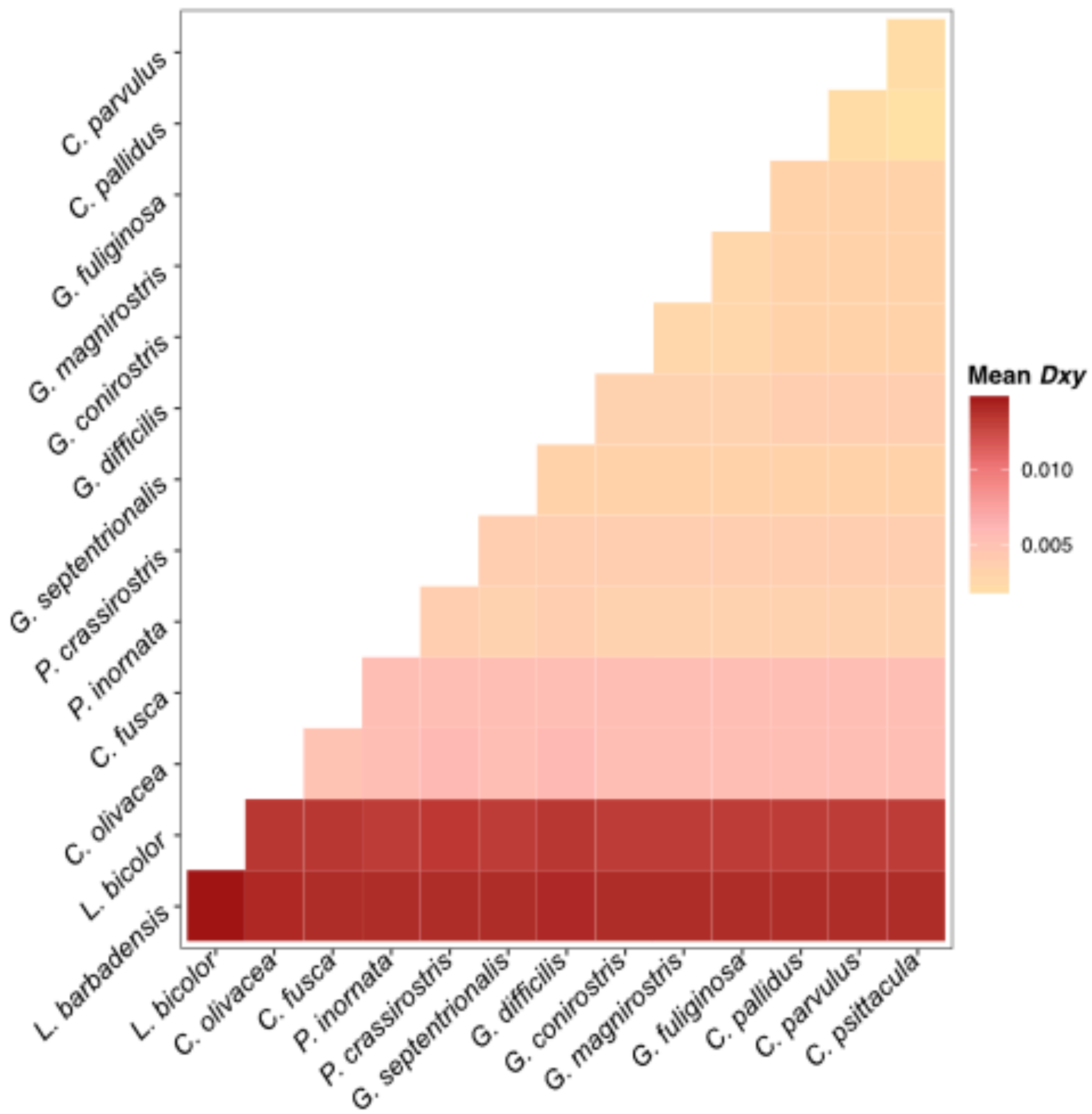

Fig. S18: Patterns of absolute divergence across the Darwin's finch radiation. Pairwise absolute divergence (*dxy*) genome-wide among selected species representing the major groups in the Darwin's finch radiation.

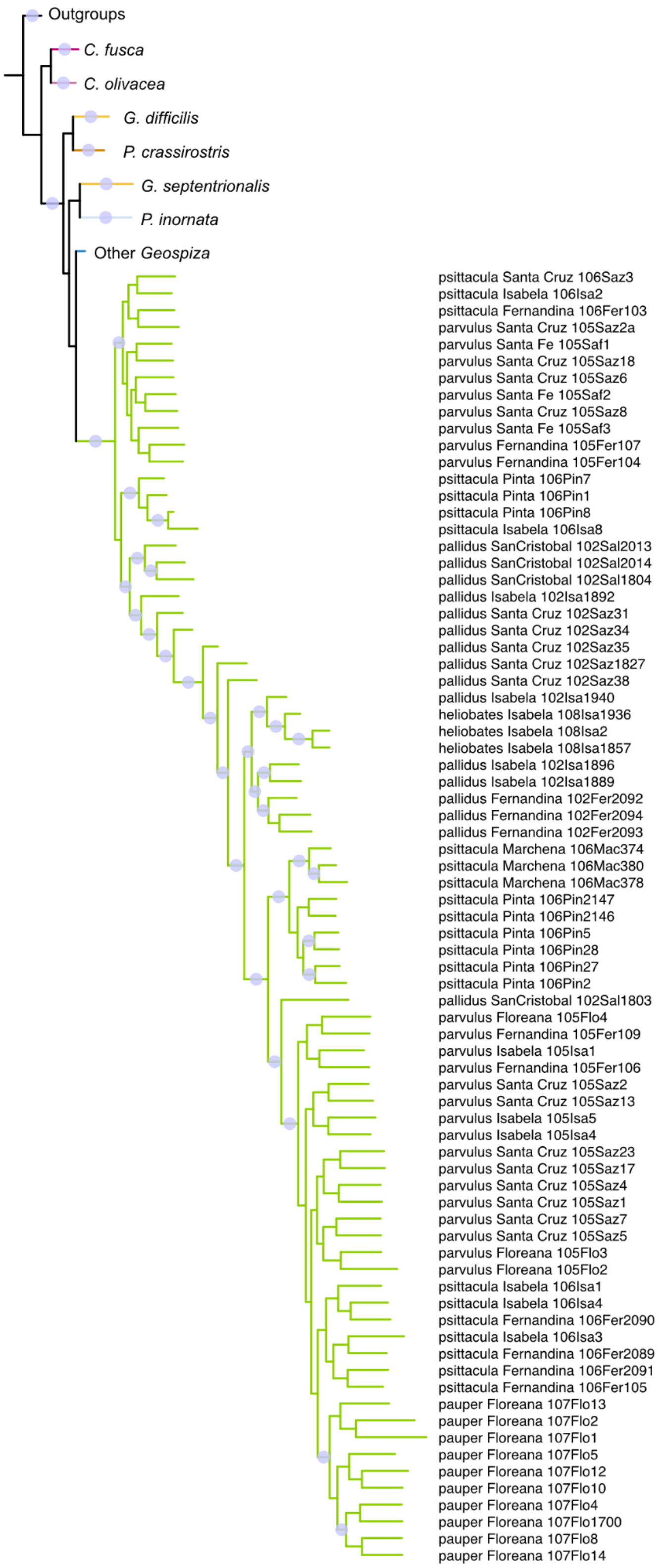

Fig. S19: Chromosome Z phylogeny of Darwin's finches. Maximum-likelihood phylogeny of the Darwin's finch radiation. Violet circles denote fully supported nodes.

Tables

Table S1: Factor loadings of principal components on the 348 *Camarhynchus* individuals measured from museum specimens. The upper panel **A)** represents the output of a principal component analysis (PCA) on beak length of the upper mandible, combined depth of both upper and lower mandible and width of the upper mandible, whereas **B)** refers to a PCA on wing and tarsus length, two proxies for body size.

**A)**	**PC1**	**PC2**	**PC3**
**St. dev.**	1.52	0.76	0.28
**% Variance**	77.6	19.7	2.6
**Length upper**	0.5	-0.85	-0.15
**Depth combined**	0.6	0.46	-0.65
**Width upper**	0.63	0.23	0.74
**B)**	**PC1**	**PC2**	
**St. dev.**	1.35	0.4	
**% Variance**	92	8	
**Wing**	-0.7	-0.71	
**Tarsus**	-0.7	0.7	

Table S2: Pairwise divergence time estimates. Average divergence times in years (T) for each pairwise comparison among the nine *Camarhynchus* taxa: *C. pallidus* S. Cruz (*C. pal. pal*), *C. pallidus* S. Cristóbal (*C. pal. str.*), western *pallidus* (*C. pal. pro*), *C. heliobates* (*C. hel.*), *C. parvulus* (*C. par.*), *C. pauper* (*C. pau.*), W *psittacula* (*C. psi. aff.*), *central psittacula* (*C. psi. psi*) and northern *psittacula* (*C. psi. hab.*). We also report split times between the *Loxigilla* outgroups and Darwin's finches with large and small beaks based on the *G03* locus. Finally, we also report estimated divergence time among all the main groups of Darwin's finches. Divergence times were estimated based on the number of nucleotide substitutions while accounting for the intrapopulation nucleotide diversity ($d\pi x$ and dy) as $da = dxy - (dx + dy)/2$, from which the divergence time was calculated as $da/2\mu$, with $\mu = 2.04 \times 10^{-9}$ [1,47,48].

Pop. 1	Pop. 2	T (yrs)	*dxy*%	Region
**Pairwise divergence time: pointed vs. blunt beak tree finches**				
C. hel.	***C. psi. hab.***	102 468	0.166	autosomes
C. hel.	***C. pau.***	89 538	0.176	autosomes
C. hel.	***C. par.***	86 307	0.184	autosomes
C. hel.	***C. psi. aff.***	85 018	0.166	autosomes
C. pal. pro.	***C. psi. hab.***	67 435	0.160	autosomes
C. pal. str.	***C. psi. hab.***	61 471	0.148	autosomes
**Pairwise divergence time between the outgroups and selected species based on *G03***				
C. psittacula	***C. parvulus***	1 116 694	0.570	*G03*
G. fuliginosa	***G. magnirostris***	954 223	0.495	*G03*
L. barbadensis	***C. parvulus***	2 075 612	0.974	*G03*
L. barbadensis	***G. fuliginosa***	1 838 051	0.879	*G03*
L. barbadensis	***G. magnirostris***	1 842 136	0.789	*G03*
L. barbadensis	***C. psittacula***	1 925 049	0.846	*G03*
L. noctis	***G. fuliginosa***	1 119 121	0.645	*G03*
L. noctis	***C. parvulus***	1 309 992	0.663	*G03*
L. noctis	***G. magnirostris***	1 154 659	0.626	*G03*
L. noctis	***C. psittacula***	1 231 361	0.582	*G03*
**Phylogenetic split time between the main groups of Darwin's finches**				
L. barbadensis	***C. parvulus***	2 932 103	1.396	autosomes
L. noctis	***C. parvulus***	2 483 096	1.328	autosomes
C. fusca	***C. parvulus***	849 753	0.535	autosomes
C. olivacea	***C. parvulus***	838 922	0.565	autosomes
P. inornata	***C. parvulus***	501 540	0.343	autosomes
P. crassirostris	***C. parvulus***	483 417	0.360	autosomes
G. septentrionalis	***C. parvulus***	357 694	0.317	autosomes
G. difficilis	***C. parvulus***	380 174	0.352	autosomes
G. conirostris	***C. parvulus***	287 807	0.313	autosomes
G. conirostris	***C. pallidus***	361 842	0.314	autosomes
G. conirostris	***C. heliobates***	334 389	0.310	autosomes

Table S3: **Gene content at *Fst* divergence peaks**. The table summarises information about the gene content present within the *Fst* divergence peaks resulting from the pairwise contrasts of blunt beak tree finches (marked with a red star in Fig. S8). The table contains information on the chromosome location, the range of positions spanned (in Mb), the peak ID, the size of the locus (in kb), gene name and its start and end positions.

Chr.	Peak range (Mb)	Peak ID	Locus size (kb)	Gene	Gene start	Gene end
1A	33,05-33,55	p1	500	*WIF1*	33,089,099	33,127,862
1A	33,05-33,55	p1	500	*LEMD3*	33,145,932	33,186,441
1A	33,05-33,55	p1	500	*MSRB3*	33,209,829	33,269,045
1A	33,05-33,55	p1	500	*HMGA2*	33,408,202	33,520,236
5	24,30-24,90	p1	600	*DLL4*	24,374,593	24,385,306
5	24,30-24,90	p1	600	*ENSCPVG00005007291*	24,413,002	24,414,268
5	24,30-24,90	p1	600	*INO80*	24,427,712	24,483,887
5	24,30-24,90	p1	600	*CHP1*	24,509,194	24,528,693
5	24,30-24,90	p1	600	*OIP5*	24,548,593	24,552,174
5	24,30-24,90	p1	600	*ENSCPVG00005007452*	24,552,432	24,563,664
5	24,30-24,90	p1	600	*NDUFAF1*	24,566,873	24,573,984
5	24,30-24,90	p1	600	*RFT1*	24,574,017	24,599,748
5	24,30-24,90	p1	600	*C14orf28*	24,647,625	24,649,044
5	24,30-24,90	p1	600	*ITPKA*	24,664,869	24,702,744
5	24,30-24,90	p1	600	*ENSCPVG00005007663*	24,709,136	24,725,392
5	24,30-24,90	p1	600	*Itk*	24,725,768	24,798,265
5	24,30-24,90	p1	600	*RPAP1*	24,858,057	24,892,151
6	16,90-16,98	p1	80	*SCD*	16,851,848	16,873,278
6	16,90-16,98	p1	80	*PKD2L1*	16,888,038	16,904,321
6	16,90-16,98	p1	80	*ABCG2*	16,915,998	16,932,751
6	16,90-16,98	p1	80	*BLOC1S2*	16,933,348	16,936,992
6	16,90-16,98	p1	80	*CYP2C23*	16,940,270	16,947,152

6	16,90-16,98	p1	80	*ALOX5*	16,963,749	16,988,430
Z	19,64-19,84	p1	200	*LOX*	19,647,492	19,658,168
Z	19,64-19,84	p1	200	*SRFBP1*	19,665,144	19,733,635
Z	31,48-31,92	p2	440	*ADAMTS19*	31,572,051	31,702,311
Z	31,48-31,92	p2	440	*ENSCPVG00005016319*	31,716,619	31,717,897
Z	31,48-31,92	p2	440	*CHSY3*	31,740,791	31,878,337
Z	36,95-37,55	p3	600	*TUT7*	36,989,261	37,022,159
Z	36,95-37,55	p3	600	*ISCA1*	37,026,264	37,031,100
Z	36,95-37,55	p3	600	*GOLM1*	37,052,974	37,086,527
Z	36,95-37,55	p3	600	*NAA35*	37,090,404	37,115,215
Z	36,95-37,55	p3	600	*AGTPBP1*	37,125,196	37,172,188
Z	36,95-37,55	p3	600	*NTRK2*	37,341,598	37,533,499
Z	57,01-57,12	p4	120	*FST*	57,006,411	57,012,811

Table S4. Most significant SNP positions identified for each divergence locus in the admixture mapping analysis. For each locus, the chromosome, genomic position and the population contrast (*parvulus* = par, *psittacula* = psi, pointed beak tree finches = PBTF) are shown. Genes where SNPs map are also shown, together with their genomic coordinates (start and end).

Chr.	Top SNP position (bp)	Contrast	Gene	Gene start	Gene end	Locus name
1	83,252,826	par_psi				*C01*
1A	> 10 snps	par_psi				*G03*
2	122,231,824	par_psi	*AHR*	122,219,430	122,278,407	*C02*
2	132,010,686	par_psi	*CACNB2*	131,955,197	132,193,442	*C03*
2	133,187,806	par_psi	*ENSCPVT00005025472*	133,001,177	133,243,947	*C04*
2	135,817,066	par_psi	*MKX*	135,773,251	135,822,542	*C05*
3	51,466,121	par_psi				*C06*
3	60,667,801	par_psi				*G23*
3	62,666,745	par_psi				*G23*
5	13,082,864	par_psi	*ABCC8*	13,082,864	12,128,633	*C07*
5	20,937,551	par_psi				*C08*
5	24,517,668	par_psi	*CHP1*			*C09*
5	24,526,138	par_psi	*CHP1*			*C09*
7	23,660,357	par_psi	*IGFBP2*	23,625,949	23,681,534	*G26*
Z	58,037,800	par_psi				*C12*
1A	> 10 snps	par_PBTF				*G03*
2	122,880,507	par_PBTF				*C13*
5	10,989,191	par_PBTF				*C14*
6	15,869,606	par_PBTF				*C15*
2	94,243,872	psi_PBTF				*C16*
2	122,870,129	psi_PBTF				*C13*
6	15,902,476	psi_PBTF				*C15*

References

1. Rubin, C.-J. *et al.* Rapid adaptive radiation of Darwin's finches depends on ancestral genetic modules. *Sci. Adv.* **8**, eabm5982 (2022).
2. Enbody, E. D. *et al.* Community-wide genome sequencing reveals 30 years of Darwin's finch evolution. *Science* **381**, eadf6218 (2023).
3. Lamichhaney, S. *et al.* A beak size locus in Darwin's finches facilitated character displacement during a drought. *Science* **352**, 470–474 (2016).
4. Pfannkuche, K., Summer, H., Li, O., Hescheler, J. & Dröge, P. The high mobility group protein HMGA2: a co-regulator of chromatin structure and pluripotency in stem cells? *Stem Cell Rev.* **5**, 224–230 (2009).
5. Zhou, X., Benson, K. F., Ashar, H. R. & Chada, K. Mutation responsible for the mouse pygmy phenotype in the developmentally regulated factor HMGI-C. *Nature* **376**, 771–774 (1995).
6. Weedon, M. N. *et al.* A common variant of HMGA2 is associated with adult and childhood height in the general population. *Nat. Genet.* **39**, 1245–1250 (2007).
7. Yang, T.-L. *et al.* HMGA2 is confirmed to be associated with human adult height: Replication between HMGA2 and height. *Ann. Hum. Genet.* **74**, 11–16 (2010).
8. Pandey, S. M. Chromatin remodeling complexes: The regulators of genome function. *Global Journal of Zoology* **1**, 007–013 (2016).
9. Guo, D.-C. *et al.* Loss-of-function mutations in YY1AP1 lead to Grange syndrome and a fibromuscular dysplasia-like vascular disease. *Am. J. Hum. Genet.* **100**, 21–30 (2017).
10. Shukla, S. *et al.* Conformational switching of Arp5 subunit regulates INO80 chromatin remodeling. *Nucleic Acids Res.* **53**, gkae1187 (2025).
11. Watanabe, K. P. *et al.* Avian cytochrome P450 (CYP) 1-3 family genes: isoforms, evolutionary relationships, and mRNA expression in chicken liver. *PLoS One* **8**, e75689 (2013).
12. Zhao, M. M. *et al.* Fatty acids modulate the expression of pyruvate kinase and arachidonate-lipoxygenase through PPARγ/CYP2C45 pathway: a link to goose fatty liver. *Poult. Sci.* **98**, 4346–4358 (2019).

13. Yang, S., Wang, W. & Ni, S. *G0S2* inhibits adipogenesis and unsaturated fatty acid biosynthesis by repressing the expression of *SCD* in chicken. *Int. J. Food Sci. Technol.* **58**, 2518–2526 (2023).
14. Chen, L. *et al.* Two cis-regulatory SNPs upstream of ABCG2 synergistically cause the blue eggshell phenotype in the duck. *PLoS Genet.* **16**, e1009119 (2020).
15. Liu, H. *et al.* Genome-wide association and selective sweep analyses reveal genetic loci for FCR of egg production traits in ducks. *Genet. Sel. Evol.* **53**, 98 (2021).
16. Lack, D. *Darwin's Finches*. (Cambridge University Press, 1947).
17. Laczkó, R. & Csiszár, K. Lysyl oxidase (LOX): Functional contributions to signaling pathways. *Biomolecules* **10**, (2020).
18. Zhang, X., Azhar, G. & Wei, J. Y. Sirtuin-2 is regulated by serum response factor and p49/strap genes. *Innov. Aging* **1**, 898–898 (2017).
19. Brocker, C. N., Vasiliou, V. & Nebert, D. W. Evolutionary divergence and functions of the ADAM and ADAMTS gene families. *Hum. Genomics* **4**, 43–55 (2009).
20. Ishimaru, D., Sugiura, N., Akiyama, H., Watanabe, H. & Matsumoto, K. Alterations in the chondroitin sulfate chain in human osteoarthritic cartilage of the knee. *Osteoarthritis Cartilage* **22**, 250–258 (2014).
21. Wilson, D. G. *et al.* Chondroitin sulfate synthase 1 (Chsy1) is required for bone development and digit patterning. *Dev. Biol.* **363**, 413–425 (2012).
22. Lim, J. *et al.* Uridylation by TUT4 and TUT7 marks mRNA for degradation. *Cell* **159**, 1365–1376 (2014).
23. Gu, S., Huang, Q., Sun, C., Wen, C. & Yang, N. Transcriptomic and epigenomic insights into pectoral muscle fiber formation at the late embryonic development in pure chicken lines. *Poult. Sci.* **103**, 103882 (2024).
24. Jia, Y. *et al.* NTRK2 promotes sheep granulosa cells proliferation and reproductive hormone secretion and activates the PI3K/AKT pathway. *Animals (Basel)* **14**, 1465 (2024).
25. Perini, F., Cendron, F., Lasagna, E., Cassandro, M. & Penasa, M. Genomic insights into shank and eggshell color in Italian local chickens. *Poult. Sci.* **103**, 103677 (2024).
26. Dushyanth, K., Shukla, R., Chatterjee, R. N. & Bhattacharya, T. K. Expression and

polymorphism of Follistatin (FST) gene and its association with growth traits in native and exotic chicken. *Anim. Biotechnol.* **33**, 824–834 (2022).

27. Wasti, S. *et al.* Expression of follistatin is associated with egg formation in the oviduct of laying hens. *Anim. Sci. J.* **91**, e13396 (2020).
28. Lehtonen, P. K. *et al.* Candidate genes for colour and vision exhibit signals of selection across the pied flycatcher (Ficedula hypoleuca) breeding range. *Heredity (Edinb.)* **108**, 431–440 (2011).
29. Toomey, M. B. *et al.* A non-coding region near Follistatin controls head colour polymorphism in the Gouldian finch. *Proc. Biol. Sci.* **285**, 20181788 (2018).
30. Schulte, K. W., Green, E., Wilz, A., Platten, M. & Daumke, O. Structural basis for aryl hydrocarbon receptor-mediated gene activation. *Structure* **25**, 1025-1033.e3 (2017).
31. Tischkau, S. A. Mechanisms of circadian clock interactions with aryl hydrocarbon receptor signalling. *Eur. J. Neurosci.* **51**, 379–395 (2020).
32. Friedrich, S. R., Lovell, P. V., Kaser, T. M. & Mello, C. V. Exploring the molecular basis of neuronal excitability in a vocal learner. *BMC Genomics* **20**, 629 (2019).
33. Ma, W., Lu, Y. & Wang, C. Production performance, egg quality, and uterine gene expression for layers as affected by N-Carbamylglutamate supplementation. *Front. Vet. Sci.* **10**, 1110801 (2023).
34. Hosseinzadeh, S. *et al.* Whole genome analysis of selection associated with resistance to heat stress in chickens. *Sci. Rep.* **16**, 11726 (2026).
35. Anderson, D. M. *et al.* Mohawk is a novel homeobox gene expressed in the developing mouse embryo. *Dev. Dyn.* **235**, 792–801 (2006).
36. Zhuang, Z.-X., Chen, S.-E., Chen, C.-F., Lin, E.-C. & Huang, S.-Y. Genomic regions and pathways associated with thermotolerance in layer-type strain Taiwan indigenous chickens. *J. Therm. Biol.* **88**, 102486 (2020).
37. Liu, R. *et al.* Genome-wide association study identifies Loci and candidate genes for body composition and meat quality traits in Beijing-You chickens. *PLoS One* **8**, e61172 (2013).
38. Hosnedlova, B. *et al.* Associations between *IGF1, IGFBP2* and *TGFß3* genes polymorphisms and growth performance of broiler chicken lines. *Animals (Basel)* **10**, E800 (2020).

39. Guo, Y. *et al.* Evolutionary analysis and functional characterization reveal the role of the insulin-like growth factor system in a diversified selection of chickens (Gallus gallus). *Poult. Sci.* **102**, 102411 (2023).

40. Rostamzadeh Mahdabi, E., Esmailizadeh, A., Ayatollahi Mehrgardi, A. & Asadi Fozi, M. A genome-wide scan to identify signatures of selection in two Iranian indigenous chicken ecotypes. *Genet. Sel. Evol.* **53**, 72 (2021).

41. Sardiello, M., Cairo, S., Fontanella, B., Ballabio, A. & Meroni, G. Genomic analysis of the TRIM family reveals two groups of genes with distinct evolutionary properties. *BMC Evol. Biol.* **8**, 225 (2008).

42. Hughes, D. C. ZP genes in avian species illustrate the dynamic evolution of the vertebrate egg envelope. *Cytogenet. Genome Res.* **117**, 86–91 (2007).

43. Swanson, D. L., Sabirzhanov, B., Vandezande, A. & Clark, T. G. Seasonal variation of myostatin gene expression in pectoralis muscle of house sparrows (Passer domesticus) is consistent with a role in regulating thermogenic capacity and cold tolerance. *Physiol. Biochem. Zool.* **82**, 121–128 (2009).

44. King, M. O. *et al.* Phenotypic flexibility of skeletal muscle and heart masses and expression of myostatin and tolloid-like proteinases in migrating passerine birds. *J. Comp. Physiol. B* **185**, 333–342 (2015).

45. Dou, D. *et al.* Genome-wide association studies for growth traits in broilers. *BMC Genom. Data* **23**, 1 (2022).

46. Haq, F., Ahmed, N. & Qasim, M. Comparative genomic analysis of collagen gene diversity. *3 Biotech* **9**, 83 (2019).

47. Nei, M. *Molecular Evolutionary Genetics*. (Columbia University Press, New York Chichester, West Sussex, 1987). doi:10.7312/nei-92038-016.

48. Lamichhaney, S. *et al.* Evolution of Darwin's finches and their beaks revealed by genome sequencing. *Nature* **518**, 371–375 (2015).
