# Supplementary material for: A supergene and genomic mosaicism are associated with phenotypic evolution in Galápagos tree finches
Source: Nat Commun. 2026 Oct 11;17:10656. doi: 10.1038/s41467-026-78507-8 (PMC13655052; doi:10.1038/s41467-026-78507-8)
Supplement: Supplementary file 2 [file 41467_2026_78507_MOESM2_ESM.pdf]

nature portfolio

Peer Review File

A supergene and genomic mosaicism are associated with phenotypic evolution in Galápagos tree finches

Corresponding Author: Dr Matteo Sebastianelli Arbelaez

This file contains all reviewer reports in order by version, followed by all author rebuttals in order by version.

Version 0:

Reviewer comments:

Reviewer #1

(Remarks to the Author)
This is my first review of the manuscript entitled, “Introgression facilitates rapid evolution of Galapagos tree finches.” The authors combine whole genomes with beak morphology to further understand the origins and diversification of Camarhynchus tree finches. They analyzed 415 genomes of Darwin finches, including 77 representing the four tree finch species. It looks like all but 2 genomes are from previous research. They recover 9 outlier regions. Among these, they establish the G03 superfamily of genes as drivers of the tree finch diversification, which was previously shown to control beak and body morphology of tree finches.
In general, I thought the paper was well written. However, I had a hard time really finding the novelty of the work and understanding the connection between the morphology and genetic dataset. Some detailed comments/suggestions that I would like to see the authors clarify on:

1) Currently, it seems that the morphology was added to a largely genomic study.
a. Why didn’t the authors conduct any GWAS against all of the traits that they obtained? Or are none of the morphological data part of the genomic data? If not, perhaps the authors can randomly assign trait variables from respective species’ measurement distribution to compare against the genomic backgrounds? Doing so several times could provide a sort of “bootstrapping” regarding the identification of outlier regions within GWAS analyses. I think something like this would help bring the 2 datasets together in a more cohesive way than currently presented.
b. I would like to at least see trees compared based on morphological versus genetic traits. Doing so in a single figure would provide a more direct comparison.

2) Broader genomic foci:
a. Among items is the focus on G03. I understand why the authors focused on the G03 gene, but given that this gene has been focused on previously (i.e., Enbody et al. 2023), what is the “novelty” in this paper? I really think it would have been better to equally focus on all of the recovered “outliers” and use the above GWAS suggestion to more directly test for such association rather than just relying on past studies/work.
b. I think some more formal test of selection on the G03 (and other genes) would be beneficial and provide additional “novelty.” Specifically, you find that the G03 haplotypes are largely segregating by species, but it would be good to understand whether this is due to simple genetic drift or mutations that arise due to species-specific [directional] selection.
c. The authors discuss “mosaicism” as it relates to genealogical associations. It is odd to me that the authors did not at least do an ancestry analysis on the bi-allelic SNP dataset in ADMIXTURE or something equivalent. Why was this not done?
d. On that note, why not also do a PCA on the genetic data, for which the authors can do an association test with the PC values obtained from the morphology to showcase that genetics explains (or not) morphological variation, and towards that, what proportion of the morphological variation is explained by genetics (i.e., proxy for inheritance). Again, this would better tie the 2 datasets together.
3) Broadening the discussion: Currently, the discussion is very much bird centric, which is fine. However, I expected some discussion to include broader themes regarding speciation with gene flow and how this study fits into it.

I really think addressing or expanding on some of the above suggestions would make a stronger and a manuscript with broader appeal. I wish the authors good luck!

(Remarks on code availability)

Reviewer #2

(Remarks to the Author)
This review is a collaboration between myself and a graduate student as part of the Early Career Review program.
This article provides a lot of interesting information on the radiation timing of Camarhynchus finches, especially in the case of C. psittacula potentially being a polyphyletic group. I also greatly appreciated the emphasis provided in the discussion on how both ecological and genomic analyses should be considered when discussing taxonomic assignments. The main concern I have with this paper is that there were no whole-genome sequences obtained from C. psittacula on Floreana Island that were used in this study. Due to Floreana Island being the only island with established populations for all three blunt-beaked tree finches and the island being central to theories on the timeline of divergence, this paper is missing a key piece of evidence that is essential to providing support for genetic mosaicism, a divergence timeline, or taxonomic assignments.

Suggested Improvements
Major
The single largest issue with this paper is its framing around a biogeographical hypothesis regarding Floreana Island, yet not (at least as far as we can tell through reading) including any C. psittacula genomes from that population. This creates a major gap that must be addressed in one of two ways. The first option is to include at least one individual of this group from Floreana. Given that may be logistically difficult, the other option is to refocus the paper more tightly on the phenotypic/functional aspect, which is already very strong without the focus on the Floreana speciation hypothesis outlined in Fig 2. As is, however, the apparent lack of samples from that key location for one of the taxa makes the paper feel a lot weaker than it otherwise could.
Specific instances:
• Page 4, Line 84-87: “Only one island in the archipelago, Floreana Island, harbours three species of tree finches with blunt beaks. They are small, medium and large. Focussing on the medium and large species, Lack argued that the large tree finch (Camarhynchus psittacula) is so similar to the same species on Santa Cruz Island it must have arrived from there, and recently (Fig. 2).” Referring to this in the introduction without getting a whole-genome sequence for the large tree finch seems like an oversight. If the focus of this paper is on Camarhynchus, I think there should be an effort to obtain a WGS of C. psittacula for the Floreana population, especially since their origin on the island is of interest to the literature that is being cited.
This comes up again on Page 18, Line 397-401: it may be challenging to provide support for any origin structure for Camarhynchus, but a WGS of C. psittacula on Floreana may provide some missing insight.
Minor
• Issue throughout the paper: There’s a general inconsistency in the paper with whether species (both focus and otherwise) are referred to by their scientific name or their common name. I would stick with one or the other so the reader can remain familiar with what is being referred to.
• Line 84-85: “Only one island in the archipelago, Floreana Island, harbours three species of tree finches with blunt beaks. They are small, medium and large.” Provide the specific names or use other clarifying wording- the second sentence implies mere size descriptors.
• Line 132-135: “First, by using a phylogenetic network approach (Fig. 3a) we found that the woodpecker finch C. pallidus striatipecta from San Cristóbal (hereafter “pallidus San Cristóbal”), C. pallidus pallidus (from Santa Cruz), C. pallidus productus (from the western islands Isabela and Fernandina) and the mangrove finch C. heliobates from Isabela are…” This is very difficult to read. I would include a table that links each scientific name to the island they are on.
• Line 397-401: Speaking on the issues with attempting to assign taxonomy. I think more emphasis should be placed on how flaws in taxonomic classification arise when we attempt to give a black-and-white assignment to a system with grey areas. Would put a sentence referencing this in the opening paragraph of the discussion.
• Fig 4: C and D) Please provide indication of the threshold of statistical significance. This may necessitate showing C as a plot of -log(p) instead of raw FST. D) As it currently reads, the figure caption for this graph makes it sound as if no matter where the line fell, it would provide you with support for your hypothesis. I would clarify where on the graph the reader should be paying attention to, and how far the line needs to be from 0 for the results to be significant.
• Fig 5: The bottom panel of this figure does not provide improved clarity from how you described the results in the section above. It may be better suited as a supplementary figure
• Fig 6: Consider adding some indication of population and/or classification of bill size to contextualize the phylogeny.
• Line 422: “…we used 415 Darwin’s finch samples (including the 77 Camarhynchus…)”. Please provide in the Supplementary Materials some information, either as a list or map, of the exact numbers and locations of at least the 77 Camarhynchus samples.
• Line 434: “…which are analogous to…GATK 4.1”- is there a reason that these tools were used? It’s fine if it’s personal preference, but I’d be curious if there were specific issues with the more standard GATK tools that make alternates more suitable for these data.
• Line 471: “To perform these diversity statistics, two individuals were removed…” Particularly with the hybrid individual, was there an observable difference in these measurements with and without? If removal of an F1 is justified, what about individuals of hybrid ancestry more distantly?
• Line 548: “We use 348 morphological measurements…” It sounds like you used 348 individuals based on line 556, and you state you performed multiple measurements per specimen- which would make your number of measurements substantially higher than 348, correct? Also, as with genetic samples, it would be advisable to provide a supplementary table with these data and specimen information, along with whether any individuals were included in both datasets (I assume not

given the description of taking blood samples, but it is not entirely clear whether that rules out any of those do have a specimen associated with them if the individual later died or was collected?)
• Line 711, Reference 36: “O’connor, J.A.,” C needs to be capitaliazed

(Remarks on code availability)

Reviewer #3

(Remarks to the Author)
I co-reviewed this manuscript with one of the reviewers who provided the listed reports. This is part of the Nature Communications initiative to facilitate training in peer review and to provide appropriate recognition for Early Career Researchers who co-review manuscripts.

(Remarks on code availability)

Version 1:

Reviewer comments:

Reviewer #1

(Remarks to the Author)
This is my second review of “A supergene and genomic mosaicism are associated with phenotypic evolution in Galápagos tree finches.” I want to start by thanking the authors for doing a great job at all the comments. I only have a few minor comments for the abstract, otherwise, I think the authors did a great job explaining all of the methods, how and where the different datasets came from, and how they were used to draw your inferences. Great work!

Abstract - minor edits.
Line 25-26 - Suggest just stating “Camarhynchus diversified over the last 100,000 years with striking morphological differences despite shallow genetic divergence.”

Line 27-27 – Suggest restating “We identified several highly differentiated loci, including G03 that is a supergene affecting beak and body size.”

Line 34-35 – Suggest edit “ Our results further establish…”

(Remarks on code availability)

Reviewer #3

(Remarks to the Author)
I co-reviewed this manuscript with one of the reviewers who provided the listed reports. This is part of the Nature Communications initiative to facilitate training in peer review and to provide appropriate recognition for Early Career Researchers who co-review manuscripts.

(Remarks on code availability)

Open Access This Peer Review File is licensed under a Creative Commons Attribution 4.0 International License, which permits use, sharing, adaptation, distribution and reproduction in any medium or format, as long as you give appropriate credit to the original author(s) and the source, provide a link to the Creative Commons license, and indicate if changes were made.
In cases where reviewers are anonymous, credit should be given to 'Anonymous Referee' and the source.
The images or other third party material in this Peer Review File are included in the article's Creative Commons license, unless indicated otherwise in a credit line to the material. If material is not included in the article's Creative Commons license and your intended use is not permitted by statutory regulation or exceeds the permitted use, you will need to obtain permission directly from the copyright holder.
To view a copy of this license, visit https://creativecommons.org/licenses/by/4.0/

We thank the reviewers for their constructive criticism of our paper that has helped us to improve the paper. Below we provide a point-to-point response to all comments.

#### Reviewer 1
This is my first review of the manuscript entitled, "Introgression facilitates rapid evolution of Galapagos tree finches." The authors combine whole genomes with beak morphology to further understand the origins and diversification of Camarhynchus tree finches. They analyzed 415 genomes of Darwin finches, including 77 representing the four tree finch species.
It looks like all but 2 genomes are from previous research.
They recover 9 outlier regions.
Among these, they establish the G03 superfamily of genes as drivers of the tree finch diversification, which was previously shown to control beak and body morphology of tree finches.
In general, I thought the paper was well written.
However, I had a hard time really finding the novelty of the work and understanding the connection between the morphology and genetic dataset.

We would like to clarify that the focus of our study was on genomic and morphological variation within the tree finch system. To investigate this, we leveraged a previously generated genomic dataset to investigate the evolutionary dynamics within this lineage, which were never investigated in our previous publications. In parallel, we assessed morphological variation using morphological measurements from >300 museum specimens measured by Prof. Peter Grant.

Even though these two datasets are not connected, their integration in our study allowed us to improve our understanding of the evolutionary relationships as well as genomic architecture of phenotypic differences within *Camarhynchus*.

There are several aspects of this study that are novel besides the population genetic analysis (within and among subspecies) of this group of Darwin's finches that was never studied before from a genomic perspective. The main findings of the paper are (a) the very close genetic relationship among the tree finches, (b) genomic mosaicism of the *C. psittacula affinis* lineage (c) the key role for the *G03* locus for phenotype diversification and (d) poor correspondence between whole-genome phylogeny and the current morphology-based taxonomy. The finding of genomic mosaicism of the *C. psittacula affinis* lineage is new and completely unexpected. The *C. psittacula affinis* lineage shows morphological resemblance to *C. pauper* (the medium tree finch) (Fig. 1c, 3a), has high genomic affinity with *C. parvulus* (the small tree finch), but is currently classified as *C. psittacula*. Interestingly, the *G03* haplotype of *C. psittacula affinis* is equally similar to that of the woodpecker finch *C. pallidus* and to that of *C. psittacula* (Fig. S16), questioning its current taxonomic status and highlighting the complex evolutionary history of this lineage. However, from an evolutionary perspective, this discovery reveals a novel way in which a species can be transformed genomically by hybridization and complements the extensive work on speciation in the ground finches.

Also, the finding that *C. psittacula affinis* has a mixed ancestry (together with the feedback received by reviewer 1) prompted us to revisit GWAS mapping among tree finches previously reported in our Rubin *et al*. 2021 paper. By excluding the *C. psittacula affinis* we now reveal much clearer differentiation between small and large tree finches. In revisiting the GWAS, we also compare the latter two lineages with the

woodpecker/mangrove finch lineage. Altogether, we report 15 QTLs associated with genetic differentiation among tree finches that were previously not reported (Fig. S14-15, Table S4).

We believe that these points push forward our understanding of the relationships and evolutionary dynamics within the finch radiation. We have revised the manuscript to highlight their relevance. We also highlight these points at the end of the introduction (L108-115) and revisited the title accordingly.

Some detailed comments/suggestions that I would like to see the authors clarify on:
1) Currently, it seems that the morphology was added to a largely genomic study.

a. Why didn't the authors conduct any GWAS against all of the traits that they obtained? Or are none of the morphological data part of the genomic data?

The two datasets are not connected. The samples in our genetic dataset do not have associated morphological measurements and none of the museum specimens has associated DNA data. The genomic dataset results from the sequencing of samples collected by Profs. Rosemary and Peter Grant and their team during a decades-long field study on Galapagos, whereas the morphological dataset results from the measurements obtained from >300 museum specimens.

If not, perhaps the authors can randomly assign trait variables from respective species' measurement distribution to compare against the genomic backgrounds? Doing so several times could provide a sort of "bootstrapping" regarding the identification of outlier regions within GWAS analyses. I think something like this would help bring the 2 datasets together in a more cohesive way than currently presented.

In agreement with the reviewer's comments, we have revisited a GWAS analyses that were performed in Rubin *et al*., 2022 (Supplementary Figure 5). In that study, the analyses were performed by grouping individuals from all populations to increase the sample size and statistical power of the GWAS. In light of the genetic mosaicism of *C. psittacula affinis*, we have now decided to conduct the GWAS analysis again by removing this lineage from the large tree finch group because of its genetic similarity with *C. parvulus* across autosomes. Furthermore, we also compared the two extreme phenotypes of the blunt beak tree finches (*parvulus* and *psittacula*) with the pointed beak tree finches (*pallidus* and *heliobates*). The GWAS results have highlighted additional loci that differentiate *parvulus* and *psittacula*, but also a locus on chr. 6 that is unique to pointed beak tree finches and one on chr. 2 that differentiates the two large linages (*psittacula* and pointed beak tree finches). We have added a new section in the main text (*Admixture mapping reveals loci underlying genomic differentiation among tree finches*, L292-310) and describe the gene content at GWAS peaks in *Supplementary Note 4*. We have also added two supplementary figures (Fig. S14-15) and one supplementary table (Table S4) describing these results.

In Fig. S14b, we also include a GWAS between *parvulus* and *psittacula*, with the latter group including the western *psittacula.* Besides *G03*, differentiation between the two groups is completely reduced below the significance threshold, reflecting the genetic similarity of western *psittacula* and *parvulus* genome-wide. Thus, we believe that reproposing a GWAS without western *psittacula* in the large tree finch group represents a significant improvement to our previous analyses.

b. I would like to at least see trees compared based on morphological versus genetic traits. Doing so in a single figure would provide a more direct comparison.

To facilitate integration of our morphological data with our genetic dataset, we leveraged the former to build Euclidean distance matrices on all morphological traits (body size + beak and beak only) and use these for neighbour-joining tree inference. We added the two cladograms in Fig. 3a, allowing a direct comparison with the phylogenetic network based on genetic distances (Fig. 3b). Interestingly, the cladograms in Fig. 3a highlight the ambiguous classification of *C. psittacula affinis*. In agreement with its current taxonomic status, *C. psittacula affinis* clusters with the other large tree finches based on beak morphology only, but when incorporating body size, most *C. psittacula affinis* individuals cluster close to the small tree finch *C. parvulus* (more specifically, between the medium tree finch *C. pauper* and the small tree finch *C. parvulus*, whereas based on beak measurements only it clusters between *C. pauper* and *C. psittacula*). Thus, mirroring its genetic mosaicism, *C. psittacula affinis* is slightly disproportionate, with a smaller body relative to its beak size. We thank the reviewer for this suggestion.

2) Broader genomic foci:
a. Among items is the focus on G03. I understand why the authors focused on the G03 gene, but given that this gene has been focused on previously (i.e., Enbody et al. 2023), what is the “novelty” in this paper? I really think it would have been better to equally focus on all of the recovered “outliers” and use the above GWAS suggestion to more directly test for such association rather than just relying on past studies/work.

Our focus on the *G03* locus is dictated by it being the only autosomal locus that reaches complete fixation in the comparison between small and large tree finches, a result in agreement with previous work from our group showing that *G03* alone accounts for about 25-30% of beak size variation in the *Geospiza* finches (Enbody *et al*., 2023). The novelty of our study lies in the evidence that this locus may account to most phenotypic differences between two sympatric species: *C. parvulus* and *C. psittacula affinis*, and that allelic variation at *G03* is responsible for the shift from one phenotypic optimum (small tree finch) to another (medium/large tree finch). Furthermore, we show that the similar phenotypes of *C. psittacula affinis* and *C. pauper* have different genetic architectures, with the phenotype of *C. psittacula affinis* arising through homozygosity for the L allele at *G03* together with a small tree finch-like genome, whereas *C. pauper* has intermediate frequencies for the L and S alleles at *G03*. A striking finding of the updated analysis is that the L *G03* allele in *C. psittacula affinis* is similar to that of *C. pallidus.* Hence, the genomic data we present questions the current taxonomic classification of *C. psittacula affinis* as a large tree finch. We have revisited the manuscript to highlight these points of novelty (see title, abstract and final paragraph of the discussion). In addition, the importance of *G03* was further corroborated by Z-transforming *Fst* (see response to reviewers 2 and 3), as well as in our GWAS results. Together, these analyses support that *G03* is the major driver of beak size differentiation among Darwin’s finches. In light of these findings, we expanded our comparison to other finch lineages, and report an unexpected diversity of haplotypes (figure 6a), which was never reported before and thus represents another point of novelty of our tree finch study. However, even though some parts of our study specifically focus on the evolutionary history of this locus, we thoroughly describe the gene content within other outlier loci detected with the *Fst* scans

and GWAS in supplementary notes 3 and 4. Furthermore, we complemented the above-mentioned analyses by investigating patterns of allele sharing between *C. psittacula* and *C. parvulus* (Fig 4d, S13). Together, these analyses resulted in the detection of new candidate genes that contribute to phenotypic diversity in this system.

b. I think some more formal test of selection on the G03 (and other genes) would be beneficial and provide additional "novelty." Specifically, you find that the G03 haplotypes are largely segregating by species, but it would be good to understand whether this is due to simple genetic drift or mutations that arise due to species-specific [directional] selection.

In responding to this comment, we assume the reviewer refers to Fig. 6a.

The analysis proposed by the reviewer would require the comparison of rates of non-synonymous and synonymous substitutions in coding regions (*i.e.* dN/dS) between different regions. Please note that there are no coding changes with strong differentiation between the S and L alleles and a possible investigation will have to be conducted on non-coding regions, but it is extremely challenging to identify species-specific adaptive, non-coding mutations over a 525 kb genomic region (*i.e. G03*) in non-model species with rudimentary functional annotation of non-coding regions. This is beyond the scope of our study. However, we have now added an analysis where we compare nucleotide diversity within the *G03* region compared with the genome average (L342-351, Fig. S17). This analysis shows that there is a strong trend for a reduction of nucleotide diversity in the *G03* region also in the two outgroup species included in this study. This most likely reflect the low recombination rate in this genomic region resulting in background selection when deleterious mutations are purged. Selection on beak size may add to low nucleotide diversity within species. Two species, *G. magnirostris* and *G. acutirostris*, have extremely low nucleotide diversity consistent with selective sweeps. Furthermore, we discuss the evolutionary history of the *G03* haplotypes on Line 437-441 of the Discussion and argue that the fact that *G03* S and L alleles have been maintained as 525 kb haplotype blocks over the last million years implies the accumulation of multiple causal variants across the region; there is no evidence for the presence of an inversion that could explain these extraordinary large haplotype blocks.

c. The authors discuss "mosaicism" as it relates to genealogical associations. It is odd to me that the authors did not at least do an ancestry analysis on the bi-allelic SNP dataset in ADMIXTURE or something equivalent. Why was this not done?

We believe that the use of ancestral recombination graphs (ARGs) to infer population affinity is another innovative aspect of our study from a methodological perspective. ARGs are increasingly being used to infer genetic admixture and detect introgression in both humans (Speidel et al. 2025; Peede et al. 2026) and non-model systems (Pal et al. 2025; Payseur et al. 2025) because of their power to capture the full coalescent history of each genomic region. In this regard, this approach has a leading edge over other more "traditional" methods that rely on genetic similarity (Mathieson and Scally 2020) as the ones mentioned by the reviewer. We believe that given the high levels of allele sharing found among finch lineages (Lamichhaney et al., 2015), and especially those find within *Camarhynchus* lineages, ARGs are better suited to detect fine-scale population structure and admixture. We now clarify our methodological choice in the methods section (L552-555). In addition to this, the genomic mosaicism of the *affinis* lineage is supported by

several other analyses showed in figure 4 (autosomal vs. *G03* trees, tree topology weighting analysis performed in TWISST, ABBA-BABA distance fraction and *ZFst* analysis) and 5 (delta allele frequency).

d. On that note, why not also do a PCA on the genetic data, for which the authors can do an association test with the PC values obtained from the morphology to showcase that genetics explains (or not) morphological variation, and towards that, what proportion of the morphological variation is explained by genetics (i.e., proxy for inheritance). Again, this would better tie the 2 datasets together.

A PCA on genetic data would group individuals based on genetic similarity and would not provide additional information to the phylogenetic network in Fig. 3b, which is based on genetic distance. We incorporate in this response to reviewer 1 a PCA plot on the genetic data. Like the phylogenetic network in Fig. 3b, *C. parvulus*, *C. pauper* and *C. psittacula affinis* form a tight cluster, reflecting their overall morphology (Fig. 3a, left cladogram). Therefore, to avoid redundancy, either the PCA plot or the phylogenetic network should be incorporated in the figure, and we believe that the network more explicitly represents the biological variation within these groups through its internal branches, whereas PCA does not have any underlying evolutionary model.

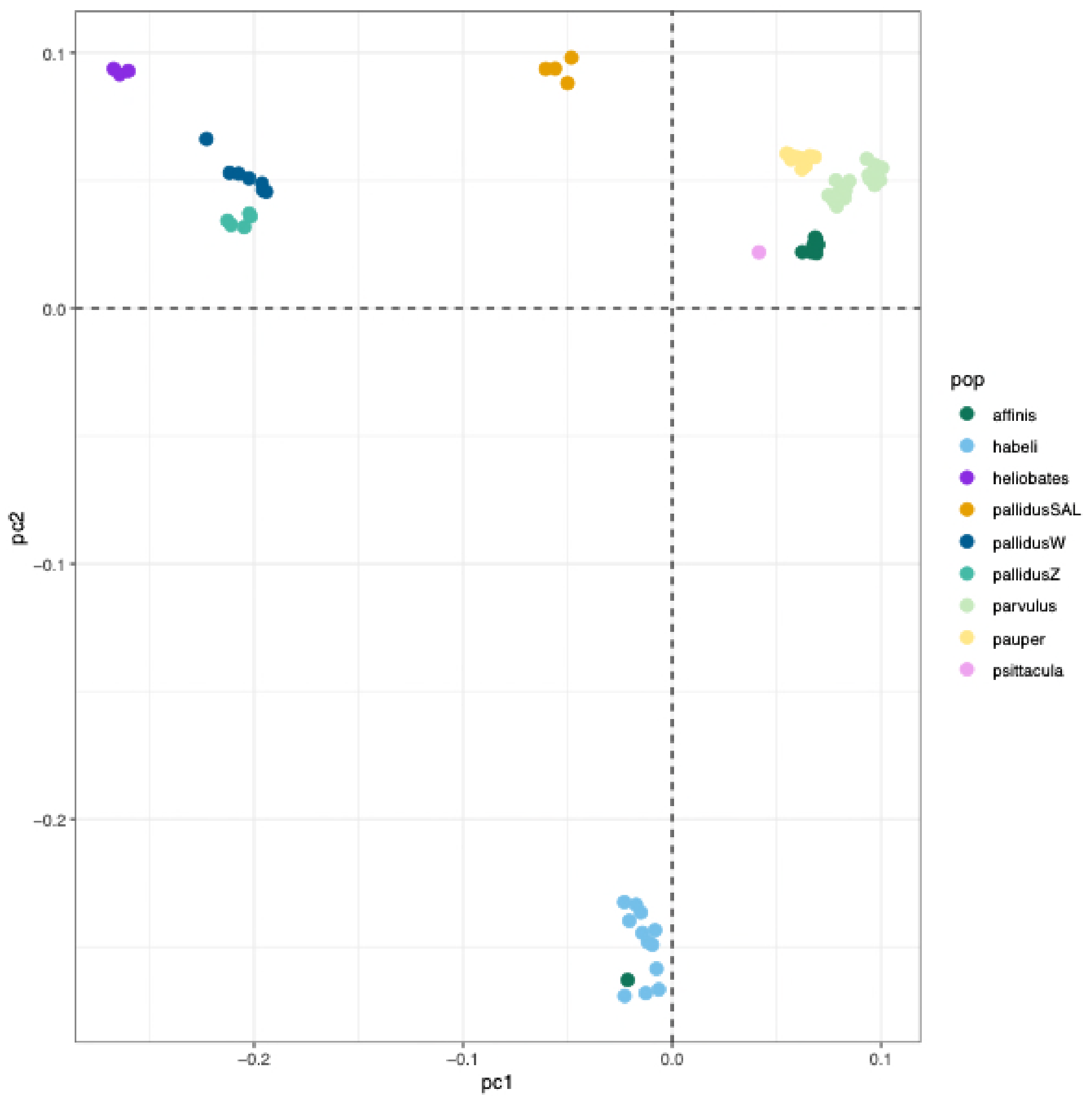

Furthermore, since the two datasets don't share the same individuals, a correlation of PCA scores (genetic and morphology) would require averaging by population and compare the population PC averages, which would be a bit difficult to interpret. In addition to this, a correlation between morphology and genetic similarity is not to be

expected, because similar ecological requirements could drive convergent phenotypes. For instance, *C. psittacula habeli* is morphologically more similar to *C. pallidus striatipecta* than to *C. parvulus* (Fig. 1c) despite being genetically more similar to the latter (Fig. 3c). In fact, Lack speculated that *C. psittacula habeli* and *C. pallidus striatipecta* converged in morphological space and ecological niche: *C. pallidus striatipecta* evolved a blunter beak because *C. psittacula* is absent on San Cristóbal and that *C. psittacula habeli* evolved a pointier beak in the northern islands because *C. pallidus* is absent there.

Overall, we agree that the lack of morphological measurements from the same individuals in the genetic dataset is a limitation of our study. We hope that visualizing the morphological cladograms (Fig. 3a) together with a phylogenetic network based on genetic distance (Fig. 3b) alleviates the reviewer concerns of making the two datasets more comparable.

3) Broadening the discussion: Currently, the discussion is very much bird centric, which is fine. However, I expected some discussion to include broader themes regarding speciation with gene flow and how this study fits into it.

We appreciate this suggestion and have added a statement in the final paragraph of the discussion explaining how our study fits a growing body of literature that highlights the important role of hybridization in speciation (L490-493).

I really think addressing or expanding on some of the above suggestions would make a stronger and a manuscript with broader appeal. I wish the authors good luck!

#### Reviewer 2 and 3
Reviewer #2 (Remarks to the Author):

This review is a collaboration between myself and a graduate student as part of the Early Career Review program.
This article provides a lot of interesting information on the radiation timing of Camarhynchus finches, especially in the case of C. psittacula potentially being a polyphyletic group. I also greatly appreciated the emphasis provided in the discussion on how both ecological and genomic analyses should be considered when discussing taxonomic assignments. The main concern I have with this paper is that there were no whole-genome sequences obtained from C. psittacula on Floreana Island that were used in this study. Due to Floreana Island being the only island with established populations for all three blunt-beaked tree finches and the island being central to theories on the timeline of divergence, this paper is missing a key piece of evidence that is essential to providing support for genetic mosaicism, a divergence timeline, or taxonomic assignments.

Suggested Improvements
Major
The single largest issue with this paper is its framing around a biogeographical hypothesis regarding Floreana Island, yet not (at least as far as we can tell through reading) including any C. psittacula genomes from that population. This creates a major gap that must be addressed in one of two ways. The first option is to include at least one individual of this group from Floreana. Given that may be logistically difficult, the other option is to refocus the paper more tightly on the

phenotypic/functional aspect, which is already very strong without the focus on the Floreana speciation hypothesis outlined in Fig 2. As is, however, the apparent lack of samples from that key location for one of the taxa makes the paper feel a lot weaker than it otherwise could.
Specific instances:
• Page 4, Line 84-87: "Only one island in the archipelago, Floreana Island, harbours three species of tree finches with blunt beaks. They are small, medium and large. Focussing on the medium and large species, Lack argued that the large tree finch (Camarhynchus psittacula) is so similar to the same species on Santa Cruz Island it must have arrived from there, and recently (Fig. 2)." Referring to this in the introduction without getting a whole-genome sequence for the large tree finch seems like an oversight.

If the focus of this paper is on Camarhynchus, I think there should be an effort to obtain a WGS of C. psittacula for the Floreana population, especially since their origin on the island is of interest to the literature that is being cited.
This comes up again on Page 18, Line 397-401: it may be challenging to provide support for any origin structure for Camarhynchus, but a WGS of C. psittacula on Floreana may provide some missing insight.

We thank reviewers 2 and 3 for their feedback on our manuscript and appreciate they find it interesting. We apologize for the confusion and acknowledge that in our introduction we failed to state clearly that *C. psittacula* on Floreana Island is long extinct as a breeding population. We now clarify that in the Introduction (L89-90). We therefore don't have DNA samples from this population, and a genomic study on this population would have to rely on ancient DNA extracted from museum specimens. I provide here more information on what is known about *C. psittacula* on Floreana. Drawing from Grant *et al*., 2005, "*C. psittacula* on Floreana its always been rare, and its rarity may underlie its late discovery in 1888 (Rothschild and Hartert, 1899), and the fact that an expedition in 1899 failed to find it (Snodgrass and Heller, 1904). The California Academy of Sciences collectors obtained only nine specimens in both the wet (May) and dry (October) seasons (Swarth, 1931). In fact, in his studies Lack analysed only 17 samples in total (Lack, 1945). In September 1957 Bowman (1961) observed an individual feeding in the highlands, and tape-recorded another in 1962 (Bowman, 1983). Steadman (1986) did not see it and listed it as possibly extinct". The Grants have never seen a *C. psittacula* in their five visits to Floreana. However, "Sonia Kleindorfer and her group captured three birds in a recent study and found a nest", but despite this, *C. psittacula* on Floreana is to be considered extinct (Kleindorfer et al., 2014).

Having clarified on the status of *C. psittacula* on Floreana, we have slightly modified the framing of our manuscript and shifted the focus towards *C. psittacula affinis*. This allowed us to maintain the historical backbone in our manuscript. Specifically, in his book, David Lack wondered about the classification of *C. psittacula affinis*. He had a parsimonious approach to species classification and preferred to focus on the affinities between lineages, rather than differences. Therefore, he considered *affinis* as a large tree finch because he had no evidence for the opposite. The opposite is true for the medium tree finch *C. parvulus*, that he had to consider as a separate species because it coexisted with the now extinct *C. psittacula* without (or limited) interbreeding (in his 1947 book, he writes: "*The distinctive island forms are here united under C. psittacula, except for C. pauper, which, though no more distinctive than the rest, must be treated as a separate species"*). However, given the phenotypic similarity between *C. pauper* and *C. psittacula*

affinis, Lack wondered whether the latter would also not interbreed with the other *C. psittacula*s, questioning its species status ("*If the Abermarle* (Isabela) *race affinis were now to colonize Abingdon* (Pinta), *where the form habeli lives* (northern psittacula), *the two are so distinctive that they might keep separate, in which case they would have to be classified as separate species. But such an invasion has not yet occurred, so that it is more convenient to consider affinis and habeli as races of the same species"*). Our study provides evidence that the morphological similarity of *C. pauper* and *C. psittacula affinis* has a different genetic architecture, with the latter being a mosaic lineage with a small tree finch-like genome, and a large *G03* haplotype. We hope that by shifting our focus towards *C. psittacula affinis*, we can keep the historical framing of our tree finch manuscript while providing a better justification of the functional/morphological analyses at the core of our study.

Minor

• Issue throughout the paper: There's a general inconsistency in the paper with whether species (both focus and otherwise) are referred to by their scientific name or their common name. I would stick with one or the other so the reader can remain familiar with what is being referred to.

To facilitate reading of the manuscript, we decided to use epithets for each lineage (L139-141), which are summarised in Table 1.

• Line 84-85: "Only one island in the archipelago, Floreana Island, harbours three species of tree finches with blunt beaks. They are small, medium and large." Provide the specific names or use other clarifying wording- the second sentence implies mere size descriptors.

We added "tree finch" at the end of the sentence to refer to their common names.

Line 132-135: "First, by using a phylogenetic network approach (Fig. 3a) we found that the woodpecker finch C. pallidus striatipecta from San Cristóbal (hereafter "pallidus San Cristóbal"), C. pallidus pallidus (from Santa Cruz), C. pallidus productus (from the western islands Isabela and Fernandina) and the mangrove finch C. heliobates from Isabela are…" This is very difficult to read. I would include a table that links each scientific name to the island they are on.

We apologize for the confusion due to different naming conventions used. Even though scientific names and island of origin of each sample were included in Figure 1c, we now add a table (Table 1) with such information and add the epithet used throughout the manuscript.

Line 397-401: Speaking on the issues with attempting to assign taxonomy. I think more emphasis should be placed on how flaws in taxonomic classification arise when we attempt to give a black-and-white assignment to a system with grey areas. Would put a sentence referencing this in the opening paragraph of the discussion.

We have added a sentence in the in the first paragraph of the discussion (L397-398).

Fig 4: C and D) Please provide indication of the threshold of statistical significance. This may necessitate showing C as a plot of -log(p) instead of raw FST. D) As it

currently reads, the figure caption for this graph makes it sound as if no matter where the line fell, it would provide you with support for your hypothesis. I would clarify where on the graph the reader should be paying attention to, and how far the line needs to be from 0 for the results to be significant.

We have now Z-transformed raw *Fst* values (Fig. 4, S8b). In this way, *Fst* is corrected by its genome-wide average and is expressed as standard deviations from the mean. In the case of Fig. 4, differentiation between the western *psittacula* and *parvulus* at *G03* is 25 sd more than its genome-wide average. Assuming a normal distribution, *ZFst* values beyond 2 lie in the 5% outer distribution (2.5% in each tail).

Fig 5: The bottom panel of this figure does not provide improved clarity from how you described the results in the section above. It may be better suited as a supplementary figure

We have improved the text in the section immediately before Fig. 5 to provide more clarity. We think that the figure nicely shows that different allele frequencies (upper panel) are underpinned by haplotypes that segregate by species, as shown in the bottom part. We have added this interpretation to the figure caption as well, and hope we have improved clarity.

Fig 6: Consider adding some indication of population and/or classification of bill size to contextualize the phylogeny.

We have now added the *Camarhynchus* icons for each tree finch lineage to help contextualise morphological differences within the tree finch phylogeny.

Line 422: "…we used 415 Darwin's finch samples (including the 77 Camarhynchus…)". Please provide in the Supplementary Materials some information, either as a list or map, of the exact numbers and locations of at least the 77 Camarhynchus samples.

We provide a map in Figure 1b with the sample size of each species in each island. The total sample size can be found in Fig. 1a.

• Line 434: "…which are analogous to…GATK 4.1"- is there a reason that these tools were used? It's fine if it's personal preference, but I'd be curious if there were specific issues with the more standard GATK tools that make alternates more suitable for these data.

Sentieon provides the GATK algorithms re-written in C to improve efficiency of the BWA/GATK pipeline, the latter of which is written in Java and is very slow. Given that our genomic dataset includes hundreds of samples, our choice was based purely because of its efficiency, but the final output is identical. We clarified this in L524-527.

• Line 471: "To perform these diversity statistics, two individuals were removed…" Particularly with the hybrid individual, was there an observable difference in these measurements with and without? If removal of an F1 is justified, what about individuals of hybrid ancestry more distantly?

This is an interesting observation but would like to clarify that only one individual was removed because of hybrid ancestry (*i.e.* the *pallidus* x *heliobates* 102Isa1940 individual). The second individual that was removed was the individual marked with a red asterisk in Fig. 3e, a putative northern *psittacula* immigrant caught on Isabela Island that was classified as western *psittacula* based on its capture location (but that belongs to the northern populations based on genetic evidence). Therefore, we removed only the individual that appeared to be a recent hybrid based on high nucleotide diversity (Fig. S2), long *heliobates* ancestry tracks (Fig. S3), intermediate phylogenetic placement (Fig. 3b) and GNN profile (Fig. 3d). As for the other individuals, we are aware that hybridization may have shaped the genomes of *Camarhynchus* finches for a long time. But together with the recent origin of this clade, it is very challenging, if not impossible, to differentiate between shared ancestry and historical gene flow with confidence. This problem holds even across different genera of Darwin's finches, which previous studies have shown they have high extent of allele sharing (Lamichhaney et al., 2015).

Line 548: "We use 348 morphological measurements…" It sounds like you used 348 individuals based on line 556, and you state you performed multiple measurements per specimen- which would make your number of measurements substantially higher than 348, correct?

We think this is a simple misunderstanding. Measurements were from 348 individuals (after removal of those stated in L663). We reworded this section to avoid any confusion.

Also, as with genetic samples, it would be advisable to provide a supplementary table with these data and specimen information, along with whether any individuals were included in both datasets (I assume not given the description of taking blood samples, but it is not entirely clear whether that rules out any of those do have a specimen associated with them if the individual later died or was collected?)

We have uploaded the biometric data to the data repository associated with this manuscript (10.6084/m9.figshare.30284983), since adding such dataset in the supplementary material would result in an overly long table.

Line 711, Reference 36: "O'connor, J.A.," C needs to be capitaliazed

We thank the reviewer for noticing this mistake, which we have now corrected.
