# Supplementary material for: A supergene and genomic mosaicism are associated with phenotypic evolution in Galápagos tree finches
Source: Nat Commun. 2026 Oct 11;17:10656. doi: 10.1038/s41467-026-78507-8 (PMC13655052; doi:10.1038/s41467-026-78507-8)
Supplement: Supplementary file 3 [file 41467_2026_78507_MOESM3_ESM.pdf]

nature portfolio

Corresponding author(s): Leif Andersson and Matteo Sebastianelli Arbelaez

Last updated by author(s): Sep 7, 2026

Reporting Summary

Nature Portfolio wishes to improve the reproducibility of the work that we publish. This form provides structure for consistency and transparency in reporting. For further information on Nature Portfolio policies, see our Editorial Policies and the Editorial Policy Checklist.

Statistics

For all statistical analyses, confirm that the following items are present in the figure legend, table legend, main text, or Methods section.

n/a	Confirmed	
☐	☒	The exact sample size (*n*) for each experimental group/condition, given as a discrete number and unit of measurement
☒	☐	A statement on whether measurements were taken from distinct samples or whether the same sample was measured repeatedly
☐	☒	The statistical test(s) used AND whether they are one- or two-sided<br>*Only common tests should be described solely by name; describe more complex techniques in the Methods section.*
☐	☒	A description of all covariates tested
☒	☐	A description of any assumptions or corrections, such as tests of normality and adjustment for multiple comparisons
☐	☒	A full description of the statistical parameters including central tendency (e.g. means) or other basic estimates (e.g. regression coefficient) AND variation (e.g. standard deviation) or associated estimates of uncertainty (e.g. confidence intervals)
☐	☒	For null hypothesis testing, the test statistic (e.g. *F*, *t*, *r*) with confidence intervals, effect sizes, degrees of freedom and *P* value noted<br>*Give P values as exact values whenever suitable.*
☒	☐	For Bayesian analysis, information on the choice of priors and Markov chain Monte Carlo settings
☒	☐	For hierarchical and complex designs, identification of the appropriate level for tests and full reporting of outcomes
☒	☐	Estimates of effect sizes (e.g. Cohen's *d*, Pearson's *r*), indicating how they were calculated

Our web collection on statistics for biologists contains articles on many of the points above.

Software and code

Policy information about availability of computer code

Data collection	No software was used for data collection.
Data analysis	The code used to analyse genotype data are available on Github at https://github.com/MatteoSebastianelli/Tree_finches. Software used for analysis:bwa-mem module v0.7.18, GATK 4.3.0.0, bcftools v1.2, vcftools 0.1.16, shapeit v4.1.3, tsinfer and tsdate 0.4.0 , pixy 2.0, GEMMA 0.98, iqtree2, vcf2phylip, SplitsTree v.6.4.13, phyml v3.3.2, twisst v.2.0, Dsuite Dtrios v.05, phytools, clustvarsel, mclust.

For manuscripts utilizing custom algorithms or software that are central to the research but not yet described in published literature, software must be made available to editors and reviewers. We strongly encourage code deposition in a community repository (e.g. GitHub). See the Nature Portfolio guidelines for submitting code & software for further information.

Data

Policy information about availability of data

All manuscripts must include a data availability statement. This statement should provide the following information, where applicable:

- Accession codes, unique identifiers, or web links for publicly available datasets
- A description of any restrictions on data availability
- For clinical datasets or third party data, please ensure that the statement adheres to our policy

Data associated with this study are available on Figshare (10.6084/m9.figshare.30284983).

Research involving human participants, their data, or biological material

Policy information about studies with human participants or human data. See also policy information about sex, gender (identity/presentation), and sexual orientation and race, ethnicity and racism.

Reporting on sex and gender	n/a
Reporting on race, ethnicity, or other socially relevant groupings	n/a
Population characteristics	n/a
Recruitment	n/a
Ethics oversight	n/a

Note that full information on the approval of the study protocol must also be provided in the manuscript.

Field-specific reporting

Please select the one below that is the best fit for your research. If you are not sure, read the appropriate sections before making your selection.

☐ Life sciences ☐ Behavioural & social sciences ☒ Ecological, evolutionary & environmental sciences

For a reference copy of the document with all sections, see nature.com/documents/nr-reporting-summary-flat.pdf

Ecological, evolutionary & environmental sciences study design

All studies must disclose on these points even when the disclosure is negative.

Study description	We conducted a study on genetic and morphological variation on Galapagos tree finches (genus Camarhynchus)
Research sample	Our study leverages two main datasets: a genomic dataset consisting of whole genome sequences for 415 birds (mostly, Darwin's finches, including 77 tree finches, and their outgroups) and a dataset of morphological measurements obtained from 348 tree finch museum specimens. More specifically, our study incorporates genomes of Loxigilla noctis and Loxigilla barbadensis (outgroups), warbler finches (Certhidea fusca and C. olivacea), vegetarian finch (Platyspiza crassirostris), Cocos finch (Pinaroloxis inornata), Geopiza difficilis, Geospiza septentrionalis, Geospiza magnirostris, Geospiza fuliginosa, Camarhynchus parvulus, Camarhynchus pauper, Camarhynchus psittacula, Camarhynchus pallidus and Camarhynchus heliobates. In total, our study incorporates samples of 269 males and 136 females that could be sexed leveraging the Z chromosome sex ration. Birds were captures using mistnets and blood was collected immediately after capture through venipuncture of the brachial vein. Upon collection of the blood sample on FTA cards, birds were released unharmed immediately after processing.
Sampling strategy	Blood samples were obtained through mistnetting.
Data collection	Blood samples for DNA sequencing were obtained through venipuncture of the brachial vein. Fieldwork was conducted by Peter Grant, Rosemary Grant, Carlos A. Valle, Lukas F. Keller and Erik Enbody
Timing and spatial scale	1973-2018. The team visited Galapagos every year from 1973 to 2012. Every year, the team would sample for a period of 1 to 3 months, mostly in the rainy season (December to March), but trips were also performed in different periods. The long term monitoring study terminated in 2012. However, the team returned in 2018 to collect samples to generate a reference genome, and additional samples were collected for short-read sequencing. The spatial scale of the sampling covers all the islands in the Galapagos archipelago.
Data exclusions	We carefully inspected the museum biometries dataset to remove measurements from individuals with missing information (n = 3), that were mislabelled or incomplete (e.g. broken body parts, n = 19) and that did not have a complete set of measurements (n = 3). This process resulted in a total of 348 individuals (19 C. heliobates, 65 C. pallidus, 145 C. parvulus, 20 C. pauper, 137 C. psittacula) that were retained for further analyses.
Reproducibility	We provide data and code used in this study on Figshare and Github
Randomization	n/a
Blinding	n/a

Did the study involve field work? ☒ Yes ☐ No

Field work, collection and transport

Field conditions	Field conditions varied from dry/semidesertic scrubland to Scalesia forest.

Location	Galapagos archipelago, Ecuador.
Access & import/export	The collection of blood samples was conducted with annual permits from the Galápagos National Parks Directorate and in accordance with its protocols and supported logistically by the Charles Darwin Research Station in Galápagos
Disturbance	Our team always tried to reduce stress levels to the birds by minimizing the processing times and handling birds according to established protocols.

Reporting for specific materials, systems and methods

We require information from authors about some types of materials, experimental systems and methods used in many studies. Here, indicate whether each material, system or method listed is relevant to your study. If you are not sure if a list item applies to your research, read the appropriate section before selecting a response.

Materials & experimental systems

n/a	Involved in the study
☒	☐ Antibodies
☒	☐ Eukaryotic cell lines
☒	☐ Palaeontology and archaeology
☐	☒ Animals and other organisms
☒	☐ Clinical data
☒	☐ Dual use research of concern
☒	☐ Plants

Methods

n/a	Involved in the study
☒	☐ ChIP-seq
☒	☐ Flow cytometry
☒	☐ MRI-based neuroimaging

Animals and other research organisms

Policy information about studies involving animals; ARRIVE guidelines recommended for reporting animal research, and Sex and Gender in Research

Laboratory animals	n/a
Wild animals	Our study involves samples from five genera of Darwin's finches: Certhidea, Platyspiza, Pinaroloxias, Geospiza and Camarhynchus, with species in the latter genus being the main focus of our study.
Reporting on sex	Of the 415 samples used in our study, 145 are female individuals. Sex was determined by comparing the relative depth of the Z chromosome in each individual to the respective autosomal depth.
Field-collected samples	Blood samples were preserved in EDTA-soaked filter paper and then stored in DRIERITE.
Ethics oversight	Ethical clearance was provided by the Princeton's University Animal Care Commitee

Note that full information on the approval of the study protocol must also be provided in the manuscript.

Plants

Seed stocks	n/a
Novel plant genotypes	n/a
Authentication	n/a
